# Supplementary material for: Plasma electrophoresis profiles of Blanding’s turtles (Emydoidea blandingii) and influences of month, age, sex, health status, and location
Source: PLoS One. 2021 Oct 14;16(10):e0258397. doi: 10.1371/journal.pone.0258397 (PMC8516212; doi:10.1371/journal.pone.0258397)
Supplement: Attachment — Submitted filename: PONE-D-21-17850_R1_reviewer 1.pdf [file pone.0258397.s004.pdf]

PLOS ONE

Plasma electrophoresis profiles of Blanding's turtles (Emydoidea blandingii) and influences of month, age, sex, health status, and location

--Manuscript Draft--

**Manuscript Number:**	PONE-D-21-17850R1
**Article Type:**	Research Article
**Full Title:**	Plasma electrophoresis profiles of Blanding's turtles (Emydoidea blandingii) and influences of month, age, sex, health status, and location
**Short Title:**	Plasma electrophoresis profiles of Blanding's turtles and influences of multiple factors
**Corresponding Author:**	Kirsten Andersson, DVM<br>University of Illinois at Urbana-Champaign<br>Urbana, Illinois UNITED STATES
**Keywords:**	Chelonians, plasma proteins, conservation, protein electrophoresis, endangered species, health monitoring, wildlife, immune response
**Abstract:**	Baseline plasma electrophoresis profiles (EPH) are important components of overall health and may aid in the conservation and captive management of species. The aim of this study was to establish plasma protein fractions for free-ranging Blanding's turtles (<i>Emydoidea blandingii <i>) and evaluate differences due to age class (adult vs. sub-adult vs. juvenile), sex (male, female, or unknown), year (2018 vs. 2019), month (May vs. June vs. July), health status, and geographical location (managed vs. unmanaged sites). Blood samples were obtained from 156 Blanding's turtles in the summer of 2018 and 129 in 2019 at two adjacent sites in Illinois. Results of the multivariate analysis demonstrated that age class, sex, year, month, health status, and geographical location all contributed to the variation observed in free-ranging populations. Adult females had the highest concentration of many protein fractions, likely associated with reproductive activity. Juveniles had lower protein concentrations. Temperature and rainfall differences between years impacted concentrations between 2018 and 2019, while May and June of both years saw higher levels in some protein fractions likely due to peak breeding and nesting season. Individuals with evidence of trauma or disease also showed increased plasma protein fractions when compared to those that were considered healthy. The two sites showed a wide/large variation over the two years. All of these factors emphasize the importance of considering multiple demographic or environmental factors when interpreting the EPH fractions. Establishing ranges for these analytes will allow investigation into disease prevalence and other environmental factors impacting this endangered species.
**Order of Authors:**	Kirsten Andersson, DVM<br>Laura Adamovicz, DVM, PhD<br>Lauren C Mumm, DVM<br>Samantha Bradley, BS<br>John C Winter, DVM<br>Gary Glowacki, MS<br>Carolyn Cray, PhD<br>Matthew C Allender, DVM, MS, PhD, Dipl ACZM
**Response to Reviewers:**	We have responded to all of the reviewer comments in the "Response to Reviewer Comments" attachment.
**Additional Information:**	
**Question**	**Response**
**Financial Disclosure**<br><br>Enter a financial disclosure statement that	Funding was provided by a private charitable foundation, Crown Family Philanthropies (https://crownfamilyphilanthropies.org/). They do not wish to have any further information disclosed. The funders had no role in study design, data collection and

describes the sources of funding for the work included in this submission. Review the submission guidelines for detailed requirements. View published research articles from *PLOS ONE* for specific examples.<br><br>This statement is required for submission and **will appear in the published article** if the submission is accepted. Please make sure it is accurate.<br><br>**Unfunded studies**<br>Enter: *The author(s) received no specific funding for this work.*<br><br>**Funded studies**<br>Enter a statement with the following details:<br>• Initials of the authors who received each award<br>• Grant numbers awarded to each author<br>• The full name of each funder<br>• URL of each funder website<br>• Did the sponsors or funders play any role in the study design, data collection and analysis, decision to publish, or preparation of the manuscript?<br>• **NO** - Include this sentence at the end of your statement: *The funders had no role in study design, data collection and analysis, decision to publish, or preparation of the manuscript.*<br>• **YES** - Specify the role(s) played.<br><br>* typeset	analysis, decision to publish, or preparation of the manuscript.
**Competing Interests**<br><br>Use the instructions below to enter a competing interest statement for this submission. On behalf of all authors, disclose any competing interests that could be perceived to bias this work—acknowledging all financial support and any other relevant financial or non-financial competing interests.<br><br>This statement is required for submission and **will appear in the published article** if the submission is accepted. Please make sure it is accurate and that any funding	The authors have declared that no competing interests exist.

sources listed in your Funding Information later in the submission form are also declared in your Financial Disclosure statement.<br><br>View published research articles from *PLOS ONE* for specific examples.<br><br>**NO authors have competing interests**<br><br>Enter: *The authors have declared that no competing interests exist.*<br><br>**Authors with competing interests**<br><br>Enter competing interest details beginning with this statement:<br><br>*I have read the journal's policy and the authors of this manuscript have the following competing interests: [insert competing interests here]*<br><br>* typeset	
**Ethics Statement**<br><br>Enter an ethics statement for this submission. This statement is required if the study involved:<br><br>• Human participants<br>• Human specimens or tissue<br>• Vertebrate animals or cephalopods<br>• Vertebrate embryos or tissues<br>• Field research<br><br>Write "N/A" if the submission does not require an ethics statement.<br><br>General guidance is provided below. Consult the submission guidelines for detailed instructions. **Make sure that all information entered here is included in the Methods section of the manuscript.**	All animal sampling was permitted by the following organizations: Department of Natural Resources (IDNR) (Scientific Collectors Permits (SCP): NH17.5065, NH18.5065, and IDNR Endangered and Threatened permits: SBT-16-062, 1199, 14-046, and 1042), the Wisconsin Department of Natural Resources (WIDNR) (SCP: SCP-SOD-004-2013 and WIDNR Scientific Research License: SRLN-18-026), and the University of Illinois Institutional Animal Care and Use Committee (Protocols: 18000 and 18165).

**Format for specific study types**<br><br>**Human Subject Research (involving human participants and/or tissue)**<br>• Give the name of the institutional review board or ethics committee that approved the study<br>• Include the approval number and/or a statement indicating approval of this research<br>• Indicate the form of consent obtained (written/oral) or the reason that consent was not obtained (e.g. the data were analyzed anonymously)<br><br>**Animal Research (involving vertebrate animals, embryos or tissues)**<br>• Provide the name of the Institutional Animal Care and Use Committee (IACUC) or other relevant ethics board that reviewed the study protocol, and indicate whether they approved this research or granted a formal waiver of ethical approval<br>• Include an approval number if one was obtained<br>• If the study involved *non-human primates*, add *additional details* about animal welfare and steps taken to ameliorate suffering<br>• If anesthesia, euthanasia, or any kind of animal sacrifice is part of the study, include briefly which substances and/or methods were applied<br><br>**Field Research**<br><br>Include the following details if this study involves the collection of plant, animal, or other materials from a natural setting:<br>• Field permit number<br>• Name of the institution or relevant body that granted permission	
**Data Availability**<br><br>Authors are required to make all data underlying the findings described fully available, without restriction, and from the time of publication. PLOS allows rare exceptions to address legal and ethical concerns. See the PLOS Data Policy and FAQ for detailed information.	Yes - all data are fully available without restriction

A Data Availability Statement describing where the data can be found is required at submission. Your answers to this question constitute the Data Availability Statement and **will be published in the article**, if accepted.<br><br>**Important:** Stating 'data available on request from the author' is not sufficient. If your data are only available upon request, select 'No' for the first question and explain your exceptional situation in the text box.<br><br>Do the authors confirm that all data underlying the findings described in their manuscript are fully available without restriction?	
**Describe where the data may be found in full sentences. If you are copying our sample text, replace any instances of XXX with the appropriate details.**<br><br>• If the data are **held or will be held in a public repository**, include URLs, accession numbers or DOIs. If this information will only be available after acceptance, indicate this by ticking the box below. For example: *All XXX files are available from the XXX database (accession number(s) XXX, XXX.*).<br>• If the data are all contained **within the manuscript and/or Supporting Information files**, enter the following: *All relevant data are within the manuscript and its Supporting Information files.*<br>• If neither of these applies but you are able to provide **details of access elsewhere**, with or without limitations, please do so. For example:<br><br>*Data cannot be shared publicly because of [XXX]. Data are available from the XXX Institutional Data Access / Ethics Committee (contact via XXX) for researchers who meet the criteria for access to confidential data.*<br><br>*The data underlying the results presented in the study are available from (include the name of the third party*	All relevant data are within the manuscript and its Supporting Information files

and contact information or URL).<br>• This text is appropriate if the data are owned by a third party and authors do not have permission to share the data.<br><br>* typeset	
Additional data availability information:	Tick here if your circumstances are not covered by the questions above and you need the journal’s help to make your data available.

Manuscript Click here to access/download;Manuscript;Manuscript.docx

Title: Plasma electrophoresis profiles of Blanding's turtles (*Emydoidea blandingii*) and influences of month, age, sex, health status, and location

Authors:

Kirsten E. Andersson, DVM,[1]* Laura Adamovicz, DVM, PhD,[1,2] Lauren E. Mumm, DVM,[1] Samantha E. Bradley,[1] John M. Winter, DVM,[1] Gary Glowacki, MS,[3] Carolyn Cray, PhD[4], Matthew C. Allender, DVM, MS, PhD, Dipl. ACZM[1]

Affiliations:

[1] Wildlife Epidemiology Laboratory, College of Veterinary Medicine, University of Illinois, Urbana, IL 61802 USA

[2] Veterinary Diagnostic Laboratory, College of Veterinary Medicine, University of Illinois, Urbana, IL 61802 USA

[3] Lake County Forest Preserve District, Libertyville, IL 60048 USA

[4] Department of Pathology & Laboratory Medicine, School of Medicine, University of Miami, Miami, FL 33136 USA

Corresponding Authors:

Kirsten E. Andersson, kkeandersson@gmail.com

Matthew C. Allender, mcallend@illinois.edu

Abstract:

Baseline plasma electrophoresis profiles (EPH) are important components of overall health and may aid in the conservation and captive management of species. The aim of this study was to establish plasma protein fractions for free-ranging Blanding's turtles (*Emydoidea blandingii*) and evaluate differences due to age class (adult vs. sub-adult vs. juvenile), sex (male, female, or unknown), year (2018 vs. 2019), month (May vs. June vs. July), health status, and geographical location (managed vs. unmanaged sites). Blood samples were obtained from 156 Blanding's turtles in the summer of 2018 and 129 in 2019 at two adjacent sites in Illinois. Results of the multivariate analysis demonstrated that age class, sex, year, month, health status, and geographical location all contributed to the variation observed in free-ranging populations. Adult females had the highest concentration of many protein fractions, likely associated with reproductive activity. Juveniles had lower protein concentrations. Temperature and rainfall differences between years impacted concentrations between 2018 and 2019, while May and June of both years saw higher levels in some protein fractions likely due to peak breeding and nesting season. Individuals with evidence of trauma or disease also showed increased plasma protein fractions when compared to those that were considered healthy. The two sites showed a wide/large variation over the two years. All of these factors emphasize the importance of considering multiple demographic or environmental factors when interpreting the EPH fractions. Establishing ranges for these analytes will allow investigation into disease prevalence and other environmental factors impacting this endangered species.

Key Words:

Chelonians, plasma proteins, conservation, protein electrophoresis, endangered species, health monitoring, wildlife, immune response

Introduction

Climate change, habitat destruction, and disease are potentially affecting the sustainability and conservation of species [1]. Wildlife health surveillance has become a critical component to maintain imperiled populations [1][2**],** but determining health is complex and involves several modalities, including physical examination, clinical pathology, pathogen presence, and contaminant exposure [3][4][5][6]. Protein electrophoresis (EPH), a component of clinical pathology investigation, has become commonly used in wildlife studies [7]. Reference interval data in several reptilian species exist and have proven to be valuable in understanding how they handle stress and disease [4][7][8][9][10][11][12][13][14][15][16]

Plasma proteins are key players in the body's innate immune response, and fluctuations in concentrations serve to indicate the presence of inflammation, infection, neoplasia, stress, or trauma [17]. In head-started red-bellied cooters (*Pseudemys rubriventris*) and captive-reared loggerhead sea turtles (*Caretta caretta*), variations in protein fractions were associated with differences in age, diets, immune stimulation, and reproductive stage [18][19], indicating baseline differences exist and responses to changes in demographic and environmental variation can be measured.

The Blanding's turtle is a semi-aquatic, long-lived species of turtle experiencing population declines over much of its range in southern and central Canada and northern United States. Individual turtles can live up to 80 years of age in the wild [20], but urban development, road mortality events, climate change, illegal poaching, and disease remain the most common threats to sustainability [21]. The Chiwaukee Prairie – Illinois Beach Lake Plain (Lake Plain) contains a population of Blanding's turtles, in which active conservation efforts are aimed at improving the long-term viability in northeastern Illinois and southeastern Wisconsin [22]. In the summer of 2015, conservation efforts incorporated physical exam and health assessment data to aid in a greater understanding of the biological threats these animals face.

The objective of this study was to establish baseline plasma protein fractions for free-ranging Blanding's turtles and to determine differences between age classes (adult vs. sub-adult vs. juvenile), sex (male vs. female), years (2018 vs. 2019), months (May vs. June vs. July), health status, and geographical location. It was hypothesized that total protein levels would be higher in adults than subadults and juveniles, higher in females than males, higher in May than in June and July, and higher in unhealthy turtles than healthy turtles.

Materials and Methods

Study sites

Blanding's turtles were sampled from three sites within the Lake Plain including Spring Bluff-Chiwaukee Prairie (SBCP), the managed site, and the North and South units of Illinois Beach State Park (IBSP), the unmanaged sites. SBCP consists of approximately 535 acres of high-quality coastal dune and swale habitat along the coast of Lake Michigan in Illinois and Wisconsin, whereas IBSP consist of 4,160 acres of dune, prairie, oak-savannah, and wetland habitats along 6.5 miles of coastline [22]. Management of mesopredators is also more robust in SBCP than IBSP, with studies focusing on camera trap surveillance for predator presence throughout the year as well as nest predation rates [23].

Capture Methods

Turtles were captured with the aid of radiotelemetry, hoop net trap, or incidentally by hand. Radiotelemetry is a three-part system using a radio transmitter, a radio antenna, and a radio receiver. The transmitter is attached to the turtle's shell, which transmits a signal to the antenna and correlates to a beeping produced by the receiver that gets louder as the animal gets closer [23][24]**.** Hoop traps were placed in marsh waters and areas that were characteristic of

Blanding's habitat or locations near previous Blanding's turtle capture sites. These traps were checked every 24 hours and remained in the same location for up to five days. Telemetry, traps, and incidental captures were used in both field sites. Many turtles, especially those equipped with a radio transmitter, were sampled up to two times per summer.

Physical examination and Sample collection

Each turtle was assigned a permanent ID, marking the shell with a notch code unless previously marked as well as inserting a pit tag (microchip) under the skin, and mass, sex, and age class were recorded. Sex was classified as male, female, or unknown. Sex of head-starte[illegible] turtles, which are clutches deposited in captivity from free-range females, was known due to established incubation temperatures, as males were incubated at 26.5°C and females at 31.0°C [23][25]. The sex of adults was determined based on plastron concavity [26]. The sex of most sub-adults and juveniles was estimated based on position of cloacal opening [27]. Wild-born individuals were classified as unknown sex when a confident determination could not be made. Age class was characterized as juvenile (<250 grams), sub-adult (250-750 grams), or adult (>750 grams). Blanding's turtles were deemed sexually mature at 750 grams ~~and over by the Lake County Forest Preserve District (LCFPD)~~ based on the lightest fertile female noted. This methodology is based on a previously published study by Mumm~~,~~ et~~l~~ al [24]. Body fat percentage (FP) was calculated using a published calculation from the relationship of carapace length and mass [28]. Physical examinations were performed noting visual appearance of the eyes, nose, oral cavity, ears, legs, digits, shell, integument, and cloaca. Gravidity was assessed using digital palpation of the prefemoral fossa. For the purpose of statistical analysis, females were classified as gravid if they had palpable eggs or if they had nested within one week of sampling. Nesting was determined either by observation of nesting behavior or the lack of

palpable eggs after having previously been confirmed gravid. Turtles were classified as either “apparently healthy” or “unhealthy” based on the presence of clinically significant physical exam abnormalities, including open fractures or wounds; ocular, oral or nasal discharge; depressed mentation; missing nails, digits, or appendages; and evidence carapace/plastron damage.

Whole blood was collected from the sub-carapacial sinus via 22-gauge or 25-gauge needle, subject to the size of the individual. No more than 0.6% of body weight of whole blood was drawn and placed into lithium-heparinized plasma separator tubes. Blood samples were placed on ice in a cooler for one to five hours depending on time of collection until returning to the lab each afternoon. Total protein (TP) was estimated using refractometry. Whole blood samples were centrifuged at 4,185 g for 10 minutes, stored at -20°C for one to four months, and shipped on dry ice to the University of Miami at the end of the field season. All individuals were released at coordinates of capture. All animal sampling was permitted by the following organizations: Department of Natural Resources (IDNR) (Scientific Collectors Permits (SCP): NH17.5065, NH18.5065, and IDNR Endangered and Threatened permits: SBT-16-062, 1199, 14-046, and 1042), the Wisconsin Department of Natural Resources (WIDNR) (SCP: SCP-SOD-004-2013 and WIDNR Scientific Research License: SRLN-18-026), and the University of Illinois Institutional Animal Care and Use Committee (Protocols: 18000 and 18165).

Protein Electrophoresis

Plasma samples were analyzed according to the procedure provided by the Helena SPIFE 3000 system with the use of Split Beta gels (Helena Laboratories, Inc., Beaumont, Texas 77707, USA). Results were produced after gel scanning and analysis by Helena software. Fraction delimits were placed as previously demonstrated for other reptiles [10]. Plasma protein fractions were divided into the following six fractions: a fraction migrating in the prealbumin region

("prealbumin"), albumin, alpha 1 globulins, alpha 2 globulins, beta globulins, and gamma globulins (Fig 1). Percentages for each fraction were determined by this software, which gave the relative value, and absolute values (g/L) for each fraction were obtained by multiplying the percentage by the TP concentration. The albumin:globulin ratio (A:G) was calculated by dividing the sum of albumin and ~~pre~~albumin by the sum of the globulin fractions.

Fig 1. Electrophoretogram comparison of plasma protein fractions.

A visual representation of the plasma protein fraction distribution amongst three representatives from each age classes. The top panel shows the protein as they appeared on the gel following electrophoresis, and the bottom panel shows a graphic representation of the concentration differences. Juvenile (A), sub-adult (B), and adult(C).

Statistical analysis

All statistical analyses were conducted in R version 3.6.3 [29] at an alpha level of 0.05, unless otherwise specified. Data distributions were assessed using histograms and the Shapiro-Wilk test and transformation was pursued, if necessary, to meet modeling assumptions.

Protein electrophoresis fractions were modeled using general linear mixed models with the lme4 and lmerTest packages [30][31]. Fixed effects included spatiotemporal variables (year, month, location), demographic variables (sex, age class, gravidity), and health variables (body condition, presence/absence of physical exam abnormalities), while turtle ID was included as a random effect. Post-hoc testing was performed using the lsmeans package with a Tukey correction to control for multiple statistical tests [32]. Fixed effects with univariable p-values < 0.15 were considered in multivariable models testing specific biological hypotheses about the effects of spatiotemporal, demographic, and health variables on EPH values. Variance inflation

factors were evaluated for multivariable models to identify and exclude highly collinear (VIF > 10) predictor variables (function vif, package car) [33]. Candidate model sets were constructed for each EPH fraction and ranked using an information-theoretic approach [34]. Figures were constructed using the ggeffects package [35]. Spatial clustering of EPH fractions was modeled in ArcGIS version 10.6 using hot spot analysis with the Gettis-Ord Gi* statistic from the spatial statistics toolbox. Hot spots are areas with higher plasma protein concentrations, while cold spots are areas with lower plasma protein concentrations.

Coefficients of variation were determined for each EPH fraction using data from apparently healthy turtles evaluated at multiple time points (CV_I) and only a single time point (CV_G). The index of individuality (II) was calculated as CV_I / CV_G and was used to infer the need for subject-based vs. population-based reference intervals [36][37]. When the II is < 0.6, subject-based reference intervals was used, while an II > 1.4 population-based reference intervals were created (Harris, 1974). When the II was between 0.6 and 1.4, population-based reference intervals were used [37][38]. Reference change values (RCV) were calculated for each EPH analyte using a published formula [36][37].

Population-based reference intervals were also determined for each analyte using the nonparametric method (referenceIntervals package) [39], according to American Society for Veterinary Clinical Pathology guidelines [36]. Outliers were identified and excluded using Horn's method [40]. Ninety percent confidence intervals were generated around the limits of each reference interval using nonparametric bootstrapping with 5000 replicates. The population-based reference interval dataset included only turtles sampled once, and a randomly-selected single time point (https://www.random.org/) from serially-sampled individuals.

Results

Two hundred and eighty-five samples were collected from 215 individual turtles. Fifty animals were sampled two times approximately one year apart, and ten animals were sampled three times – twice within the 2018 active season and a third time in 2019. One hundred fifty-six samples were obtained in 2018, while 129 were collected in 2019. One hundred seventy-eight samples were collected at SBCP, 51 were from IBSP North Unit, 54 were from IBSP South Unit, and two individuals did not have location data recorded. Samples were collected in May (N = 129), June (N = 120), and July (N = 36) from 171 adults, 83 sub-adults, and 31 juveniles. Sex distribution included 174 females, 75 males, and 36 turtles of unknown sex. Twenty-two females were gravid.

Blood samples were collected from the subcarapacial sinus due to its relative ease of access and minimal restraint requirement in a field setting. Grossly hemolyzed or lymph contaminated samples were removed from the study, although microscopic hemolysis cannot be ruled out. The timing of sampling relative to food consumption is unknown, so post-prandial changes, such as lipemia, could impact results. Physical examination was largely unremarkable for most individuals. Clinical signs of upper respiratory disease (URD), including oculonasal discharge, blepharoedema, and/or oral plaques were present in eleven turtles. Integumentary abnormalities including abrasions, lacerations, and/or nodules were present in 28 turtles. Appendicular abnormalities including abnormal nails and/or missing digits, feet, limbs, or tail tips were present in 42 animals. Cloacal abnormalities consisting of erythema, swelling, and/or discharge were present in 15 animals. Shell abnormalities involved the carapace (erosions – 25, predator injury - 8) and plastron (erosions – 99, predator injury - 12). In total, 30 turtles had active physical exam abnormalities significant enough to compromise health, and these individuals were excluded from the reference interval dataset.

All absolute EPH parameters varied by year, with TP (effect size = 3.70g/L, 95% CI = 1.60 – 5.90g/L, p = 0.01), albumin (effect size = 1.20g/L, 95% CI = 0.70 – 1.70g/L, p < 0.01), alpha 1 globulins (effect size = 0.86g/L, 95% CI = 0.60 – 1.10g/L, p < 0.01) alpha 2 globulins (effect size = 0.63g/L, 95% CI = 0.26 – 1.00g/L, p = 0.01), and gamma globulins (effect size = 4.30g/L, 95% CI = 3.70 – 4.80g/L, p < 0.01) higher in 2018 than 2019, and A:G (effect size = 0.02, 95% CI = 0.01 – 0.03, p = 0.02), prealbumin (effect size = 0.67g/L, 95% CI = 0.58 – 0.77g/L, p < 0.01), and beta globulins (effect size = 2.60g/L, 95% CI = 1.60 – 3.60g/L, p = 0.01) higher in 2019 than 2018. The relationships between relative EPH fraction and year were similar, except there was no significant association between relative alpha 2 globulins and year (p = 0.34).

The effects of location on absolute EPH parameters depended on year (significant Year*Location interaction, p < 0.05) for TP, A:G, prealbumin, albumin, and gamma globulins (Fig 2). A significant year*location effect was also identified for relative prealbumin (Fig 3). Location influenced absolute alpha 2 globulins (p = 0.02), absolute beta globulins (p = 0.04), and relative gamma globulins (p = 0.01) independent of year, while it was not a statistically significant predictor of absolute alpha 1 globulins, relative albumin, relative alpha 1 globulins, relative alpha 2 globulins, or relative beta globulins (p > 0.05). In addition to the site-level effects identified using general linear models, finer-scale clusters of high and low absolute EPH values were identified using spatial modeling (Figs 4). The location of these spatial clusters varied between years, and differences occurred both between and within study sites, especially in turtles sampled at SBCP in 2019 (Fig 4).

Fig 2. Model predictions for plasma protein electrophoresis values based on year and location.

Model predictions with 95% confidence intervals for plasma protein electrophoresis values in free-living Blanding's turtles (*Emydoidea blandingii*) based on year and location. Model estimates were produced by top-ranking general linear mixed models (see Table 2). SB = Spring Bluff-Chiwaukee Prairie, IB-N = Illinois Beach North, IB-S = Illinois Beach South.

Fig 3. Model predictions for plasma protein electrophoresis values based on year, location, month, and BCI.

Model predictions with 95% confidence intervals for plasma protein electrophoresis values in free-living Blanding's turtles (*Emydoidea blandingii*) based on year, location, month, and body condition index (BCI). Model estimates were produced by top-ranking general linear mixed models (see Table 2).

Fig 4. 2018 and 2019 spatial clusters of plasma protein electrophoresis values in free-living Blanding's turtles (*Emydoidea blandingii*).

Study site map displaying hot and cold spots for each analyte in 2018 and 2019. Hot spots are areas with higher plasma protein concentrations, while cold spots are areas with lower plasma protein concentrations. SBCP, the managed site, is the northernmost territory on the map followed by the North unit of IBSP as the central territory and the South unit of IBSP as the southernmost territory. Evaluated using Gettis-Ord Gi* models. Map obtained from USGS National Map Viewer. TP = total protein (g/L), PreALB = prealbumin (g/L), ALB = albumin (g/L), Alpha1 = alpha 1 globulins (g/L), Alpha2 = alpha 2 globulins (g/L), Beta = beta globulins (g/L), Gamma = gamma globulins (g/L), A:G = albumin / globulin.

A:G ($p < 0.01$), absolute albumin ($p = 0.01$), relative albumin ($p < 0.01$), and relative gamma globulins ($p = 0.02$) differed by month, while the effects of month on absolute and

relative prealbumin concentration depended on year (significant Month*Year interaction) (Fig 2, Tables 1 and 2). Specifically, relative gamma globulins were significantly higher in May compared to June while A:G, absolute albumin, and relative albumin were significantly higher in June compared to both May and July. Relative and absolute prealbumin followed a similar trend to A:G and albumin in 2019, but values were not significantly different by month in 2018.

Table 1. Protein electrophoresis values that vary by month and the presence of plastron abnormalities in free-living Blanding's turtles (*Emydoidea blandingii*). Model estimates were produced by top-ranking general linear mixed models (see Table 2).

Analyte	Level	Model Estimate	SE	Contrast	Difference	95% CI	P - value
**Month**							
**Albumin/Globulin**	May	0.27	0.01	May vs. June	-0.04	-0.06, -0.02	< 0.01
	June	0.31	0.01	June vs. July	0.04	0.01, 0.06	0.01
	July	0.27	0.01	May vs. July	-0.01	-0.03, 0.02	1.00
**Albumin (g/L)**	May	6.00	0.40	May vs. June	-1.40	-2.20, -0.60	0.01
	June	7.40	0.40	June vs. July	1.33	0.20, 2.30	0.01
	July	6.10	0.50	May vs. July	-0.10	-1.10, 0.90	1.00
**Albumin (%)**	May	19.00	0.64	May vs. June	-2.10	-3.30, -0.95	0.01
	June	21.10	0.44	June vs. July	1.80	0.33, 3.20	0.01
	July	19.40	0.68	May vs. July	-0.36	-1.70, 0.98	0.80
**Gamma Globulins (%)**	May	17.20	0.41	May vs. June	1.31	0.20, 2.40	0.02
	June	15.90	0.39	June vs. July	-0.81	-2.30, 0.70	0.40
	July	16.70	0.58	May vs. July	0.51	-0.97, 2.00	0.70
**Plastron**							
**Beta Globulins (g/L)**	Normal	11.30	0.70	N vs. E	-1.60	-2.90, -0.40	0.01
	Erosions	12.90	0.70	E vs. I	-2.10	-4.90, 0.70	0.15
	Injury	15.00	1.40	N vs. I	-3.70	-6.60, -0.90	0.01
**Beta Globulins (%)**	Normal	35.60	0.80	N vs. E	-0.27	-1.64, 1.10	0.70
	Erosions	35.90	0.90	E vs. I	-3.33	-6.40, -0.28	0.03
	Injury	39.20	1.50	N vs. I	-3.60	-6.60, -0.61	0.02

SE = standard error. N = normal. E = erosions. I = injury.

Table 2. Top models for predicting protein electrophoresis values in free-living Blanding's turtles (*Emydoidea blandingii*) based on Akaike's information criterion, corrected for sample size (AIC_c).

Analyte	Model	N	K	AIC_c	w_i
**Total Protein (g/L)**	Y * L + BCI + Integ + App	265	11	748.00	0.98
**Albumin / Globulin**	Y * L + M + Sex + Age + Cloaca	279	15	-825.40	0.98
**Prealbumin (g/L)**	Y * L + Y * M + URD	279	13	-995.90	0.80
**Prealbumin (%)**	Y * L + Y * M + URD	279	13	-1534.60	0.69
**Albumin (g/L)**	Y * L + M + App + Gravid	278	12	-7.70	0.57
**Albumin (%)**	Y + M + Age + Sex + Gravid	279	11	-1154.10	0.94
**Alpha 1 Globulins (g/L)**	Y + Age + Carapace	279	10	-423.10	0.58
**Alpha 1 Globulins (%)**	Y + Age + Carapace	279	10	-1232.30	0.80
**Alpha 2 Globulins (g/L)**	Y + L + Age	283	8	-157.30	0.76
**Alpha 2 Globulins (%)**	Sex + Age + Gravid	280	8	-1207.10	0.95
**Beta Globulins (g/L)**	Y + L + Sex + BCI + Integ + Plastron + App	265	15	307.80	0.99
**Beta Globulins (%)**	Y + Age + Sex + Plastron + Gravid	280	13	-891.00	0.99
**Gamma Globulins (g/L)**	Y * L + Age + BCI + App	266	12	-34.80	0.99
**Gamma Globulins (%)**	Y + L + M + Age	283	10	-1129.80	0.86

Y = year, L = location, M = month, BCI = body condition index, Integ = integument, App = appendages, URD = upper respiratory disease.

Age class influenced A:G and all relative EPH fractions except prealbumin; it was also found to be an important predictor of absolute alpha 1 globulins, alpha 2 globulins, and gamma globulins (Table 3). Juvenile turtles had the highest values for A:G, relative albumin, relative alpha 1 globulins, and absolute alpha 1 globulins. Subadults had the highest values for relative and absolute alpha 2 globulins. Adults had the highest values for relative beta globulins, relative gamma globulins, and absolute gamma globulins.

Table 3. Protein electrophoresis values that vary by age class in free-living Blanding's turtles (*Emydoidea blandingii*). Model estimates were produced by top-ranking general linear mixed models (see Table 2). SE = standard error. Ad = adult. SA = sub-adult. Juv = juvenile.

Analyte	Level	Model Estimate	SE	Contrast	Difference	95% CI	P - value
**Albumin/Globulin**	Adult	0.25	0.01	Ad vs. Juv	-0.07	-0.11, -0.04	< 0.01
	Sub-Adult	0.28	0.01	Ad vs. SA	-0.04	-0.06, -0.01	0.01
	Juvenile	0.32	0.02	SA vs. J	-0.04	-0.07, -0.01	0.03
**Albumin (%)**	Adult	17.90	0.41	Ad vs. Juv	-3.80	-5.80, -1.80	<0.01
	Sub-Adult	19.80	0.63	Ad vs. SA	-1.80	-3.10, -0.56	0.01
	Juvenile	21.80	0.87	SA vs. J	-2.00	-4.00, 0.02	0.05
**Alpha 1 Globulins (g/L)**	Adult	1.60	0.10	Ad vs. Juv	-2.40	-2.90, -1.80	< 0.01
	Sub-Adult	2.70	0.10	Ad vs. SA	-1.00	-1.40, -0.70	< 0.01
	Juvenile	4.00	0.20	SA vs. J	-1.30	-1.90, -0.80	< 0.01
**Alpha 1 Globulins (%)**	Adult	5.20	0.30	Ad vs. Juv	-9.30	-10.60, -8.00	< 0.01
	Sub-Adult	8.70	0.40	Ad vs. SA	-3.50	-4.40, -2.60	< 0.01
	Juvenile	14.50	0.60	SA vs. J	-5.80	-7.20, -4.40	< 0.01
**Alpha 2 Globulins (g/L)**	Adult	5.10	0.20	Ad vs. Juv	0.10	-0.70, 0.90	1.00
	Sub-Adult	5.80	0.30	Ad vs. SA	-0.70	-1.30, -0.20	0.01
	Juvenile	5.00	0.40	SA vs. J	0.80	0.04, 1.70	0.04
**Alpha 2 Globulins (%)**	Adult	14.30	0.37	Ad vs. Juv	-1.10	-2.90, 0.60	0.30
	Sub-Adult	16.40	0.50	Ad vs. SA	-2.10	-3.20, -1.00	< 0.01
	Juvenile	15.50	0.78	SA vs. J	1.00	-0.90, 2.80	0.40
**Beta Globulins (%)**	Adult	41.70	0.79	Ad vs. Juv	9.61	6.40, -12.83	< 0.01
	Sub-Adult	34.40	0.97	Ad vs. SA	7.28	5.40, -9.16	< 0.01
	Juvenile	32.10	1.50	SA vs. J	2.33	-0.98, 5.65	0.20
**Gamma Globulins (g/L)**	Adult	7.00	0.30	Ad vs. Juv	2.00	0.60, 3.40	0.01
	Sub-Adult	6.60	0.40	Ad vs. SA	0.30	-0.80, 1.40	0.85
	Juvenile	5.00	0.50	SA vs. J	1.70	0.60 – 3.00	0.01

**Gamma Globulins (%)**	Adult	19.00	0.31	Ad vs. Juv	6.00	4.37, 7.63	< 0.01
	Sub-Adult	18.00	0.45	Ad vs. SA	0.98	-0.22, 2.20	0.13
	Juvenile	13.00	0.65	SA vs. J	5.00	3.36, 6.68	< 0.01

SE = standard error. Ad = adult. SA = sub-adult. Juv = juvenile.

Sex influenced the A:G, relative albumin, relative alpha 2 globulins, relative beta globulins, and absolute beta globulins (Table 4). Specifically, male turtles had higher A:G, relative albumin, and relative alpha 2 globulins, while females had higher absolute and relative beta globulin values. Gravid females had higher relative beta globulins and lower A:G, absolute and relative albumin, and alpha 2 globulins.

Table 4. Protein electrophoresis values that vary by sex and gravidity in free-living Blanding's turtles (*Emydoidea blandingii*).

Model estimates were produced by top-ranking general linear mixed models (see Table 2). SE = standard error.

Predictor	Analyte	Level	Model Estimate	SE	Difference	95% CI	P - value
**Sex**	**Albumin (%)**	Male	20.40	0.60	1.21	0.31 - 2.11	0.01
		Female	19.20	0.50			
	**Albumin/Globulin**	Male	0.30	0.01	0.02	0.01 - 0.04	0.01
		Female	0.27	0.01			
	**Alpha 2 Globulins (%)**	Male	16.20	0.50	1.60	0.72 - 2.40	0.01
		Female	14.60	0.43			
	**Beta Globulins (g/L)**	Male	12.30	0.90	1.80	0.50 – 3.20	0.01
		Female	14.10	0.80			
	**Beta Globulins (%)**	Male	34.50	0.97	3.04	1.56 - 4.53	< 0.01
		Female	37.60	0.92			
**Gravidity**	**Albumin/Globulin[a]**	Non-Gravid	0.31	0.01	0.03	0.02 - 0.06	0.04
		Gravid	0.27	0.02			
	**Albumin (g/L)**	Non-Gravid	7.10	0.30	1.20	0.03 -2.30	0.04
		Gravid	6.00	0.60			
	**Albumin (%)**	Non-Gravid	21.00	0.32	2.42	0.88 - 3.95	0.01
		Gravid	18.60	0.84			
	**Alpha 2 Globulins (%)**	Non-Gravid	16.40	0.28	1.90	0.63 - 3.24	0.01
		Gravid	14.40	0.70			
	**Beta Globulins (%)**	Non-Gravid	34.40	0.73	3.40	1.07 - 5.71	0.01
		Gravid	37.80	1.30			

SE = standard error.

[a] Gravidity was not included in the top-ranking model for Albumin/Globulin due to confounding with the "cloaca" variable, but considered separately it is significantly associated with Albumin/Globulin

Multiple EPH fractions were associated with health predictors. Body condition was negatively associated with TP, absolute beta globulins, and absolute gamma globulins (Fig 2). Turtles with predator injuries or erosions of the plastron had higher relative and absolute beta globulins than those with normal plastrons (Table 1). Abnormalities of the appendicular system were associated with higher TP and absolute albumin, beta globulins, and gamma globulins (Table 5). Integumentary abnormalities were associated with lower TP and absolute beta globulins (Table 5). Turtles with upper respiratory disease had lower absolute and relative prealbumin than those without (Table 5). Finally, cloacal abnormalities were associated with a lower A:G (Table 5). Top models for each EPH fraction tended to include spatiotemporal, demographic, and health predictors, highlighting the influence that each of these components has on the distribution of blood proteins in Blanding's turtles (Table 2).

Table 5. Protein electrophoresis values that vary based on the presence of physical examination abnormalities in free-living Blanding's turtles (*Emydoidea blandingii*). Model estimates were produced by top-ranking general linear mixed models (see Table 2). Top models for relative and absolute alpha 1 globulins contained "carapace", however no contrasts for this predictor were significant therefore not reported.

Predictor	Analyte	Level	Model Estimate	SE	Difference	95% CI	P - value
**Appendages**	**Total Protein (g/L)**	Normal	31.90	1.10	5.30	1.90 – 8.06	0.01
		Abnormal	37.10	1.80			
	**Albumin (g/L)**	Normal	6.10	0.30	9.00	0.10 -1.70	0.03
		Abnormal	7.00	0.50			
	**Beta Globulins (g/L)**	Normal	12.10	0.70	2.20	0.50 – 3.80	0.01
		Abnormal	14.20	1.00			
	**Gamma Globulins (g/L)**	Normal	5.50	0.30	1.40	0.60 – 2.10	0.01
		Abnormal	6.80	0.40			
**Integument**	**Total Protein (g/L)**	Normal	31.90	1.10	4.60	0.70 – 8.50	0.02
		Abnormal	27.20	2.00			
	**Beta Globulins (g/L)**	Normal	14.10	0.70	2.00	0.20 – 3.80	0.03
		Abnormal	12.20	1.10			
**Upper Respiratory Disease**	**Prealbumin (g/L)**	Absent	0.58	0.03	0.27	0.03 - 0.52	0.03
		Present	0.31	0.12			
	**Prealbumin (%)**	Absent	1.88	0.13	1.04	0.11 - 1.97	0.03
		Present	0.84	0.48			
**Cloaca**	**Albumin/Globulin**	Normal	0.31	0.01	0.04	0.01 - 0.07	0.01
		Abnormal	0.26	0.02			

SE = standard error.

Reference intervals were constructed in both a subject-based and population-based manner for each EPH analyte (Table 6). Based on the index of individuality, subject-based reference intervals are superior for absolute and relative alpha 1 globulins, absolute beta globulins, and relative albumin in Blanding's turtles. The remainder of the II values fell between 0.6 and 1.4, indicating that population-based reference intervals were employed and interpreted with caution for all other fractions.

Table 6. Summary data including data distribution, measure of central tendency (mean for normally distributed variables, median for non-normally distributed variables), measure of dispersion (standard deviation for normally distributed variables, 10th – 90th percentiles for non-normally distributed variables), and reference intervals for plasma protein electrophoresis in free-living, apparently healthy Blanding's turtles (*Emydoidea blandingii*).

Analyte	N	Dist	CT	Disp	Min	Max	Reference Interval	90% CI LB	90% CI UB	II	RCV (%)	Sub vs. Pop
**Total Protein (g/L)**	193[a]	NG	31.00	18.00 – 47.50	13.00	56.00	15.70 – 52.30	15.40 – 17.60	48.60 – 53.60	0.68	62.70	Pop
**Albumin/Globulin**	195[b]	G	0.29	0.07	0.14	0.46	0.17 - 0.43	0.16 - 0.19	0.40 - 0.46	0.66	41.20	Pop
**Prealbumin (g/L)**	196	NG	0.40	0.05 – 1.20	0.00	2.30	0.00 – 2.00	0.00 - 0.10	1.80 – 2.30	0.90	237.00	Pop
**Albumin (g/L)**	196	NG	6.30	3.40 – 9.90	1.90	15.60	2.50 – 13.40	2.10 – 3.00	12.90 - 15.00	0.66	74.40	Pop
**Alpha 1 Globulins (g/L)**	196	NG	1.90	1.00 – 4.30	0.70	11.60	0.80 – 5.70	0.70 - 0.80	4.10 – 6.10	0.54	88.40	Sub
**Alpha 2 Globulins (g/L)**	196	NG	4.70	2.70 – 7.40	1.70	11.20	2.10 – 10.00	1.90 – 2.50	9.60 - 10.70	0.67	70.80	Pop
**Beta Globulins (g/L)**	196	NG	10.80	5.04 - 17.90	3.40	30.00	4.00 - 25.70	3.30 – 4.40	21.80 - 29.90	0.56	66.70	Sub
**Gamma Globulins (g/L)**	195[c]	NG	5.60	2.80 – 10.70	1.40	16.50	1.70 - 14.10	1.30 – 1.90	12.30 - 15.70	0.99	144.00	Pop
**Prealbumin (%)**	196	NG	1.00	0.00 - 4.20	0.00	10.10	0.00 - 6.70	0.00 – 1.00	4.90 - 7.80	0.93	266.00	Pop
**Albumin (%)**	195[d]	G	20.30	3.80	13.00	31.00	14.00 – 29.00	13.00 – 15.00	27.00 – 31.00	0.59	29.90	Sub
**Alpha 1 Globulins (%)**	196	NG	6.00	4.00 – 14.00	2.70	28.00	3.00 – 23.00	2.90 - 3.30	19.00 - 28.90	0.34	51.80	Sub
**Alpha 2 Globulins (%)**	195[e]	NG	16.00	11.00 - 19.70	9.00	24.00	10.00 - 21.70	9.70 – 11.00	20.50 - 23.30	0.62	31.20	Pop
**Beta Globulins (%)**	196	NG	35.80	25.00 - 49.10	18.00	59.10	20.00 - 55.20	19.00 – 21.00	51.90 - 57.70	0.82	59.20	Pop
**Gamma Globulins (%)**	196	NG	19.00	11.30 – 28.00	8.00	37.00	9.700 - 32.10	9.40 – 10.00	31.20 - 34.10	1.20	114.00	Pop

Dist = distribution, NG = Non-Gaussian, G = Gaussian, CT = measure of central tendency, Disp = measure of dispersion, CI = confidence interval, LB = lower bound of reference interval, UB = upper bound of reference interval, II = index of individuality, RCV = reference change value, Sub = subject-based refence interval recommended, Pop = population-based reference interval recommended.

[a] Outliers removed: 10.00, 71.00, 74.00 g/L

[b] Outliers removed: 0.51

[c] Outliers removed: 22.6 g/L

[d] Outliers removed: 11%

[e] Outliers removed: 26%

Discussion

We set out to describe baseline plasma protein fractions in a well-studied population of Blanding's turtles in northeastern Illinois and southeastern Wisconsin and observed EPH fractions varied significantly based on spatiotemporal, demographic, and health factors. Understanding how reptile clinical pathology values correlate to landscape changes is important for contextualizing health assessments and evaluating ecosystem wellness [1][2][5][6].

We documented several statistically significant inter-annual differences in Blanding's turtle total protein and EPH fractions. These changes may be attributable to fluctuation in climactic variables that influence turtle metabolism and resource availability. Temperature is a key determinant of metabolic rates in ectotherms, including reptiles [41][42], and previous studies in loggerheads (*Caretta caretta*) and green turtles (*Chelonia mydas*) ~~observed a~~ negative correlation with the A:G and environmental temperature [16]. In Lake County, the average air temperatures in May (60.4°F/15.8°C) and June (66.4°F/19.1°C) of 2018 were warmer than those in May (54.7°F/12.6°C) and June (64.2°F/17.9°C) of 2019 [43]. Similar to the temperature-associated protein changes in sea turtles, Blanding's turtle TP, albumin, alpha 1 and alpha 2 globulins, and gamma globulin concentrations were greater in 2018, while prealbumin, beta globulin, and A:G were greater in 2019. It is plausible that turtles would be more active, consume more food items, initiate reproductive activity, and mount immune responses more efficiently at higher temperatures, all of which may increase circulating protein concentrations and contribute to the observed inter-annual variability in EPH fractions [41].

~~Temperature, however, is not the only environmental factor that differed between years.~~ Rainfall was also greater during May and June of 2018 (30.2 inches total) compared to the same time period in 2019 (18.76 inches total), while humidity was a bit more consistent between years (77.2% average relative humidity in May and June of 2018 and 80.75% average relative

humidity in May and June of 2019) [43]. Water availability and humidity both influence behavioral thermoregulation in ectotherms and can modify their activity levels in complex ways [44]. Increased activity secondary to rainfall may create more opportunities for antigenic stimulation and contribute to changes in food consumption, reproductive behaviors, and other physiologic processes with a resultant increase in plasma protein concentrations [44]. While the effects of some climactic variables on ectotherm physiology have been at least partially characterized, many other environmental variables may also impact resource availability and overall wellness. Additional research is needed to determine the underlying environmental causes of temporal variability in reptile protein electrophoresis values. It is likely that inter-annual differences in protein electrophoresis values also exist for other reptiles [45]. Unfortunately, direct comparison to existing literature is difficult, because although some multi-year chelonian EPH studies exist [4][10][16][18][46][47][48][49][50][51][52], the possibility of inter-annual variation in EPH values is infrequently assessed. Our findings indicate that future EPH studies in reptiles should consider the potential for significant inter-annual effects.

TP and absolute EPH fractions differed by location in Blanding's turtles, similar to eastern box turtles (*Terrapene carolina carolina*) [4], alligator snapping turtles (*Macrochelys temminckii*) [46] and green turtles [45]. SBCP and IBSP vary significantly in their habitats despite their close proximity. SBCP offers a coastal dune and swale habitat that has been managed and preserved since 2004, whereas IBSP includes adjacent north (N) and south (S) units containing dune, prairie, oak-savannah and wetland habitats, with limited management prior to 2017 [22]. The effect of location on EPH parameters depended on the year; and the differences in EPH values between the study sites and years were rarely consistent. The only consistent location-related differences between 2018 and 2019 were the cold spots at the northern end of SBCP, potentially indicating a more consistent health status for turtles occupying

this area. Inter-annual fluctuation in TP and EPH fractions between and within the different study sites may indicate transient, localized changes in health status with unclear management implications. Focused, longitudinal assessment of telemetered individuals within these areas will be useful to identify biotic and abiotic factors associated with acute changes in EPH parameters. Our findings indicate that it is important to consider both location and time at multiple different scales in order to obtain a nuanced understanding of the drivers of turtle health status.

Several Blanding's turtle plasma protein values were affected by month; specifically, A:G, absolute albumin, relative albumin, and both absolute and relative prealbumin values peaked in June, while relative gamma globulins were highest in May. Similar patterns have been documented in other chelonians including Hermann's tortoises (*Testudo hermanni*) [53], alligator snapping turtles [46], and eastern box turtles [4]. These changes may be attributed to increased interactions with other turtles during the mating season and/or reproductive physiology. In the Lake Plain, Blanding's turtle mating season occurs from March to May, while nesting season typically begins in June. Turtles are more likely to interact with each other and potentially transmit pathogens such as *Emydoidea* herpesvirus 1 during mating season [54], which may contribute to an increase in gamma globulins (i.e. immunoglobulins). Alternatively, the increased gamma globulin concentration in May could be secondary to vitellogenesis, as the release of estrogen stimulates hyperglobulinemia in chelonians [55][56]. Elevated albumin concentrations in June may be attributable to dehydration associated with prolonged overland trips and nesting-associated exertion, similar to previous reports in sea turtle species [48][57][58][59]. Studies in other chelonian and lizard species have documented elevations in albumin and total proteins during the summer months and have correlated these elevations to the increased food consumption and reproductive activity in this time period [4][60][61].

Gravid females had higher relative beta globulins and lower A:G, absolute and relative albumin, and alpha 2 globulins. As reviewed above, gravid reptiles can develop hyperglobulinemia during vitellogenesis in response to estrogen [62]. A study conducted in pond sliders (*Trachemys scripta*) demonstrated that estrogen also downregulates albumin, which may be a factor in the lower albumin concentrations observed in gravid female Blanding's turtles [63]. Higher globulins and lower albumin secondary to estrogen production would also support the lower A:G in gravid females. In birds, elevated beta globulins are attributed to egg production [64]. In leatherbacks, alpha 2 globulins decrease over the nesting season due to inanition [65]. Our findings are important to provide context for future studies on EPH in gravid chelonians, as several of the changes associated with gravidity (elevated beta globulins, lower albumin and A:G) can also be interpreted as indicative of inflammation and poor health [17].

Male turtles had higher A:G, relative albumin, and relative alpha 2 globulins while females had higher absolute and relative beta globulin values. Many of these findings differ from those in other chelonians. Relative albumin was higher in female red-eared sliders (*Trachemys scripta elegans*) and map turtles (*Graptemys geographica*) [66], and absolute albumin was higher in female eastern box turtles [4]. Male loggerhead sea turtles had higher absolute and relative beta globulin concentrations, although there was a great deal of beta-gamma bridging indicating possible underlying disease processes in those individuals [10]. Male radiated tortoises had higher relative alpha 2 globulins during winter sampling, and female eastern box turtles and radiated tortoises had higher absolute and relative beta globulin concentrations [4][67]. Consistent with a previous study in this population of Blanding's, but contrary to several other studies in chelonians, there was no difference in total protein between the sexes [24]. Many of these findings may be confounded by the timing of our sampling, since it was concentrated during the breeding and nesting season. It is possible that if these turtles were sampled later in

the year we would find that sex-based differences in Blanding's turtles are more in line with what is reported for other species. Blanding's turtles have some unique sex-associated EPH patterns compared to other chelonians, underscoring the need for studies like this to understand species differences in clinical pathology values.

Protein fraction concentrations varied across age groups, with juvenile turtles having the highest values for A:G, relative albumin, relative alpha 1 globulins, and absolute alpha 1 globulins. Similar trends are seen in juvenile loggerheads [16], juvenile Kemp's ridley sea turtles (*Lepidochelys kempii*) [68][69], and juvenile gopher tortoises (*Gopherus polyphemus*) [70]. Adults had the highest values for relative beta globulins, relative gamma globulins, and absolute gamma globulins, which are similar to findings in other chelonians. Adult loggerheads [10], eastern box [4], and green turtles [49] all had higher absolute beta globulin levels, and adult Kemp's ridley sea turtles [69] had higher absolute beta and gamma globulins compared to their juvenile counterparts. In general, the changes found can be attributed to increased antigenic challenge as turtles age and become reproductively mature, a consistent finding in other chelonian studies [16][19][46].

The overall health status of individuals also contributed to variations in plasma protein concentrations. Higher absolute beta and gamma globulins were found in turtles with lower body fat percentage, which both increase in the presence of acute and chronic inflammation. BCS is a reliable measure of health status in other reptiles, with a good body condition score equating to better immune function and capability to fight periodic bouts of disease [71]. The elevated relative and absolute beta globulins in turtles with plastron injuries and erosions might be due to the plastron constantly being in contact with either unclean water or the ground, increasing the potential for chronic antigenic stimulation when injuries or abnormalities are present.

Appendage abnormalities were associated with higher TP and absolute beta and gamma globulins, indicating the presence of possible chronic inflammation. The loss of nails or digits from a variety of causes results in open wounds where infections may develop. Missing nails and digits might also have an impact on the turtle's overall health, making tasks like swimming and foraging more difficult [72]. In eastern box turtles, microvascular problems and primary microbial infections can cause the loss of digits and nails, indicating that even apparently minor anatomical abnormalities may have physiologically significant impacts on these turtles [73]. Integument abnormalities were associated with lower TP and absolute beta globulin concentrations. A study conducted in green and loggerhead sea turtles with traumatic wounds to their carapace, head, and/or flippers showed similar trends, with those experiencing trauma having lower beta globulin concentrations than their healthy counterparts [16].

Turtles with evidence of upper respiratory disease had lower relative prealbumin concentrations. Like albumin, prealbumin is a negative acute phase protein and decreases in the presence of inflammation [74]. Prealbumin concentrations can also be lower in cases of protein malnutrition [74]. These turtles may have lower concentrations because they are ill with upper respiratory infection, and this illness could be preventing them from taking in an adequate amount of protein. It is important to note, however, that prealbumin has not yet been validated in chelonian species, so it is unclear if prealbumin is truly what is represented in the prealbumin region of the electrophoretogram. The lower A:G in turtles with cloacal abnormalities likely consistent with inflammation associated with infection, inflammation, or stress, with a lower ratio usually indicating hyperglobulinemia [75].

The population of Blanding's turtles that was studied showed a high degree of within-individual variability in EPH parameters at different points in time. This was also identified for hematologic and plasma biochemical parameters in the same population [24]. Reptile clinical

pathology parameters are widely variable, and it is important to understand the many factors come together to influence the absolute value of each analyte. Our findings in Blanding's turtles indicate that the index of individuality and subsequent need for subject-based reference intervals should be investigated in other reptile species in order to improve the interpretation of clinical pathology testing.

There are a few limitations in this study that could be addressed in future research. Due to radiotelemetry strategy in the Lake County location, there was a bias towards adult female turtles, which are followed closely to identify nest location. Additionally, a limited number of overtly unhealthy turtles were identified, with only a few individuals having physical examination abnormalities. While this is recognized as a positive finding considering that it indicates the population is doing well, it does limit the ability to determine how EPH values change in states of poor health. Furthermore, turtles that could be identified more than once over the course of the sampling period were sampled every three to six weeks, which could indicate the fluctuation of an inflammatory response or antigenic stimulation over time.

All blood samples were obtained from the subcarapacial sinus. Blood and lymphatic vessels are very closely associated with one another at this site, making it possible that blood samples could become contaminated with lymphatic fluid during venipuncture [76]. While samples with obvious lymph contamination were discarded, undetectable lymph contamination could have negatively impacted results. Lymph contamination has been known to falsely decrease PCV and hemoglobin concentrations and may have similar affects on plasma protein concentrations [77]. For those turtles that were collect and sampled from traps, the stress of trapping could ultimately play a role in affecting plasma protein fractions, [50] but the significance has not been studied in Blanding's turtles. Timing samples to be collected pre- or post-prandial to account for lipemia is difficult to control in wildlife research, but a study

conducted in Kemp's ridley and green sea turtles revealed that feeding had very minimal effects on plasma biochemical values and are therefore unlikely to alter clinical interpretation [78].

There is a discrepancy between the use of TS verses total protein (TP). A refractometer is an efficient way to measure TS in a field research setting, but TS includes both plasma proteins as well as additional plasma solutes [79]. While there have been multiple studies conducted in other chelonian species that show a significant correlation between TS and TP [80][19], a direct relationship in Blanding's turtles has not been previously identified. Following separation, the collected plasma was frozen until protein electrophoresis could be run. Studies conducted in other reptile species have identified EPH differences in fresh plasma compared to frozen/thawed samples, and some of those studies recommended using fresh samples for best results [81][13]. In our circumstances, it would have been impractical and cost-prohibitive to ship over 200 fresh plasma samples for analysis separately, underscoring the need for us to freeze and batch-run our samples. Hemolysis also has the potential to affect EPH values, but no grossly hemolyzed samples were use in this study [82].

The baseline plasma protein reference intervals generated in this study will be useful in defining the health status of this population. There is some overlap in the top models for predicting relative and absolute EPH fractions; however there also instances where the values vary, with absolute values being high while relative values are low for the same variable. This variation demonstrates the importance of considering both relative and absolute fractions when interpreting EPH values because they might be driven by different processes. Results of this study validate that month, location, sex, age class, and health status should be considered when interpreting EPH fractions. Although EPH does not provide details on specific diseases or stressors, it is a helpful tool that can aid in identifying when intervention and treatment might be needed. With the increased use of protein electrophoresis to evaluate the health status of animals

in the veterinary medical field, the application of this tool in conservation of wild populations is becoming more widely accepted and studied. With baseline concentrations established and evaluated for variation, future studies can aim to validate and expand upon the normal reference intervals in this species.

References

1) Deem SL, Karesh WB, Weisman W. Putting theory into practice: Wildlife health in conservation. Conserv Biol. 2008;15(5): 1224-1233. doi:10.1111/j.1523-1739.2001.00336.x.

2) Ryser-Degiorgis MP. Wildlife health investigations: needs, challenges and recommendations. BMC Vet Res. 2013;9(1): 223. doi: 10.1186/1746-6148-9-223.

3) Mörner T, Obendorf DL, Artois M, Woodford MH. Surveillance monitoring of wildlife diseases. Rev Sci Tech. 2002;21(1): 67-76. doi: 10.20506/rst.21.1.1321.

4) Flower JE, Byrd J, Cray C, Allender MC. Plasma electrophoretic profiles and hemoglobin binding protein reference intervals in the eastern box turtle (*Terrapene carolina carolina*) and influences of age, sex, season, and location. J Zoo Wildl Med. 2014;45(4): 836-842. doi: 10.1638/2014-0035.1.

5) Aguirre AA, Lutz PL. Marine turtles as sentinels of ecosystem health: Is fibropapillomatosis an indicator? Ecohealth. 2004;1: 275-283. doi: 10.1007/s10393-004-0097-3.

6) Page-Karijian A, Perrault JR. Sea turtle health assessments: Maximizing turtle encounters to better understand health. In: Nahill B, editor. Sea turtle research and conservation: Lessons from Working in the Field. Elsevier, Academic Press; 2021. pp. 31-44.

7) Knotek Z, Musilova A, Pinterova K, Knotkova Z. Plasma protein electrophoresis as a diagnostic tool for endangered Asian reptiles. World Small Animal Veterinary Association World Congress Proceedings. 2015. Available from https://www.vin.com/apputil/content/defaultadv1.aspx?id=7259164&pid=14365&print=1

8) Allender MC, Junge RE, Baker-Wylie S, Hileman ET, Faust LJ, Cray C. Plasma electrophoretic profiles in the eastern massasauga (*Sistrurus catenatus*) and influences of

age, sex, year, location, and snake fungal disease. J Zoo Wildl Med. 2015;46(4): 767-773. doi: 10.1638/2015-0034.1.

9) Cooper-Bailey K, Smith SA, Zimmerman K, Lane R, Raskin RE, Denardo D. Hematology, leukocyte cytochemical analysis, plasma biochemistry, and plasma electrophoresis of wild-caught and captive-bred Gila monsters (*Heloderma suspectum*). Vet Clin Pathol. 2011;40(3): 316-323. doi: 10.1111/j.1939-165x.2011.00337.x.

10) Gicking JC, Foley AM, Harr KE, Raskin RE, Jacobson E. Plasma protein electrophoresis of the Atlantic loggerhead sea turtle, *Carretta carretta*. J Herpetol Med Surg. 2004;14(3): 13-18. doi: 10.5818/1529-9651.14.3.13.

11) Giménez M, Saco Y, Pato R, Busquets A, Martorell JM, Bassols A. Plasma protein electrophoresis of *Trachemys scripta* and *Iguana iguana*. Vet Clin Pathol. 2010;39(2): 227-235. doi: 10.1111/j.1939-165x.2009.00204.x.

12) Machado CC, Silva LFN, Ramos PRR, Takahira RK. (2006). Seasonal influence on hematologic values and hemoglobin electrophoresis in Brazilian *Boa constrictor amarali*. J Zoo Wildl Med. 2006;37(4): 487–491. doi: 10.1638/05-124.1.

13) Proverbio D, Bagnagatti De Giorgi G, Della Pepa A, Baggiani L, Spada E, Perego R, et al. Preliminary evaluation of total protein concentration and electrophoretic protein fractions in fresh and frozen serum from wild Horned Vipers (*Vipera ammodytes ammodytes*). Veterinary Clinical Pathology. 2012;41(4): 582-586. doi: 10.1111/j.1939-165x.2012.00486.x.

14) Silva LFN, Riani-Costa CCM, Ramos PRR, Takahira RK. Seasonal influence on biochemical profile and serum protein electrophoresis for *Boa constrictor amarali* in captivity. Braz J Biol. 2011;71(2): 517-520. doi: 10.1590/s1519-69842011000300023.

15) Work TM, Rameyer RA, Balazs GH, Cray C, Chang SP. Immune status of free-ranging green turtles with fibropapillomatosis from Hawaii. J Wildl Dis. 2001;37(3): 574-581. doi: 10.7589/0090-3558-37.3.574.

16) Osborne AG, Jacobson ER, Bresette MJ, Singewald DA, Scarpino RA, Bolten AB. Reference intervals and relationships between health status, carapace length, body mass, and water temperature and concentrations of plasma total protein and protein electrophoretogram fractions in Atlantic loggerhead sea turtles and green turtles. J Am Vet Med Assoc. 2010;237(5): 561-567. doi:10.2460/javma.237.5.561.

17) Cray, C. Acute phase proteins in animals. Prog Mol Biol Transl Sci. 2012;105: 113-150. doi:10.1016/b978-0-12-394596-9.00005-6.

18) Innis CJ, Tlusty M, Wunn D. Hematologic and plasma biochemical analysis of juvenile head-started northern red-bellied cooters (*Pseudemys rubriventris*). J Zoo Wildl Med. 2007;38(3): 425-432. doi:10.1638/1042-7260(2007)38[425:hapbao]2.0.co;2.

19) Rousselet E, Stacy NI, Lavictoire K, Higgins BM, Tocidlowski ME, Flanagan JP, et al. Hematology and plasma biochemistry analytes in five age groups of immature, captive-reared loggerhead sea turtles (*Caretta caretta*). J Zoo Wildl Med. 2013;44(4): 859-874. doi:10.1638/2012-0162r1.1.

20) Congdon JD, Sels RC. Relationships of reproductive traits and body size with attainment of sexual maturity and age in Blanding’s turtles (*Emydoidea blandingii*). J Evol Biol. 1993;6(4): 547-557. doi:10.1046/j.1420-9101.1993.6040547.x.

21) van Dijk PP, Rhodin AGJ. *Emydoidea blandingii*. The IUCN Red List of Threatened Species. 2011. Available from https://dx.doi.org/10.2305/IUCN.UK.2011-1.RLTS.T7709A155088836.en.

22) Glowacki G. Blanding's Turtle recovery program: 2017 Summary Report. Lake County Forest Preserve District, Blanding's Turtle Recovery Program. 2017.

23) Kuhns AR. Recovery of the Blanding's turtle (Emydoidea blandingii) at Spring Bluff Nature Preserve, Lake Count Forest Preserves. Lake County 552 Forest Preserve District, Blanding's Turtle Recovery Program Federal Aid Project T-39-D-1. 2010. Available from: https://www2.illinois.gov/dnr/conservation/iwap/documents/swgreports/t-39%20d-1%20final%20recovery%20of%20the%20blandings%20turtle.pdf

24) Mumm LE, Winter JM, Andersson KE, Glowacki GA, Adamovicz LA, Allender MC. Hematology and plasma biochemistries in the Blanding's turtle (*Emydoidea blandingii*) in Lake County, Illinois. PloS One. 2019;14(11). doi: 10.1371/journal.pone.0225130.

25) Gutzke WH, Packard GC. The influence of temperature on eggs and hatchlings of Blanding's turtles, *Emydoidea blandingii*. J Herpetol. 1987;21(2): 161. doi:10.2307/1564476.

26) Ernst CH, Lovich JE. *Emydoidea blandingii*, Blanding's Turtle. In: Turtles of the United States and Canada. 2nd ed. Baltimore: The John Hopkins University Press; 2009. pp. 233–249.

27) Mosimann JE, Bider JR. Variation, sexual dimorphism, and maturity in a Quebec population of the common snapping turtle, *Chelydra serpentina*. Can J Zool. 1960;38:19–38.

28) Newman E, Allender M, Thompson D, Glowacki G, Ivančić M, Adkesson M, et al. Measuring fat content using computed tomography to establish a body condition index in free-ranging Blanding's turtles (*Emydoidea blandingii*) in Illinois. J Zoo Wildl Med. 2019;50(3): 594-603. doi: 10.1638/2018-0154.

29) R Core Team. 2020. R: A language and environment for statistical computing. R Foundation for Statistical Computing, Vienna, Austria. https://www.R-project.org/.

30) Bates D, Maechler M, Bolker B, Walker S. Fitting linear mixed-effects models using lme4. J Stat Softw. 2015;67(1): 1-48. doi:10.18637/jss.v067.i01.

31) Kuznetsova A, Brockhoff PB, Christensen RHB. lmerTest package: Tests in linear mixed effects models. J Stat Softw. 2017; 82(13): 1-26. doi: 10.18637/jss.v082.i13.

32) Lenth RV. Least-squares means: The R package ismeans. J Stat Softw. 2016;69(1): 1-33. doi: 10.18637/jss.v069.i01.

33) Fox J, Weisberg S. An {R} Companion to Applied Regression. Sage Publications, USA. 2011. Available from http://socserv.socsci.mcmaster.ca/jfox/Books/Companion.

34) Barton K. MuMIn: Multi-Model Inference. R package version 1.43.15. 2019. Available from https://CRAN.R-project.org/package=MuMIn.

35) Lüdecke D. ggeffects: Tidy data frames of marginal effects from regression models. J Open Source Softw. 2018;3(26): 772. doi: 10.21105/joss.00772.

36) Friedrichs KR, Harr KE, Freeman KP, Szladovits B, Walton RM, Barnhart KF, et al. ASVCP reference interval guidelines: determination of de novo reference intervals in veterinary species and other related topics. Vet Clin Pathol. 2012;41: 441–453. doi:10.1111/vcp.12006.

37) Walton RM. Subject-based reference values: biological variation, individuality, and reference change values. Vet Clin Pathol. 2012;41:175–181.

38) Harris EK. Effects of intra- and interindividual variation on the appropriate use of normal ranges. Clin Chem. 1974;20:1535–1542.

39) Finnegan D. referenceIntervals: Reference Intervals. R package version 1.1.1. 2004. Available from https://CRAN.R-project.org/package=referenceIntervals.

40) Horn PS, Feng L, Li Y, Pesce AJ. Effect of outliers and nonhealthy individuals on reference interval estimation. Clin Chem. 2001;47: 2137-2145.

41) Shine R. Life-history evolution in reptiles. Annu Rev Ecol Ecol Syst. 2005;36(1): 23-46. doi:10.1146/annurev.ecolsys.36.102003.152631.

42) Huey R, Slatkin M. 1976. Costs and benefits of lizard thermoregulation. Q. Rev. Biol. 51:363–84.

43) NOAA. National Centers for Environmental Information; 2020. US Local Climatological Data. NCEI DSI 3505. Available from https://www.ncdc.noaa.gov/cdo-web/datasets/LCD/stations/WBAN:04845/detail.

44) Rozen- Rechels D, Dupoué A, Lourdais O, Chamaillé- Jammes S, Meylan S, Clobert J, et al. When water interacts with temperature: Ecological and evolutionary implications of thermo- hydroregulation in terrestrial ectotherms. Ecol Evol. 2019;9(17): 10029-10043. doi:10.1002/ece3.5440.

45) Flint M, Brand A, Bell IP, Hof CA. Monitoring the health of green turtles in northern Queensland post catastrophic events. Sci Total Environ. 2019;660: 586-592. doi:10.1016/j.scitotenv.2019.01.065.

46) Chaffin K, Norton TM, Gilardi K, Poppenga R, Jensen JB, Moler P, et al. Health assessment of free-ranging alligator snapping turtles (*Macrochelys temminckii*) in Georgia and Florida. J Wildl Dis. 2008;44(3): 670-686. doi:10.7589/0090-3558-44.3.670.

47) Deem SL, Dierenfeld ES, Sounguet GP, Alleman AR, Cray C, Poppenga RH, et al. Blood values in free-ranging nesting leatherback sea turtles (*Dermochelys coriacea*) on the coast of the Republic of Gabon. J Zoo Wildl Med. 2006;37(4): 464-471. doi:10.1638/05-102.1.

48) Deem SL, Norton TM, Mitchell M, Segars A, Alleman AR, Cray C, et al. Comparison of blood values in foraging, nesting, and stranded loggerhead turtles (*Caretta caretta*) along

the coast of Georgia, USA. J Wildl Dis. 2009;45(1): 41-56. doi:10.7589/0090-3558-45.1.41.

49) Flint M, Matthews BJ, Limpus CJ, Mills PC. Establishment of reference intervals for plasma protein electrophoresis in Indo-Pacific green sea turtles, *Chelonia mydas*. Conserv Physiol. 2015;3(1). doi: 10.1093/conphys/cov037.

50) Innis C, Merigo C, Dodge K, Tlusty M, Dodge M, Sharp B, et al. Health evaluation of leatherback turtles (*Dermochelys coriacea*) in the northwestern Atlantic during direct capture and fisheries gear disentanglement. Chelonian Conserv Biol. 2010;9(2): 205-222. doi:10.2744/ccb-0838.1.

51) Page-Karjian A, Rivera S, Torres F, Diez C, Moore D, Dam RV, et al. Baseline blood values for healthy free-ranging green sea turtles (*Chelonia mydas*) in Puerto Rico. Comp Clin Path. 2014;24(3): 567-573. doi:10.1007/s00580-014-1947-1.

52) Perrault JR, Miller DL, Eads E, Johnson C, Merrill A, Thompson LJ, et al. Maternal health status correlates with nest success of leatherback sea turtles (*Dermochelys coriacea*) from Florida. PLoS One. 2012;7(2). doi:10.1371/journal.pone.0031841.

53) Andreani G, Carpenè E, Cannavacciuolo A, Girolamo ND, Ferlizza E, Isani G. Reference values for hematology and plasma biochemistry variables, and protein electrophoresis of healthy Hermann's tortoises (*Testudo hermanni* ssp). Vet Clin Pathol. 2014;43(4): 573-583. doi:10.1111/vcp.12203.

54) Lindemann D, Allender M, Thompson D, Glowacki G, Newman E, Adamovicz L, et al. Epidemiology of Emydoidea herpesvirus 1 in free-ranging Blanding’s turtles (*Emydoidea blandingii*) from Illinois. J Zoo Wildl Med. 2019;50(3): 547-556. doi: 10.1638/2018-0074.

55) Campbell TW. Clinical pathology of reptiles. In: Mader DR, editor. Reptile Medicine and Surgery. St. Louis: Saunders Elsevier; 2006. pp 453-470.

56) Dessauer HC. (1970). Blood chemistry of reptiles: Physiological and evolutionary aspects. In: Gans C, Parsons TS, editors. Biology of the Reptilia, Vol. 3. New York: Academic Press; 1970. pp. 1-72.

57) Dowling Z, Hartwig T, Kiviat E, Keesing F. Experimental management of nesting habitat for the Blanding's turtle (*Emydoidea blandingii*). Ecological Rest. 2010;28(2): 154-159. doi:10.3368/er.28.2.154.

58) Congdon J, Kinney O, Nagle R. Spatial ecology and core-area protection of Blanding's Turtle (*Emydoidea blandingii*). Can J Zool. 2011;89: 1098-1106. doi: 10.1139/z11-091.

59) Price ER, Sotherland PR, Wallace BP, Spotila JR, Dzialowski EM. Physiological determinants of the interesting interval in sea turtles: a novel 'water-limitation' hypothesis. Biol Lett. 2019;12(20190248). doi: 10.1098/rsbl.2019.0248.

60) Yang PY, Yu PH, Wu SH, Chie CH. Seasonal hematology and plasma biochemistry references range values of the yellow-marginated box turtle (*Cuora flavomarginata*). J Zoo Wildl Med. 2014;45:278-286. doi: 10.1638/2013-0125R1.1.

61) Musilová A, Knotková Z, Pinterová K, Knotek Z. Variations of plasma protein electrophoresis in healthy captive green iguanas (*Iguana iguana*). Vet Clin Pathol. 2015;44(2): 243-248. doi: 10.1111/vcp.12238.

62) Mader DR, Divers SJ. Current therapy in reptile medicine and surgery. St. Louis: Saunders Elsevier; 2004.

63) Selcer KW, Palmer BD. Estrogen downregulation of albumin and a 170-kDa serum protein in the turtle, *Trachemys scripta*. Gen Comp Endocrinol. 1995;97(3): 340-352. doi: 10.1006/gcen.1995.1034.

64) Harris DJ. Clinical Tests. In: Tully T, Dorrestien G, Jones A, editors. Handbook of Avian Medicine, 2nd ed. Philadelphia: Elsevier; 2009. pp. 77–84.

65) Perrault JR, Wyneken J, Page-Karjian A, Merrill A, Miller DL. Seasonal trends in nesting leatherback turtle (*Dermochelys coriacea*) serum proteins further verify capital breeding hypothesis. Conserv Physiol. 2014;2(1). doi: 10.1093/conphys/cou002.

66) Leineweber C, Öfner S, Stöhr AC, Marschang RE, Mathes K. A comparison of thyroid hormone levels and plasma capillary zone electrophoresis in red- eared sliders (*Trachemys scripta elegans*) and map turtles (*Graptemys* spp.) depending on season and sex. Vet Clin Pathol. 2019;49(1): 78-90. doi:10.1111/vcp.12838.

67) Zaias J, Norton T, Fickel A, Spratt J, Altman NH, Cray C. Biochemical and hematologic values for 18 clinically healthy radiated tortoises (*Geochelone radiata*) on St Catherines Island, Georgia. Vet Clin Pathol. 2006;35(3): 321-325. doi:10.1111/j.1939-165x.2006.tb00139.x.

68) Perrault JR, Schmidt JR, Walsh CJ, Yordy JE, Tucker AD. Brevetoxin exposure, superoxide dismutase activity and plasma protein electrophoretic profiles in wild-caught Kemp's ridley sea turtles (*Lepidochelys kempii*) in southwest Florida. Harmful Algae. 2014;37: 194-202. doi:10.1016/j.hal.2014.06.007.

69) Perrault JR, Arendt MD, Schwenter JA, Byrd JL, Harms CA, Cray C, et al. Blood analytes of immature Kemp’s ridley sea turtles (*Lepidochelys kempii*) from Georgia, USA: reference intervals and body size correlations. Conserv Physiol. 2020;8(1). doi: 10.1093/conphys/coaa091.

70) Page-Karjian A, Rafferty K, Clerson X, Stacy NI, Moore JA, Hirsch SE, et al. Comprehensive health assessment and blood analyte reference intervals of gopher

tortoises (*Gopherus polyphemus*) in southeastern FL, USA. Conserv Physiol. 2021;9(1). doi: 10.1093/conphys/coab015.

71) Lamberski N, Braun J, Witte C, Christopher M, Field K, Averill-Murray R, et al. Identifying key clinical signs and validating body condition scores to minimize disease spread and maximize individual survival during desert tortoise translocations. Joint Annual Meeting for Wildlife Disease Association and European Association of Wildlife Diseases. 2012. Available at: https://www.researchgate.net/publication/271519082_Identifying_key_clinical_signs_and_validating_body_condition_scores_to_minimize_disease_spread_and_maximize_individual_survival_during_desert_tortoise_translocations.

72) Fujii JA, McLeish D, Brooks AJ, Gaskell J, Van Houtan KS. Limb-use foraging marine turtles, an evolutionary perspective. PeerJ. 2018;6. doi: 10.7717/peerj.4565.

73) Adamovicz L, Allender MC, Archer G, Rzadkowska M, Boers K, Phillips C, et al. Investigation of multiple mortality events in eastern box turtles (*Terrapene carolina carolina*). PloS One. 2018;13(4). doi: 10.1371/journal.pone.0195617.

74) Beck FK, Rosenthal TC. Prealbumin: a marker for nutritional evaluation. Am Fam Physician. 2002;65: 1575-1578.

75) Zaias J, Cray C. Protein electrophoresis: A tool for the reptilian and amphibian practitioner. J Herpetol Med Surg. 2002;12(1): 30-32.

76) Rohilla MS, Tiwari PK. Simple method of blood sampling from Indian freshwater turtles for genetic studies. Acta Herpetol. 2020;3(4): 65-69.

77) Stacy NI, Alleman AR, Sayler KA. Diagnostic hematology of reptiles. Clin Lab Med. 2011;31(1): 87-108. doi: 10.1016/j.cll.2010.10.006.

78) Anderson ET, Minter LJ, Clarke EO, Mroch RM, Beasley JF, Harms CA. The effects of feeding on hematological and plasma biochemical profiles in green (*Chelonia mydas*) and Kemp's ridley (*Lepidochelys kempii*) sea turtles. Vet Med Int. 2011;2011. doi: 10.4061/2011/890829.

79) Stewart K, Mitchell MA, Norton T, Krececk RC. Measuring the level of agreement in hematologic and biochemical values between blood sampling sites in leatherback sea turtles (*Dermochelys coriacea*). J Zoo Wildl Med. 2021;43(4): 719-725. doi: 10.1638/2011-0045R.1.

80) Bolten AB, Bjorndal KA. Blood profiles for a wild population of green turtles (*Chelonia mydas*) in the southern Bahamas: Size-specific and sex-specific relationships. J Wildl Dis. 1992;28(3): 407-413. doi: 10.7589/0090-3558-28.3.407.

81) Alberghina D, Marafioti S, Spadola F, Panzera M, Piccione G. Influence of short-term storage conditions on the stability of total protein concentrations and electrophoretic fractions in plasma samples from loggerhead sea turtles, *Caretta caretta*. Comp Clin Path. 2014;24(5): 1091-1095. doi:10.1007/s00580-014-2038-z.

82) Stacy NI, Chabot RM, Innis CJ, Cray C, Fraser KM, Rigano KS, et al. Plasma chemistry in nesting leatherback sea turtles (*Dermochelys coriacea*) from Florida: Understanding the importance of sample hemolysis effects on blood analytes. PloS One. 2019;14(9). doi:10.1371/journal.pone.0222426.

Supplementary File 1. Combined Data.

All project data collected upon turtle capture, which includes identifying information, temporal data, capture method, weather data, measurements, physical exam findings, and release information.

Fig 1

Click here to access/download;Figure;Fig1.tif

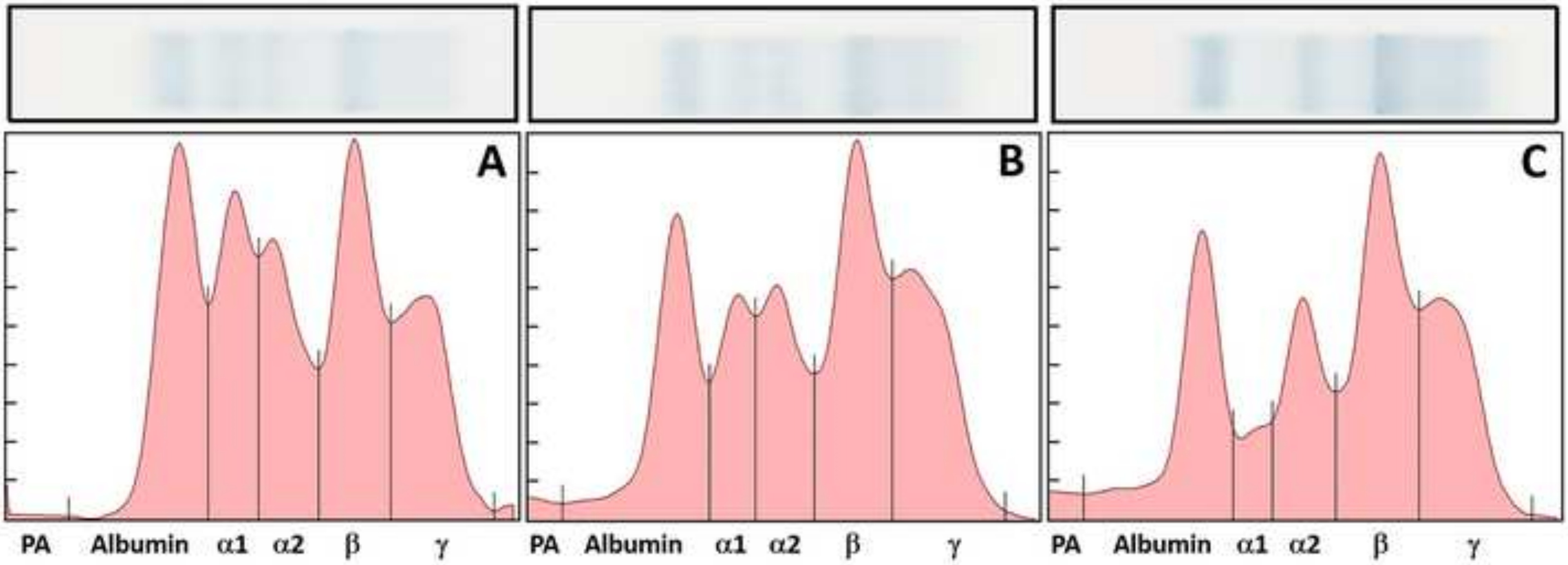

Fig 2

Click here to access/download;Figure;Fig2.tif

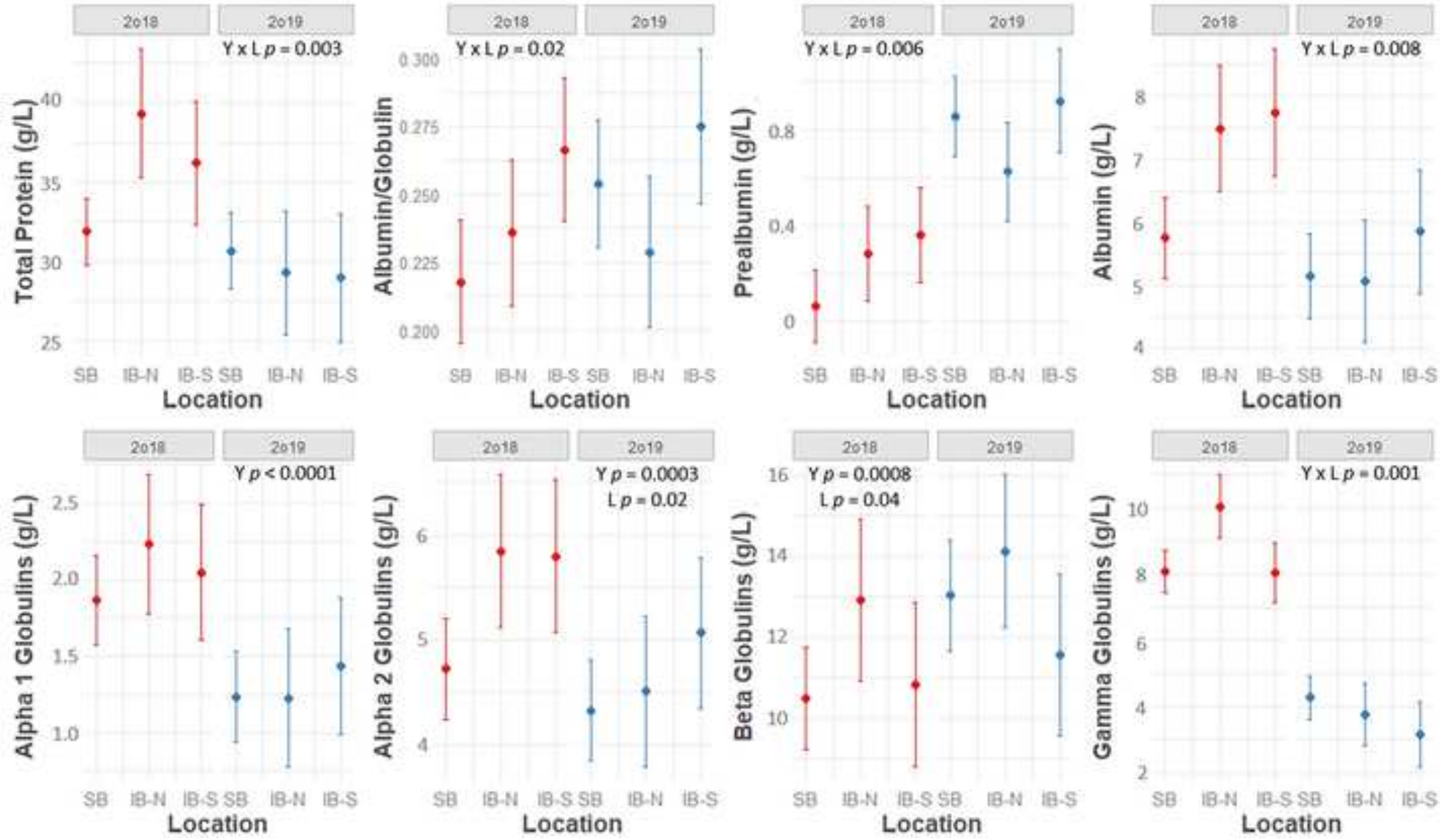

Fig 3

Click here to access/download;Figure;Fig3.tif

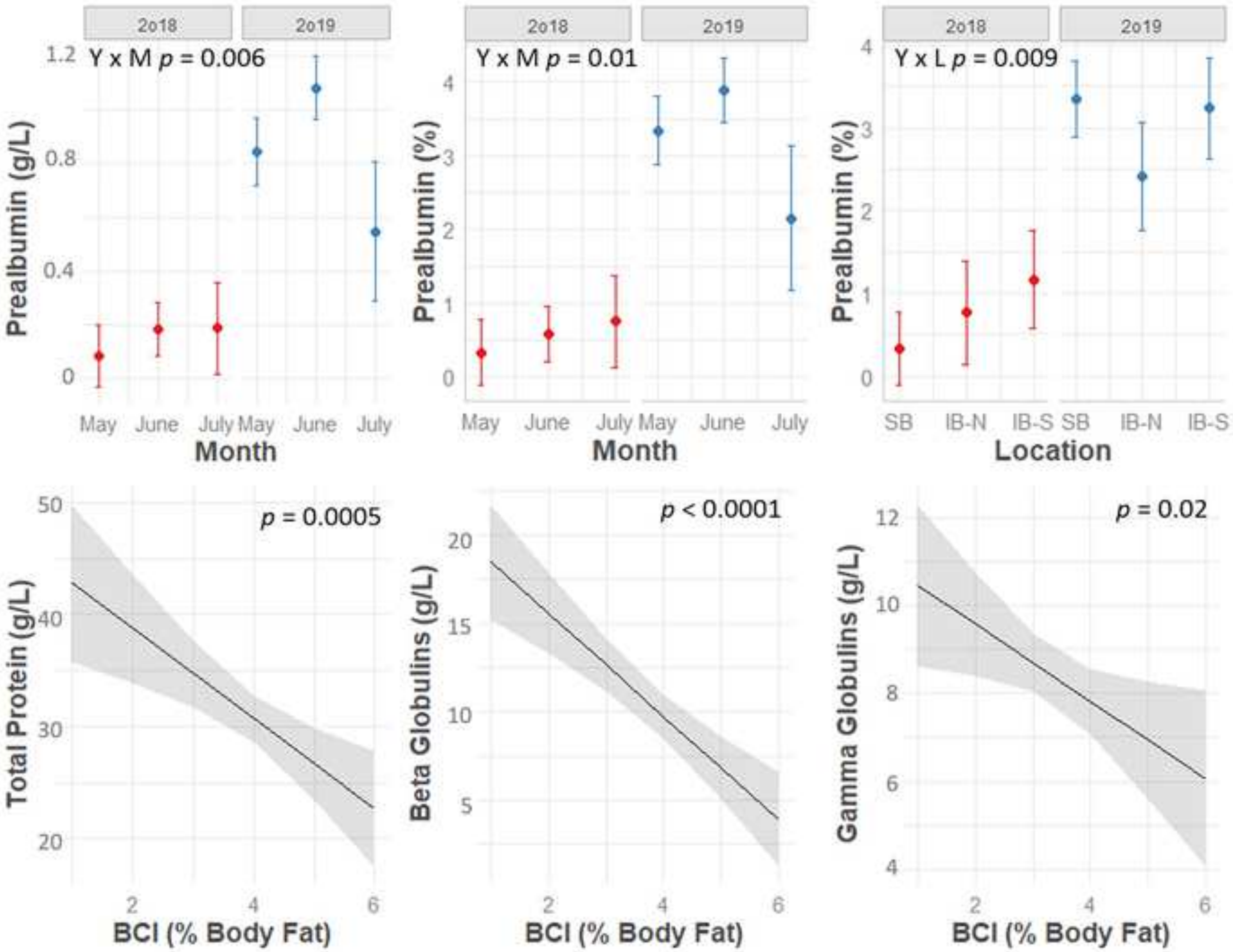

Fig 4

Click here to access/download;Figure;Fig4.tif

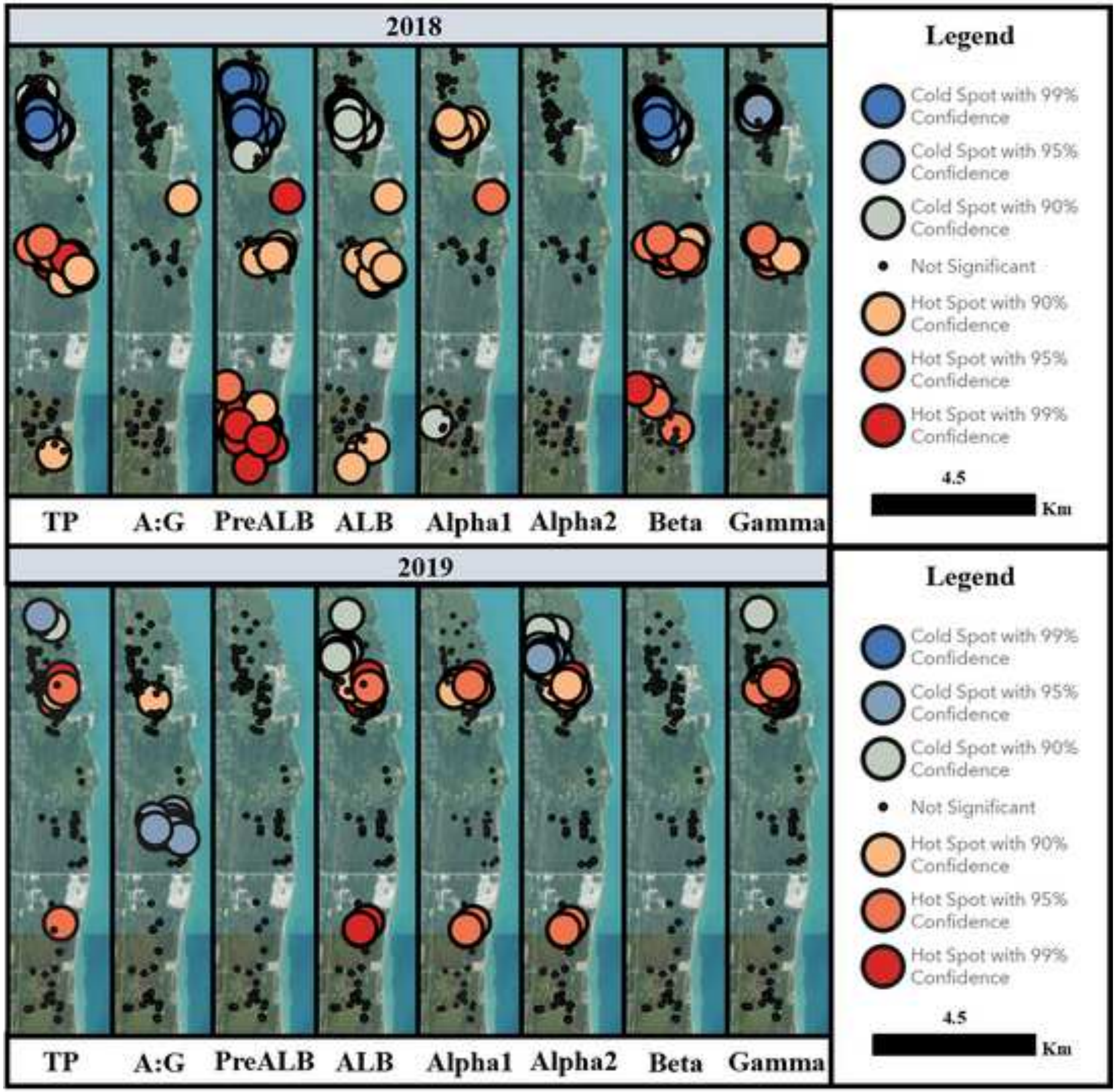

Click here to access/download
Supporting Information
SuppFile1.xlsx

Title: Plasma electrophoresis profiles of Blanding's turtles (*Emydoidea blandingii*) and influences of month, age, sex, health status, and location

Authors:

Kirsten E. Andersson, DVM~~BS~~,[1]* Laura Adamovicz, DVM, PhD,[1,2] Lauren E. Mumm, DVM,[1] Samantha E. Bradley,[1] John M. Winter, DVM,[1] Gary Glowacki, MS,[3] Carolyn Cray, PhD[4], Matthew C. Allender, DVM, MS, PhD, Dipl. ACZM[1]

Affiliations:

[1] Wildlife Epidemiology Laboratory, College of Veterinary Medicine, University of Illinois, Urbana, IL 61802 USA

[2] Veterinary Diagnostic Laboratory, College of Veterinary Medicine, University of Illinois, Urbana, IL 61802 USA

[3] Lake County Forest Preserve District, Libertyville, IL 60048 USA

[4] Department of Pathology & Laboratory Medicine, School of Medicine, University of Miami, Miami, FL 33136 USA

Corresponding Authors:

Kirsten E. Andersson, kkeandersson@gmail.com

Matthew C. Allender, mcallend@illinois.edu

Abstract:

B~~Establishing b~~aseline plasma electrophoresis profiles (EPH) are important components of overall health and may aid in the conservation and captive management of species. The aim of this study was to establish ~~baseline values for~~ plasma protein fractions for free-ranging Blanding’s turtles (*Emydoidea blandingii*) and evaluate differences due to age class (adult vs. sub-adult vs. juvenile), sex (male, female, or unknown), year (2018 vs. 2019), month (May vs. June vs. July), health status, ~~s (multiple health factors considered)~~ and geographical location (managed vs. unmanaged sites). Blood samples were obtained from 156 Blanding’s turtles in the summer of 2018 and 129 in 2019 at two adjacent sites in Illinois. Results of the multivariate analysis demonstrated that age class, sex, year, month, health status, and geographical location all contributed to the variation observed in free-ranging populations. Adult females had the highest concentration of many protein fractions, likely associated with reproductive activity. Juveniles had lower protein concentrations. Temperature ~~swings~~ and rainfall differences between years impacted concentrations between 2018 and 2019, while May and June of both years saw higher levels in some protein fractions likely due to peak breeding and nesting season. Individuals with evidence of trauma or disease also showed increased plasma protein ~~concentrations~~ fractions when compared to those that were considered healthy. ~~Between locations~~The two sites ~~, there was~~showed a wide/large variation ~~between~~ over the two years ~~at the two sites~~. All of these factors emphasize the importance of considering multiple demographic or environmental factors when interpreting the EPH fractions. Establishing ~~baseline concentration~~ ranges for these analytes will ~~aid in~~allow ~~further health studies~~ investigation into~~ating~~ disease prevalence and other environmental factors impacting this endangered species.

Key Words:

~~Blanding's turtle~~Chelonians, plasma proteins, conservation, protein electrophoresis, endangered species, health monitoring, wildlife, immune response

Introduction

Climate change, habitat destruction, and disease are ~~negatively~~potentially affecting the ~~impacting~~ sustainability and conservation of species [1]. Wildlife health surveillance has become a critical component to maintain imperiled populations [1][2**]**, but determining health is complex and involves several modalities, including physical examination, clinical pathology, pathogen presence, and contaminant exposure [3][4][5][6]. ~~.~~Protein electrophoresis (EPH), a component of clinical pathology investigation, has become commonly used in wildlife studies [7~~5~~]. Reference interval data in several reptilian species exist and have ~~s~~ proven to be valuable in understanding how they handle stress and disease [4][7~~5~~][8~~6~~][9~~7~~][10~~8~~][11~~9~~][12~~0~~][13~~1~~][14~~2~~][15~~3~~][16]~~.~~

Formatted: Font: Bold

Plasma ~~and acute phase proteins (APPs)~~ proteins are key players in the body's innate immune response, and fluctuations in concentrations serve to indicate the presence of inflammation, infection, neoplasia, stress, or trauma [17~~4~~]. In head-started red-bellied cooters (*Pseudemys rubriventris*) and captive-reared loggerhead sea turtles (*Caretta caretta*), variations in protein fractions were associated with differences in age, diets, immune stimulation, ~~or~~ and reproductive stage [18~~5~~][19~~6~~], indicating baseline differences exist and responses to changes in demographic and environmental variation can be measured.

The Blanding's turtle is a semi-aquatic, long-lived species of turtle experiencing population declines over much of its range in southern and central ~~parts of~~ Canada and northern ~~parts of the~~ United States. Individual turtles can live up to 80 years of age in the wild [20~~17~~], but urban development, road mortality events, climate change, illegal poaching, and disease remain the most common threats to sustainability [21~~18~~]. The Chiwaukee Prairie – Illinois Beach Lake

Plain (Lake Plain) contains a population of Blanding's turtles, in which active conservation efforts are aimed at improving the long-term viability in northeastern Illinois and southeastern Wisconsin [22~~19~~]. In the summer of 2015, conservation efforts incorporated physical exam and health assessment data to aid in a greater understanding of the biological threats these animals face.

The objective of this study was to establish baseline plasma protein fractions for free-ranging Blanding's turtles and to determine differences between age classes (adult vs. sub-adult vs. juvenile), sex~~es~~ (male vs. female), years (2018 vs. 2019), months (May vs. June vs. July), health status, and geographical location. It was hypothesized that total protein levels would be higher in adults than subadults and juveniles, higher in females than males, higher in May than in June and July, and higher in unhealthy turtles than healthy turtles.

Materials and Methods

Study sites

Blanding's turtles were sampled from three sites within the Lake Plain including Spring Bluff-Chiwaukee Prairie (SBCP), the managed site, and the North and South units of Illinois Beach State Park (IBSP), the unmanaged sites. SBCP consists of approximately 535 acres of high-quality coastal dune and swale habitat along the coast of Lake Michigan in Illinois and Wisconsin, whereas IBSP consist of 4,160 acres of dune, prairie, oak-savannah, and wetland habitats along 6.5 miles of coastline [22~~19~~]. Management of mesopredators~~ and invasive plants species also differs between the sites~~is also more robust in SBCP than IBSP, with studies focusing on camera trap surveillance for predator presence throughout the year as well as nest predation rates [23].

Capture Methods

Turtles were captured with the aid of radiotelemetry, hoop net trap, or incidentally by hand. Radiotelemetry is a three-part system using a radio transmitter, a radio antenna, and a radio receiver. The transmitter is attached to the turtle's shell, which transmits a signal to the antenna and correlates to a beeping produced by the receiver that gets louder as the animal gets closer [23][24]. Hoop traps were placed in marsh waters and areas that were characteristic of Blanding's habitat or locations near previous Blanding's turtle capture sites. These traps were checked every 24 hours and remained in the same location for up to five days. Telemetry, traps, and incidental captures were used in both field sites. Many turtles, especially those equipped with a radio transmitter, were sampled up to two times per summer.

Physical examination and Sample collection

Each turtle was assigned a permanent ID, marking the shell with a notch code unless previously marked as well as inserting a pit tag (microchip) under the skin, and mass, sex, and age class ~~was~~ were recorded. Sex was classified as male, female, or unknown. Sex of head-started turtles, which are clutches deposited in captivity from free-range females, was known due to established incubation temperatures, as males were incubated at 26.5°C and females at 31.0°C [23][25~~0~~]. The sex of adults was determined based on plastron concavity [26~~1~~]. The sex of most sub-adults and juveniles was estimated based on position of cloacal opening [27~~2~~]. Wild-born individuals were classified as unknown sex when a confident determination could not be made. Age class was characterized as juvenile (<250 grams), sub-adult (250-750 grams), or adult (>750 grams). Blanding's turtles were deemed sexually mature at 750 grams and over by the Lake County Forest Preserve District (LCFPD) based on the lightest fertile female noted. This methodology is based on a previously published study by Mumm, etl al [24]. Body fat

percentage (FP) was calculated using a published calculation from the relationship of carapace length and mass [28~~3~~]. Physical examinations were performed noting visual appearance of the eyes, nose, oral cavity, ears, legs, digits, shell, integument, and cloaca. Gravidity was assessed using digital palpation of the prefemoral fossa. For the purpose of statistical analysis, females were classified as gravid if they had palpable eggs or if they had nested within one week of sampling. Nesting was determined either by observation of nesting behavior or the lack of palpable eggs after having previously been confirmed gravid. Turtles were classified as either “apparently healthy” or “unhealthy” based on the presence of clinically significant physical exam abnormalities, including open fractures or wounds; ocular, oral or nasal discharge; depressed mentation; missing nails, digits, or appendages; and evidence carapace/plastron damage.

Whole blood was collected from the sub-carapacial sinus via 22-gauge or 25-gauge needle, subject to the size of the individual. No more than 0.6% of body weight of whole blood was drawn and placed into lithium-heparinized plasma separator tubes. Blood samples were placed on ice in a cooler for one to five hours depending on time of collection until returning to the lab each afternoon. Total protein (TP) was estimated using refractometry. Whole blood samples were centrifuged at 4,185 g for 10 minutes, stored at -20°C for one to four months, and shipped on dry ice to the University of Miami at the end of the field season. All individuals ~~were marked with a shell notch code unless previously marked, assigned a pit tag and~~ were released at coordinates of capture. All animal sampling was permitted by the following organizations: Department of Natural Resources (IDNR) (Scientific Collectors Permits (SCP): NH17.5065, NH18.5065, and IDNR Endangered and Threatened permits: SBT-16-062, 1199, 14-046, and 1042), the Wisconsin Department of Natural Resources (WIDNR) (SCP: SCP-SOD-004-2013 and WIDNR Scientific Research License: SRLN-18-026), and the University of Illinois Institutional Animal Care and Use Committee (Protocols: 18000 and 18165).

Protein Electrophoresis

Plasma samples were analyzed according to the procedure provided by the Helena SPIFE 3000 system with the use of Split Beta gels (Helena Laboratories, Inc., Beaumont, Texas 77707, USA). Results were produced after gel scanning and analysis by Helena software. Fraction delimits were placed as previously demonstrated for other reptiles [10~~8~~]. Plasma protein fractions were divided into the following six fractions: a fraction migrating in the prealbumin region ("prealbumin"), albumin, alpha 1 globulins, alpha 2 globulins, beta globulins, and gamma globulins (Fig 1). Percentages for each fraction were determined by this software, which gave the relative value, and absolute values (g/~~d~~L~~l~~) for each fraction were obtained by multiplying the percentage by the ~~total protein (~~TP~~)~~ concentration. The albumin:globulin ratio (~~AGR~~A:G) was calculated by dividing the sum of albumin and prealbumin by the sum of the globulin fractions.

Fig 1. Electrophoretogram comparison of plasma protein fractions.

A visual representation of the plasma protein fraction distribution amongst three representatives from each age classes. The top panel shows the protein as they appeared on the gel following electrophoresis, and the bottom panel shows a graphic representation of the concentration differences. Juvenile (A), sub-adult (B), and adult(C).

Statistical analysis

All statistical analyses were conducted in R version 3.6.3 [29~~4~~] at an alpha level of 0.05, unless otherwise specified. Data distributions were assessed using histograms and the Shapiro-Wilk test and transformation was pursued, if necessary, to meet modeling assumptions.

Formatted: Font: 10 pt, Font color: Auto

Formatted: Font: Bold

Formatted: Font: Not Bold

Formatted: Font: Not Italic

Protein electrophoresis fractions were modeled using general linear mixed models with the lme4 and lmerTest packages [30~~25~~][31~~26~~]. Fixed effects included spatiotemporal variables (year, month, location), demographic variables (sex, age class, gravidity), and health variables (body condition, presence/absence of physical exam abnormalities), while turtle ID was included as a random effect. Post-hoc testing was performed using the lsmeans package with a Tukey correction to control for multiple statistical tests [32~~27~~]. Fixed effects with univariable p-values < 0.15 were considered in multivariable models testing specific biological hypotheses about the effects of spatiotemporal, demographic, and health variables on EPH values. Variance inflation factors were evaluated for multivariable models to identify and exclude highly collinear (VIF > 10) predictor variables (function vif, package car) [33~~28~~]. Candidate model sets were constructed for each EPH fraction and ranked using an information-theoretic approach [34~~29~~]. Figures were constructed using the ggeffects package [35~~30~~]. Spatial clustering of EPH fractions was modeled in ArcGIS version 10.6 using hot spot analysis with the Gettis-Ord Gi* statistic from the spatial statistics toolbox. Hot spots are areas with higher plasma protein concentrations, while cold spots are areas with lower plasma protein concentrations.

Formatted: Font: 12 pt

Coefficients of variation were determined for each EPH fraction using data from apparently healthy turtles evaluated at multiple time points (CV_I) and only a single time point (CV_G). The index of individuality (II) was calculated as CV_I / CV_G and was used to infer the need for subject-based vs. population-based reference intervals [36~~1~~][37~~2~~]. When the II is < 0.6, subject-based reference intervals was used, while an II > 1.4 population-based reference intervals were created (Harris, 1974). When the II was between 0.6 and 1.4, population-based reference intervals were used [37~~2~~][38~~3~~]. Reference change values (RCV) were calculated for each EPH analyte using a published formula [36~~1~~][37~~2~~].

Population-based reference intervals were also determined for each analyte using the nonparametric method (referenceIntervals package) [39~~4~~], according to American Society for Veterinary Clinical Pathology guidelines [36~~1~~]. Outliers were identified and excluded using Horn's method [40~~35~~]. Ninety percent confidence intervals were generated around the limits of each reference interval using nonparametric bootstrapping with 5000 replicates. The population-based reference interval dataset included only turtles sampled once, and a randomly-selected single time point (https://www.random.org/) from serially-sampled individuals.

Results

Two hundred and eighty-five samples were collected from 215 individual turtles. Fifty animals were sampled two times approximately one year apart, and ten animals were sampled three times – twice within the 2018 active season and ~~the~~ a third time in 2019. One hundred fifty-six samples were obtained in 2018, while 129 were collected in 2019. One hundred seventy-eight samples were collected at SBCP, 51 were from IBSP North Unit, 54 were from IBSP South Unit, and two individuals did not have location data recorded. Samples were collected in May (N = 129), June (N = 120), and July (N = 36) from 171 adults, 83 sub-adults, and 31 juveniles. Sex distribution included 174 females, 75 males, and 36 turtles of unknown sex. Twenty-two females were gravid.

Blood samples were collected from the subcarapacial sinus due to its relative ease of access and minimal restraint requirement in a field setting. Grossly hemolyzed or lymph contaminated samples were removed from the study, although microscopic hemolysis cannot be ruled out. The timing of sampling relative to food consumption is unknown, so post-prandial changes, such as lipemia, could impact results. Physical examination was largely unremarkable for most individuals. Clinical signs of upper respiratory disease (URD), including oculonasal

discharge, blepharoedema, and/or oral plaques were present in eleven turtles. Integumentary abnormalities including abrasions, lacerations, and/or nodules were present in 28 turtles. Appendicular abnormalities including abnormal nails and/or missing digits, feet, limbs, or tail tips were present in 42 animals. Cloacal abnormalities consisting of erythema, swelling, and/or discharge were present in 15 animals. Shell abnormalities involved the carapace (erosions – 25, predator injury - 8) and plastron (erosions – 99, predator injury - 12). In total, 30 turtles had active physical exam abnormalities significant enough to compromise health, and these individuals were excluded from the reference interval dataset.

All absolute EPH parameters varied by year, with TP (effect size = ~~0.~~3.70g/~~d~~L, 95% CI = ~~0.~~1.60 – ~~0.~~5.90g/~~d~~L, p = 0.01~~009~~), albumin (effect size = ~~0.~~1.20g/~~d~~L, 95% CI = 0.~~0~~70 – ~~0.~~1.70g/~~d~~L, p < 0.0~~00~~1), alpha 1 globulins (effect size = 0.~~0~~86g/~~d~~L, 95% CI = 0.~~0~~60 – ~~0.~~1.10g/~~d~~L, p < 0.~~00~~01) alpha 2 globulins (effect size = 0.~~0~~63g/~~d~~L, 95% CI = 0.~~0~~26 – ~~0.~~1.00g/~~d~~L, p = 0.01~~003~~), and gamma globulins (effect size = ~~0.~~4.30g/~~d~~L, 95% CI = ~~0.~~3.70 – ~~0.~~4.80g/~~d~~L, p < 0.~~00~~01) higher in 2018 than 2019, and ~~AGR~~ A:G (effect size = 0.02~~16~~, 95% CI = 0.01~~03~~ – 0.03, p = 0.02), prealbumin (effect size = 0.~~0~~67g/~~d~~L, 95% CI = 0.~~0~~58 – 0.~~0~~77g/~~d~~L, p < 0.~~00~~01), and beta globulins (effect size = ~~0.~~2.60g/L, 95% CI = ~~0.~~1.60 – ~~0.~~3.60g/L, p = 0.0~~00~~1~~8~~) higher in 2019 than 2018. The relationships between relative EPH fraction and year were similar, except there was no significant association between relative alpha 2 globulins and year (p = 0.34).

The effects of location on absolute EPH parameters depended on year (significant Year*Location interaction, p < 0.05) for TP, ~~AGR~~A:G, prealbumin, albumin, and gamma globulins (Fig 2~~1~~). A significant year*location effect was also identified for relative prealbumin (Fig 3~~2~~). Location influenced absolute alpha 2 globulins (p = 0.02), absolute beta globulins (p = 0.04), and relative gamma globulins (p = 0.01~~003~~) independent of year, while it was not a

statistically significant predictor of absolute alpha 1 globulins, relative albumin, relative alpha 1 globulins, relative alpha 2 globulins, or relative beta globulins ($p > 0.05$). In addition to the site-level effects identified using general linear models, finer-scale clusters of high and low absolute EPH values were identified using spatial modeling (Figs 4~~3 & 4~~). The location of these spatial clusters varied between years, and differences occurred both between and within study sites, especially in turtles sampled at SBCP in 2019 (Fig 4~~4~~).

Fig 2~~1~~. Model predictions for plasma protein electrophoresis values based on year and location.

Model predictions with 95% confidence intervals for plasma protein electrophoresis values in free-living Blanding's turtles (*Emydoidea blandingii*) based on year and location. Model estimates were produced by top-ranking general linear mixed models (see Table 2). SB = Spring Bluff-Chiwaukee Prairie, IB-N = Illinois Beach North, IB-S = Illinois Beach South.

Fig 3~~2~~. Model predictions for plasma protein electrophoresis values based on year, location, month, and BCI.

Model predictions with 95% confidence intervals for plasma protein electrophoresis values in free-living Blanding's turtles (*Emydoidea blandingii*) based on year, location, month, and body condition index (BCI). Model estimates were produced by top-ranking general linear mixed models (see Table 2).

Fig 4~~3~~. 2018 and 2019 spatial clusters of plasma protein electrophoresis values in free-living Blanding's turtles (*Emydoidea blandingii*).

Study site map displaying hot and cold spots for each analyte in 2018 and 2019. Hot spots are areas with higher plasma protein concentrations, while cold spots are areas with lower plasma

protein concentrations. SBCP, the managed site, is the northernmost territory on the map followed by the North unit of IBSP as the central territory and the South unit of IBSP as the southernmost territory. Evaluated using Gettis-Ord Gi* models. Map obtained from USGS National Map Viewer. TP = total protein (g/~~d~~L), PreALB = prealbumin (g/~~d~~L), ALB = albumin (g/~~d~~L), Alpha1 = alpha 1 globulins (g/~~d~~L), Alpha2 = alpha 2 globulins (g/~~d~~L), Beta = beta globulins (g/~~d~~L), Gamma = gamma globulins (g/~~d~~L), A:~~/~~G = albumin / globulin.

~~**Fig 4. 2019 spatial clusters of plasma protein electrophoresis values in free-living Blanding's turtles (*Emydoidea blandingii*).**~~

~~Evaluated using Gettis-Ord Gi* models. TP = total protein (g/dL), PreALB = prealbumin (g/dL), ALB = albumin (g/dL), Alpha1 = alpha 1 globulins (g/dL), Alpha2 = alpha 2 globulins (g/dL), Beta = beta globulins (g/dL), Gamma = gamma globulins (g/dL), A/G = albumin / globulin.~~

Formatted: Indent: First line: 0"

~~AGR~~ A:G ($p < 0.0$~~00~~1), absolute albumin ($p = 0.0$~~00~~1), relative albumin ($p < 0.0$~~00~~1), and relative gamma globulins ($p = 0.02$) differed by month, while the effects of month on absolute and relative prealbumin concentration depended on year (significant Month*Year interaction) (Fig 2, Tables 1 and 2). Specifically, relative gamma globulins were significantly higher in May compared to June while ~~AGR~~A:G, absolute albumin, and relative albumin were significantly higher in June compared to both May and July. Relative and absolute prealbumin followed a similar trend to ~~AGR~~ A:G and albumin in 2019, but values were not significantly different by month in 2018.

Table 1. Protein electrophoresis values that vary by month and the presence of plastron abnormalities in free-living Blanding's turtles (*Emydoidea blandingii*). Model estimates were produced by top-ranking general linear mixed models (see Table 2).

Analyte	Level	Model Estimate	SE	Contrast	Difference	95% CI	P - value
**Month**							
**Albumin/Globulin**	May	0.271	0.01	May vs. June	-0.04	-0.06, -0.02	< 0.0001
	June	0.31	0.01	June vs. July	0.04	0.01, 0.06	0.0102
	July	0.272	0.012	May vs. July	-0.0102	-0.03, 0.02	1.00
**Albumin (g/dL)**	May	6.000.6	0.040	May vs. June	-1.0.140	-2.0.220, -0.060	0.001
	June	7.0.740	0.040	June vs. July	1.30.13	0.020, 2.0.230	0.01
	July	6.0.610	0.050	May vs. July	-0.010	-1.0.110, 0.090	1.00
**Albumin (%)**	May	19.00	0.64	May vs. June	-2.10	-3.30, -0.95	0.0001
	June	21.10	0.44	June vs. July	1.80	0.33, 3.20	0.01
	July	19.40	0.68	May vs. July	-0.36	-1.70, 0.98	0.80
**Gamma Globulins (%)**	May	17.20	0.41	May vs. June	1.31	0.20, 2.40	0.02
	June	15.90	0.39	June vs. July	-0.81	-2.30, 0.70	0.40
	July	16.70	0.58	May vs. July	0.51	-0.97, 2.00	0.70
**Plastron**							
**Beta Globulins (g/dL)**	Normal	11..130	0.070	N vs. E	-1.60.106	-2.0.290, -0.040	0.01
	Erosions	1.2.90	0.070	E vs. I	-2.10.021	-4.0.490, 0.070	0.15
	Injury	15.00.5	1.0.140	N vs. I	-3.0.370	-6.0.660, -0.090	0.01
**Beta Globulins (%)**	Normal	35.60	0.80	N vs. E	-0.27	-1.64, 1.10	0.70
	Erosions	35.90	0.90	E vs. I	-3.33	-6.40, -0.28	0.03

Formatted: Font: Bold

	Injury	39.20	1.50	N vs. I	-3.60	-6.60, -0.61	0.02

SE = standard error. N = normal. E = erosions. I = injury.

Table 2. Top models for predicting protein electrophoresis values in free-living Blanding's turtles (*Emydoidea blandingii*) based on Akaike's information criterion, corrected for sample size (AIC_c).

Analyte	Model	N	K	AIC_c	w_i
**Total Protein (g/~~d~~L)**	Y * L + BCI + Integ + App	265	11	748.00	0.98~~83~~
**Albumin / Globulin**	Y * L + M + Sex + Age + Cloaca	279	15	-825.40	0.98~~1~~
**Prealbumin (g/~~d~~L)**	Y * L + Y * M + URD	279	13	-995.90	0.80~~798~~
**Prealbumin (%)**	Y * L + Y * M + URD	279	13	-1534.60	0.69~~89~~
**Albumin (g/~~d~~L)**	Y * L + M + App + Gravid	278	12	-7.70	0.57~~66~~
**Albumin (%)**	Y + M + Age + Sex + Gravid	279	11	-1154.10	0.94~~38~~
**Alpha 1 Globulins (g/~~d~~L)**	Y + Age + Carapace	279	10	-423.10	0.58~~79~~
**Alpha 1 Globulins (%)**	Y + Age + Carapace	279	10	-1232.30	0.80~~798~~
**Alpha 2 Globulins (g/~~d~~L)**	Y + L + Age	283	8	-157.30	0.76~~58~~
**Alpha 2 Globulins (%)**	Sex + Age + Gravid	280	8	-1207.10	0.95~~47~~
**Beta Globulins (g/~~d~~L)**	Y + L + Sex + BCI + Integ + Plastron + App	265	15	307.80	0.99~~5~~
**Beta Globulins (%)**	Y + Age + Sex + Plastron + Gravid	280	13	-891.00	0.99~~86~~
**Gamma Globulins (g/~~d~~L)**	Y * L + Age + BCI + App	266	12	-34.80	0.99~~3~~
**Gamma Globulins (%)**	Y + L + M + Age	283	10	-1129.80	0.86~~55~~

Y = year, L = location, M = month, BCI = body condition index, Integ = integument, App = appendages, URD = upper respiratory disease.

Formatted: Font: Bold

Formatted Table

Age class influenced ~~AGR~~ A:G and all relative EPH fractions except prealbumin; it was also found to be an important predictor of absolute alpha 1 globulins, alpha 2 globulins, and gamma globulins (Table 3). Juvenile turtles had the highest values for ~~AGR~~A:G, relative albumin, relative alpha 1 globulins, and absolute alpha 1 globulins. Subadults had the highest values for relative and absolute alpha 2 globulins. Adults had the highest values for relative beta globulins, relative gamma globulins, and absolute gamma globulins.

Table 3. Protein electrophoresis values that vary by age class in free-living Blanding's turtles (*Emydoidea blandingii*). Model estimates were produced by top-ranking general linear mixed models (see Table 2). SE = standard error. Ad = adult. SA = sub-adult. Juv = juvenile.

Formatted: Font: Bold

Analyte	Level	Model Estimate	SE	Contrast	Difference	95% CI	P - value
**Albumin/Globulin**	Adult	0.2548	0.0018	Ad vs. Juv	-0.00773	-0.0.11108, -0.00438	< 0.00001
	Sub-Adult	0.283	0.0011	Ad vs. SA	-0.00435	-0.00657, -0.00114	0.01004
	Juvenile	0.321	0.00216	SA vs. J	-0.00438	-0.00774, -0.00102	0.03
**Albumin (%)**	Adult	17.90	0.41	Ad vs. Juv	-3.80	-5.80, -1.80	<0.0001
	Sub-Adult	19.80	0.63	Ad vs. SA	-1.80	-3.10, -0.56	0.0102
	Juvenile	21.80	0.87	SA vs. J	-2.00	-4.00, 0.02	0.05
**Alpha 1 Globulins (g/dL)**	Adult	1.600.16	0.010	Ad vs. Juv	-20.240	-2.0.290, -1.0.180	< 0.0001
	Sub-Adult	2.700.27	0.010	Ad vs. SA	-1.000.1	-0.141.40, -0.070	< 0.0001
	Juvenile	4.000.4	0.020	SA vs. J	-1.0.130	-1.0.190, -0.080	< 0.0001
**Alpha 1 Globulins (%)**	Adult	5.20	0.30	Ad vs. Juv	-9.30	-10.60, -8.00	< 0.0001
	Sub-Adult	8.70	0.40	Ad vs. SA	-3.50	-4.40, -2.60	< 0.0001
	Juvenile	14.50	0.60	SA vs. J	-5.80	-7.20, -4.40	< 0.0001
**Alpha 2 Globulins (g/dL)**	Adult	5.100.51	0.020	Ad vs. Juv	0.010	-0.070, 0.090	1.00
	Sub-Adult	5.800.58	0.030	Ad vs. SA	-0.070	-1.0.130, -0.020	0.01
	Juvenile	5.000.5	0.040	SA vs. J	0.080	0.004, 1.0.170	0.04
**Alpha 2 Globulins (%)**	Adult	14.30	0.37	Ad vs. Juv	-1.10	-2.90, 0.60	0.30
	Sub-Adult	16.40	0.50	Ad vs. SA	-2.10	-3.20, -1.00	< 0.0001

	Juvenile	15.50	0.78	SA vs. J	1.00	-0.90, 2.80	0.40
**Beta Globulins (%)**	Adult	41.70	0.79	Ad vs. Juv	9.61	6.40, ~~-~~-~~-~~12.83	< 0.0~~00~~1
	Sub-Adult	34.40	0.97	Ad vs. SA	7.28	5.40, –~~-~~9.16	< 0.0~~00~~1
	Juvenile	32.10	1.50	SA vs. J	2.33	-0.98, 5.65	0.20
**Gamma Globulins (g/~~d~~L)**	Adult	~~0.7~~7.00	0.~~0~~30	Ad vs. Juv	2.00~~0.2~~	0.~~0~~60, 3.~~0.3~~40	0.01~~03~~
	Sub-Adult	~~0.66~~6.60	0.~~0~~40	Ad vs. SA	0.~~0~~30	-0.~~0~~80, 1.~~0.1~~40	0.85
	Juvenile	~~0.5~~5.00	0.~~0~~50	SA vs. J	1.~~0.1~~70	0.~~0~~60 ~~-~~– 3.00~~0.3~~	0.01~~02~~
**Gamma Globulins (%)**	Adult	19.00	0.31	Ad vs. Juv	6.00	4.37, 7.63	< 0.0~~00~~1
	Sub-Adult	18.00	0.45	Ad vs. SA	0.98	-0.22, 2.20	0.13
	Juvenile	13.00	0.65	SA vs. J	5.00	3.36, 6.68	< 0.~~00~~01

SE = standard error. Ad = adult. SA = sub-adult. Juv = juvenile.

Sex influenced the ~~AGR~~A:G, relative albumin, relative alpha 2 globulins, relative beta globulins, and absolute beta globulins (Table 4). Specifically, male turtles had higher ~~AGR~~A:G, relative albumin, and relative alpha 2 globulins, while females had higher absolute and relative beta globulin values. Gravid females had higher relative beta globulins and lower ~~AGR~~A:G, absolute and relative albumin, and alpha 2 globulins.

Table 4. Protein electrophoresis values that vary by sex and gravidity in free-living Blanding's turtles (*Emydoidea blandingii*).

Formatted: Font: Bold

Model estimates were produced by top-ranking general linear mixed models (see Table 2). SE = standard error.

Predictor	Analyte	Level	Model Estimate	SE	Difference	95% CI	P - value
**Sex**	**Albumin (%)**	Male	20.40	0.60	1.21	0.31 - 2.11	0.01~~09~~
		Female	19.20	0.50			
	**Albumin/Globulin**	Male	0.30~~296~~	0.01	0.02~~3~~	0.01~~08~~ - 0.04~~38~~	0.01~~04~~
		Female	0.27~~3~~	0.01			
	**Alpha 2 Globulins (%)**	Male	16.20	0.50	1.60	0.72 - 2.40	0.01~~003~~
		Female	14.60	0.43			
	**Beta Globulins (g/~~d~~L)**	Male	12.~~.2~~30	0.~~0~~90	1.~~0.1~~80	0.~~0~~50 ~~-~~– 3.~~0.3~~20	0.01
		Female	14.~~.4~~10	0.~~0~~80			
	**Beta Globulins (%)**	Male	34.50	0.97	3.04	1.56 - 4.53	< 0.0~~00~~1
		Female	37.60	0.92			
**Gravidity**	**Albumin/Globulin[a]**	Non-Gravid	0.31~~05~~	0.01~~07~~	0.03~~1~~	0.02 - 0.06	0.04
		Gravid	0.27~~4~~	0.02~~17~~			
	**Albumin (g/~~d~~L)**	Non-Gravid	7.~~0.7~~10	0.~~0~~30	1.~~0.1~~20	0.~~0~~03 -2. ~~0.2~~30	0.04
		Gravid	6.00~~0.6~~	0.~~0~~60			
	**Albumin (%)**	Non-Gravid	21.00	0.32	2.42	0.88 - 3.95	0.01~~02~~
		Gravid	18.60	0.84			
	**Alpha 2 Globulins (%)**	Non-Gravid	16.40	0.28	1.90	0.63 - 3.24	0.01~~04~~
		Gravid	14.40	0.70			
	**Beta Globulins (%)**	Non-Gravid	34.40	0.73	3.40	1.07 - 5.71	0.01~~05~~
		Gravid	37.80	1.30			

SE = standard error.

[a] Gravidity was not included in the top-ranking model for Albumin/Globulin due to confounding with the "cloaca" variable, but considered separately it is significantly associated with Albumin/Globulin

Multiple EPH fractions were associated with health predictors. Body condition was negatively associated with TP, absolute beta globulins, and absolute gamma globulins (Fig 2). Turtles with predator injuries or erosions of the plastron had higher relative and absolute beta globulins than those with normal plastrons (Table 1). Abnormalities of the appendicular system were associated with higher TP and absolute albumin, beta globulins, and gamma globulins (Table 5). Integumentary abnormalities were associated with lower TP and absolute beta globulins (Table 5). Turtles with upper respiratory disease had lower absolute and relative prealbumin than those without (Table 5). Finally, cloacal abnormalities were associated with a lower ~~AGR~~ A:G (Table 5). Top models for each EPH fraction tended to include spatiotemporal, demographic, and health predictors, highlighting the influence that each of these components has on the distribution of blood proteins in Blanding's turtles (Table 2).

Table 5. Protein electrophoresis values that vary based on the presence of physical examination abnormalities in free-living Blanding's turtles (*Emydoidea blandingii*). Model estimates were produced by top-ranking general linear mixed models (see Table 2). Top models for relative and absolute alpha 1 globulins contained "carapace", however no contrasts for this predictor were significant therefore not reported.

Predictor	Analyte	Level	Model Estimate	SE	Difference	95% CI	P - value
**Appendages**	**Total Protein (g/dL)**	Normal	31..190	1.0.110	5.0.530	1.0.190 -– 8.00.86	0.0102
		Abnormal	37..710	1.0.180			
	**Albumin (g/dL)**	Normal	6.100.61	0.030	9.00.090	0.010 -1. 0.170	0.03
		Abnormal	0.7.00	0.050			
	**Beta Globulins (g/dL)**	Normal	12..210	0.070	2.0.220	0.050 -– 3.0.380	0.01
		Abnormal	14..420	1.000.1			
	**Gamma Globulins (g/dL)**	Normal	5.0.550	0.030	1.0.140	0.060 -– 2.0.210	0.01006
		Abnormal	6.0.680	0.040			
**Integument**	**Total Protein (g/dL)**	Normal	31..190	1.0.110	4.0.460	0.070 -– 8.0.850	0.02
		Abnormal	27..720	2.000.2			
	**Beta Globulins (g/dL)**	Normal	14..410	0.070	2.000.2	0.020 -– 3.0.380	0.03
		Abnormal	12..220	1.0.110			
**Upper Respiratory Disease**	**Prealbumin (g/dL)**	Absent	0.058	0.003	0.027	0.003 - 0.052	0.03
		Present	0.031	0.012			
	**Prealbumin (%)**	Absent	1.88	0.13	1.04	0.11 - 1.97	0.03
		Present	0.84	0.48			
**Cloaca**	**Albumin/Globulin**	Normal	0.3106	0.0106	0.044	0.014 - 0.072	0.0103
		Abnormal	0.263	0.0216			

Formatted: Font: Bold

SE = standard error.

Reference intervals were constructed in both a subject-based and population-based manner for each EPH analyte (Table 6). Based on the index of individuality, subject-based reference intervals are superior for absolute and relative alpha 1 globulins, absolute beta globulins, and relative albumin in Blanding's turtles. The remainder of the II values fell between 0.6 and 1.4, indicating that population-based reference intervals were employed and interpreted with caution for all other fractions.

Table 6. Summary data including data distribution, measure of central tendency (mean for normally distributed variables, median for non-normally distributed variables), measure of dispersion (standard deviation for normally distributed variables, 10th – 90th percentiles for non-normally distributed variables), and reference intervals for plasma protein electrophoresis in free-living, apparently healthy Blanding's turtles (*Emydoidea blandingii*).

Formatted: Font: Bold

Analyte	N	Dist	CT	Disp	Min	Max	Reference Interval	90% CI LB	90% CI UB	II	RCV (%)	Sub vs. Pop
**Total Protein (g/~~d~~L)**	193[a]	NG	31.00~~.1~~	18.00~~.8~~ ~~-~~– 47.~~.7~~50	1~~.~~3.00	56.00~~.6~~	15.~~.5~~70 ~~-~~– 52.~~.2~~30	15.~~.5~~40 ~~-~~– 17.~~.7~~60	4~~.~~8.60 ~~-~~– 53.~~.3~~60	0.68~~75~~	62.70	Pop
**Albumin/Globulin**	195[b]	G	0.29~~86~~	0.07~~66~~	0.14	0.46	0.17 - 0.43	0.16 - 0.19	0.40 - 0.46	0.66~~58~~	41.20	Pop
**Prealbumin (g/~~d~~L)**	196	NG	0.~~0~~40	0.~~0~~05 ~~-~~– 1.~~0.1~~20	0.00	2.~~0.~~2 30	0.00 ~~-~~– 2.00~~0.2~~	0.00 - 0.~~0~~10	1.~~0.1~~80 ~~-~~– 2.~~0.2~~3~~5~~0	0.90~~1~~	237.00	Pop
**Albumin (g/~~d~~L)**	196	NG	6.~~0.6~~30	3.~~0.3~~40 ~~-~~– 9.~~0.9~~90	1.~~0.1~~90	15.~~.5~~60	2.~~0.2~~50 ~~-~~– 13.~~.3~~40	2.~~0.2~~10 ~~-~~– 3.00~~0.3~~	1~~.~~2.90 - 1~~.~~5.00	0.66~~2~~	74.40	Pop
**Alpha 1 Globulins (g/~~d~~L)**	196	NG	1.~~0.1~~90	~~0.~~1.00 ~~-~~– 4.~~0.4~~30	0.~~0~~70	1~~.~~1.60	0.~~0~~80 ~~-~~– ~~0.~~5.70	0.~~0~~70 - 0.~~0~~80	4.~~0.4~~10 ~~-~~– 6.~~0.6~~10	0.54~~39~~	88.40	Sub
**Alpha 2 Globulins (g/~~d~~L)**	196	NG	~~0.~~4.70	~~0.~~2.70 ~~-~~– ~~0.~~7.40	~~0.~~1.70	11.~~1~~20	~~0.~~2.10 ~~-~~– 10.00	~~0.~~1.90 ~~-~~– 2.~~0.2~~50	~~0.~~9.60 - 1~~.~~0.70	0.67~~68~~	70.80	Pop
**Beta Globulins (g/~~d~~L)**	196	NG	1~~.~~0.80	~~0.~~5.04 - 1~~.~~7.90	~~0.~~3.40	30.00	~~0.~~4.00 - 2~~.~~5.70	~~0.~~3.30 ~~-~~– ~~0.~~4.40	21.~~.1~~80 - 2~~.~~9.90	0.56~~57~~	66.70	Sub
**Gamma Globulins (g/~~d~~L)**	195[c]	NG	~~0.~~5.60	~~0.~~2.80 ~~-~~– 10.~~.0~~70	~~0.~~1.40	1~~.~~6.50	~~0.~~1.70 - 1~~.~~4.10	~~0.~~1.30 ~~-~~– ~~0.~~1.90	1~~.~~2.30 - 1~~.~~5.70	0.99~~88~~	144.00	Pop
**Prealbumin (%)**	196	NG	1.00	0.00 - 4.20	0.00	10.10	0.00 - 6.70	0.00 ~~-~~– 1.00	4.90 - 7.80	0.93~~29~~	266.00	Pop
**Albumin (%)**	195[d]	G	20.30	3.80	13.00	31.00	14.00 ~~-~~– 29.00	13.00 ~~-~~– 15.00	27.00 ~~-~~– 31.00	0.59~~2~~	29.90	Sub
**Alpha 1 Globulins (%)**	196	NG	6.00	4.00 ~~-~~– 14.00	2.70	28.00	3.00 ~~-~~– 23.00	2.90 - 3.30	19.00 - 28.90	0.34~~38~~	51.80	Sub
**Alpha 2 Globulins (%)**	195[e]	NG	16.00	11.00 - 19.70	9.00	24.00	10.00 - 21.70	9.70 ~~-~~– 11.00	20.50 - 23.30	0.62~~4~~	31.20	Pop

**Beta Globulins (%)**	196	NG	35.80	25.00 - 49.10	18.00	59.10	20.00 - 55.20	19.00 – 21.00	51.90 - 57.70	0.82~~17~~	59.20	Pop
**Gamma Globulins (%)**	196	NG	19.00	11.30 – 28.00	8.00	37.00	9.700 - 32.10	9.40 – 10.00	31.20 - 34.10	1.20	114.00	Pop

Dist = distribution, NG = Non-Gaussian, G = Gaussian, CT = measure of central tendency, Disp = measure of dispersion, CI = confidence interval, LB = lower bound of reference interval, UB = upper bound of reference interval, II = index of individuality, RCV = reference change value, Sub = subject-based refence interval recommended, Pop = population-based reference interval recommended.

[a] Outliers removed: 10.00, 7~~.~~1.00, 7~~.~~4.00 g/~~d~~L

[b] Outliers removed: 0.51

[c] Outliers removed: 2~~.~~2.6 g/~~d~~L

[d] Outliers removed: 11%

[e] Outliers removed: 26%

Discussion

We set out to describe baseline plasma protein fractions in a well-studied population of Blanding’s turtles in northeastern Illinois and southeastern Wisconsin and observed EPH fractions varied significantly based on spatiotemporal, demographic, and health factors. Understanding how reptile clinical pathology values correlate to landscape changes is important for contextualizing health assessments and evaluating ecosystem wellness [1][2][5][6].

We documented several statistically significant inter-annual differences in Blanding’s turtle total protein and EPH fractions. These changes may be attributable to fluctuation in climactic variables that influence turtle metabolism and resource availability. Temperature is a key determinant of metabolic rates in ectotherms, including reptiles [41~~36~~][42~~37~~], and previous studies in loggerheads (*Caretta caretta*) and green turtles (*Chelonia mydas*) ~~and~~ observed a negative correlation with the ~~AGR~~ A:G and environmental temperature [1~~36~~8]. In Lake County, the average air temperatures in May (60.4°F/15.8°C) and June (66.4°F/19.1°C) of 2018 were warmer than those in May (54.7°F/12.6°C) and June (64.2°F/17.9°C) of 2019 [43~~39~~]. Similar to the temperature-associated protein changes in sea turtles, Blanding’s turtle TP, albumin, alpha 1 and alpha 2 globulins, and gamma globulin concentrations were greater in 2018, while prealbumin, beta globulin, and ~~AGR~~ A:G were greater in 2019. It is plausible that turtles would be more active, consume more food items, initiate reproductive activity, and mount immune responses more efficiently at higher temperatures, all of which may increase circulating protein concentrations and contribute to the observed inter-annual variability in EPH fractions [41~~36~~].

Temperature, however, is not the only environmental factor that differed between years. Rainfall was also greater during May and June of 2018 (30.2 inches total) compared to the same time period in 2019 (18.76 inches total), while humidity was a bit more consistent between years (77.2% average relative humidity in May and June of 2018 and 80.75% average relative

humidity in May and June of 2019) [43~~39~~]. Water availability and humidity both influence behavioral thermoregulation in ectotherms and can modify their activity levels in complex ways [44~~40~~]. Increased activity secondary to rainfall may create more opportunities for antigenic stimulation and contribute to changes in food consumption, reproductive behaviors, and other physiologic processes with a resultant increase in plasma protein concentrations [44]. While the effects of some climactic variables on ectotherm physiology have been at least partially characterized, many other environmental variables may also impact resource availability and overall wellness. Additional research is needed to determine the underlying environmental causes of temporal variability in reptile protein electrophoresis values. It is likely that inter-annual differences in protein electrophoresis values also exist for other reptiles [45]. Unfortunately, direct comparison to existing literature is difficult, because although some multi-year chelonian EPH studies exist [4][10][16][18][46][47][48][49][50][51][52], the possibility of inter-annual variation in EPH values is infrequently assessed.

~~The exact cause of the observed inter-annual variability is unknown; however, the effects of year were as significant as those attributed to demographic or health factors, indicating that this is an important source of biological variation in Blanding's turtle EPH values. It is likely that inter-annual differences in protein electrophoresis values exist for other reptiles as well, as many reptilian clinical pathology values vary by year [41]. Unfortunately, direct comparison to existing literature is difficult, because although some multi-year chelonian EPH studies exist [4][8][13][38][42][43][44][45][46][47][48], the possibility of inter-annual variation in EPH values is infrequently assessed.~~ Our findings indicate that future EPH studies in reptiles should consider the potential for significant inter-annual effects.

Formatted: Normal (Web)

TP and absolute EPH fractions differed by location in Blanding's turtles, similar to eastern box turtles (*Terrapene carolina carolina*) [4], alligator snapping turtles (*Macrochelys*

temminckii) [46~~42~~] and green turtles [45~~41~~]. SBCP and IBSP vary significantly in their habitats despite their close proximity. SBCP offers a coastal dune and swale habitat that has been managed and preserved since 2004, whereas IBSP includes adjacent north (N) and south (S) units containing dune, prairie, oak-savannah and wetland habitats, with limited management prior to 2017 [22~~19~~]. The effect of location on EPH parameters depended on the year; and the differences in EPH values between the study sites and years were rarely consistent. The only consistent location-related differences ~~that remained similar~~ between 2018 and 2019 were the cold spots ~~(areas with lower plasma protein concentrations)~~ at the northern end of SBCP, potentially indicating a more consistent health status for turtles occupying this area. Inter-annual fluctuation in TP and EPH fractions between and within the different study sites may indicate transient, localized changes in health status with unclear management implications. Focused, longitudinal assessment of telemetered individuals within these areas will be useful to identify biotic and abiotic factors associated with acute changes in EPH parameters. Our findings indicate that it is important to consider both location and time at multiple different scales in order to obtain a nuanced understanding of the drivers of turtle health status.

Several Blanding's turtle plasma protein values were affected by month; specifically, ~~AGR~~A:G, absolute albumin, relative albumin, and both absolute and relative prealbumin values peaked in June, while relative gamma globulins were highest in May. Similar patterns have been documented in other chelonians including Hermann's tortoises (*Testudo hermanni*) [53~~49~~], alligator snapping turtles [46~~42~~], and eastern box turtles [4]. These changes may be attributed to increased interactions with other turtles during the mating season and/or~~,~~ reproductive physiology. In the Lake Plain, Blanding's turtle mating season occurs from March to May, while nesting season typically begins in June. Turtles are more likely to interact with each other and potentially transmit pathogens such as *Emydoidea* herpesvirus 1 during mating season [54~~50~~],

which may contribute to an increase in gamma globulins (i.e. immunoglobulins). Alternatively, the increased gamma globulin concentration in May could be secondary to vitellogenesis, as the release of estrogen stimulates hyperglobulinemia in chelonians [55~~51~~][56~~52~~]. Elevated albumin concentrations in June may be attributable to dehydration associated with prolonged overland trips and nesting-associated exertion, similar to previous reports in ~~loggerheads~~ sea turtle species [48~~44~~][57~~53~~][58~~54~~][59]. Studies in other chelonian and lizard species have documented elevations in albumin and total proteins during the summer months and have correlated these elevations to the increased food consumption and reproductive activity in this time period [4][60~~55~~][61~~56~~].

Gravid females had higher relative beta globulins and lower ~~AGR~~A:G, absolute and relative albumin, and alpha 2 globulins. As reviewed above, gravid reptiles can develop hyperglobulinemia during vitellogenesis in response to estrogen [62~~57~~]. A study conducted in pond sliders (*Trachemys scripta*) demonstrated that estrogen also downregulates albumin, ~~this~~ which may be a factor in the lower albumin concentrations observed in gravid female Blanding's turtles [63~~58~~]. Higher globulins and lower albumin secondary to estrogen production would also support the lower ~~AGR~~ A:G in gravid females. In birds, elevated beta globulins ~~levels~~ are attributed to egg production [64~~59~~]. In leatherbacks, alpha 2 globulins decrease over the nesting season due to inanition [65~~60~~]. Our findings are important to provide context for future studies on EPH in gravid chelonians, as several of the changes associated with gravidity (elevated beta globulins, lower albumin and ~~AGR~~A:G) can also be interpreted as indicative of inflammation and poor health [17].

Male turtles had higher ~~AGR~~A:G, relative albumin, and relative alpha 2 globulins while females had higher absolute and relative beta globulin values. Many of these findings differ from those in other chelonians. Relative albumin was higher in female red-eared sliders (*Trachemys*

scripta elegans) and map turtles (*Graptemys geographica*) [6661], and absolute albumin was higher in female eastern box turtles [4]. Male loggerhead sea turtles had higher absolute and relative beta globulin concentrations, although there was a great deal of beta-gamma bridging indicating possible underlying disease processes in those individuals [108]. Male radiated tortoises had higher relative alpha 2 globulins during winter sampling, and female eastern box turtles and radiated tortoises had higher absolute and relative beta globulin concentrations [4][6762]. Consistent with a previous study in this population of Blanding's, but contrary to several other studies in chelonians, there was no difference in total protein between the sexes [2463]. Many of these findings may be confounded by the timing of our sampling, since it was concentrated during the breeding and nesting season. It is possible that if these turtles were sampled later in the year we would find that sex-based differences in Blanding's turtles are more in line with what is reported for other species. Blanding's turtles have some unique sex-associated EPH patterns compared to other chelonians, underscoring the need for studies like this to understand species differences in clinical pathology values.

Protein fraction concentrations varied across age groups, with juvenile turtles having the highest values for AGRA:G, relative albumin, relative alpha 1 globulins, and absolute alpha 1 globulins. Similar trends are seen in juvenile loggerheads [1368], juvenile Kemp's ridley sea turtles (*Lepidochelys kempii*) [6864][69], and juvenile gopher tortoises (*Gopherus polyphemus*) [65][70]. Adults had the highest values for relative beta globulins, relative gamma globulins, and absolute gamma globulins, which are similar to findings in other chelonians. Adult loggerheads [108], eastern box [4], and green turtles [4945] all had higher absolute beta globulin levels, and adult Kemp's ridley sea turtles [60] [69] had higher absolute beta and gamma globulins compared to their juvenile counterparts. In general, the changes found can be attributed to

Formatted: Strikethrough

Formatted: Strikethrough

increased antigenic challenge as turtles age and become reproductively mature, a consistent finding in other chelonian studies [165][196][46].

The overall health status of individuals also contributed to variations in plasma protein concentrations. Higher absolute beta and gamma globulins were found in turtles with lower body fat percentage, which likely reflects poor health in these individualsboth increase in the presence of acute and chronic inflammation. BCS has proven to beis a reliable measure of health status in other reptiles, with a good body condition score equating to better immune function and capability to fight periodic bouts of disease [7166]. The elevated relative and absolute beta globulins in turtles with plastron injuries and erosions might be due to the plastron constantly being in contact with either unclean water or the ground, increasing the potential for chronic antigenic stimulation when injuries or abnormalities are present.

Appendage abnormalities were associated with higher TP and absolute beta and gamma globulins, indicating the presence of possible chronic inflammation. The loss of nails or digits from a variety of causes results in open wounds where infections may develop. Missing nails and digits might also have an impact on the turtle’s overall health, making tasks like swimming and foraging more difficult [72]. In eastern box turtles, microvascular problems and primary microbial infections can cause the loss of digits and nails, indicating that even apparently minor anatomical abnormalities may have physiologically significant impacts on these turtles [7367]. Integument abnormalities were associated with lower TP and absolute beta globulin concentrations. A study conducted in green and loggerhead sea turtles with traumatic wounds to their carapace, head, and/or flippers showed similar trends, with those experiencing a trauma having lower beta globulin concentrations than their healthy counterparts [1368].

Turtles with evidence of upper respiratory disease had lower relative prealbumin concentrations. Like albumin, prealbumin is a negative acute phase protein and decreases in the

Formatted: Font: (Default) Times New Roman

presence of inflammation [74~~68~~]. Prealbumin concentrations can also be lower in cases of protein malnutrition [74~~68~~]. These turtles may have lower concentrations because they are ill with upper respiratory infection, and this illness could be preventing them from taking in an adequate amount of protein. It is important to note, however, that prealbumin has not yet been validated in chelonian species, so it is unclear if prealbumin is truly what is represented in the prealbumin region of the electrophoretogram. The lower ~~AGR~~ A:G in turtles with cloacal abnormalities likely ~~has to do~~consistent with inflammation associated with infection, inflammation, or stress, with a lower ratio usually indicating hyperglobulinemia [75~~69~~].

The population of Blanding's turtles that was studied showed a high degree of within-individual variability in EPH parameters at different points in time. This was also identified for hematologic and plasma biochemical parameters in the same population [24~~63~~]. Reptile clinical pathology parameters are widely variable, and it is important to understand the many factors come together to influence the ~~final~~ absolute value of each analyte~~s~~. Our findings in Blanding's turtles indicate that the index of individuality and subsequent need for subject-based reference intervals should be investigated in other reptile species in order to improve the interpretation of clinical pathology testing.

There are a few limitations in this study that could be addressed in future research. Due to radiotelemetry strategy in the Lake County location, there was a bias towards adult female turtles, which are followed closely to identify nest location. Additionally, a limited number of overtly unhealthy turtles were identified, with only a few individuals having physical examination abnormalities. While this is recognized as a positive finding considering that it indicates the population is doing well, it does limit the ability to determine how EPH values change in states of poor health. Furthermore, turtles that could be identified more than once over the course of the sampling period were sampled every three to six weeks, which could indicate

the fluctuation of an inflammatory response or antigenic stimulation over time.~~. Due to the fact they some were sampled at multiple different points in their disease processes, some EPH changes may have been fluctuating in line with their inflammatory response.~~

All blood samples were obtained from the subcarapacial sinus. Blood and lymphatic vessels are very closely associated with one another at this site, making it possible that blood samples could become contaminated with lymphatic fluid during venipuncture [76~~70~~]. While samples with obvious lymph contamination were discarded, undetectable lymph contamination could have negatively impacted results. Lymph contamination has been known to falsely decrease PCV and hemoglobin concentrations and may have similar affects on plasma protein concentrations [77~~71~~]. For those turtles that were collect and sampled from traps, the stress of trapping could ultimately play a role in affecting plasma protein fractions, [50] but the significance has not been studied in Blanding's turtles. Timing samples to be collected pre- or post-prandial to account for lipemia is difficult to control in wildlife research, but a study conducted in Kemp's ridley and green sea turtles revealed that feeding had very minimal effects on plasma biochemical values and are therefore unlikely to alter clinical interpretation [78].

There is a discrepancy between the use of TS verses total protein (TP). A refractometer is an efficient way to measure TS in a field research setting, but TS includes both plasma proteins as well as additional plasma solutes [79]. While there have been multiple studies conducted in other chelonian species that show a significant correlation between TS and TP [80][19], a direct relationship in Blanding's turtles has not been previously identified. Following separation, the collected plasma was frozen until protein electrophoresis could be run. Studies conducted in other reptile species have identified EPH differences in fresh plasma compared to frozen/thawed samples, and some of those studies recommended using fresh samples for best results [81~~72~~][13~~1~~]. In our circumstances, it would have been impractical and cost-prohibitive to ship

over 200 fresh plasma samples for analysis separately, underscoring the need for us to freeze and batch-run our samples. Hemolysis also has the potential to affect EPH values, but no grossly hemolyzed samples were use in this study [8273].

The baseline plasma protein reference intervals generated in this study will be useful in defining the health status of this population. There is some overlap in the top models for predicting relative and absolute EPH fractions;, however there also instances where the values vary, with absolute values being high while relative values are low for the same variable. This variation demonstrates the importance of considering both relative and absolute fractions when interpreting EPH values because they might be driven by different processes. Results of this study validate that month, location, sex, age class, and health status should be considered when interpreting EPH fractions. Although EPH does not provide details on specific diseases or stressors, it is a helpful tool that can aid in identifying when intervention and treatment might be needed. With the increased use of protein electrophoresis to evaluate the health status of animals in the veterinary medical field, the application of this tool in conservation of wild populations is becoming more widely accepted and studied. With baseline concentrations established and evaluated for variation, future studies can aim to validate and expand upon the normal reference intervals in this species.

References

1) Deem SL, Karesh WB, Weisman W. Putting theory into practice: Wildlife health in conservation. Conserv Biol. 2008;15(5): 1224-1233. doi:10.1111/j.1523-1739.2001.00336.x.

2) Ryser-Degiorgis MP. Wildlife health investigations: needs, challenges and recommendations. BMC Vet Res. 2013;9(1): 223. doi: 10.1186/1746-6148-9-223.

3) Mörner T, Obendorf DL, Artois M, Woodford MH. Surveillance monitoring of wildlife diseases. Rev Sci Tech. 2002;21(1): 67-76. doi: 10.20506/rst.21.1.1321.

4) Flower JE, Byrd J, Cray C, Allender MC. Plasma electrophoretic profiles and hemoglobin binding protein reference intervals in the eastern box turtle (*Terrapene carolina carolina*) and influences of age, sex, season, and location. J Zoo Wildl Med. 2014;45(4): 836-842. doi: 10.1638/2014-0035.1.

5) Aguirre AA, Lutz PL. Marine turtles as sentinels of ecosystem health: Is fibropapillomatosis an indicator? Ecohealth. 2004;1: 275-283. doi: 10.1007/s10393-004-0097-3.

6) Page-Karijian A, Perrault JR. Sea turtle health assessments: Maximizing turtle encounters to better understand health. In: Nahill B, editor. Sea turtle research and conservation: Lessons from Working in the Field. Elsevier, Academic Press; 2021. pp. 31-44.

7) Knotek Z, Musilova A, Pinterova K, Knotkova Z. Plasma protein electrophoresis as a diagnostic tool for endangered Asian reptiles. World Small Animal Veterinary Association World Congress Proceedings. 2015. Available from https://www.vin.com/apputil/content/defaultadv1.aspx?id=7259164&pid=14365&print=1

8) Allender MC, Junge RE, Baker-Wylie S, Hileman ET, Faust LJ, Cray C. Plasma electrophoretic profiles in the eastern massasauga (*Sistrurus catenatus*) and influences of

Formatted: Not Highlight

Formatted: Not Highlight

age, sex, year, location, and snake fungal disease. J Zoo Wildl Med. 2015;46(4): 767-773. doi: 10.1638/2015-0034.1.

9) Cooper-Bailey K, Smith SA, Zimmerman K, Lane R, Raskin RE, Denardo D. Hematology, leukocyte cytochemical analysis, plasma biochemistry, and plasma electrophoresis of wild-caught and captive-bred Gila monsters (*Heloderma suspectum*). Vet Clin Pathol. 2011;40(3): 316-323. doi: 10.1111/j.1939-165x.2011.00337.x.

10) Gicking JC, Foley AM, Harr KE, Raskin RE, Jacobson E. Plasma protein electrophoresis of the Atlantic loggerhead sea turtle, *Carretta carretta*. J Herpetol Med Surg. 2004;14(3): 13-18. doi: 10.5818/1529-9651.14.3.13.

11) Giménez M, Saco Y, Pato R, Busquets A, Martorell JM, Bassols A. Plasma protein electrophoresis of *Trachemys scripta* and *Iguana iguana*. Vet Clin Pathol. 2010;39(2): 227-235. doi: 10.1111/j.1939-165x.2009.00204.x.

12) Machado CC, Silva LFN, Ramos PRR, Takahira RK. (2006). Seasonal influence on hematologic values and hemoglobin electrophoresis in Brazilian *Boa constrictor amarali*. J Zoo Wildl Med. 2006;37(4): 487–491. doi: 10.1638/05-124.1.

13) Proverbio D, Bagnagatti De Giorgi G, Della Pepa A, Baggiani L, Spada E, Perego R, et al. Preliminary evaluation of total protein concentration and electrophoretic protein fractions in fresh and frozen serum from wild Horned Vipers (*Vipera ammodytes ammodytes*). Veterinary Clinical Pathology. 2012;41(4): 582-586. doi: 10.1111/j.1939-165x.2012.00486.x.

14) Silva LFN, Riani-Costa CCM, Ramos PRR, Takahira RK. Seasonal influence on biochemical profile and serum protein electrophoresis for *Boa constrictor amarali* in captivity. Braz J Biol. 2011;71(2): 517-520. doi: 10.1590/s1519-69842011000300023.

15) Work TM, Rameyer RA, Balazs GH, Cray C, Chang SP. Immune status of free-ranging green turtles with fibropapillomatosis from Hawaii. J Wildl Dis. 2001;37(3): 574-581. doi: 10.7589/0090-3558-37.3.574.

16) Osborne AG, Jacobson ER, Bresette MJ, Singewald DA, Scarpino RA, Bolten AB. Reference intervals and relationships between health status, carapace length, body mass, and water temperature and concentrations of plasma total protein and protein electrophoretogram fractions in Atlantic loggerhead sea turtles and green turtles. J Am Vet Med Assoc. 2010;237(5): 561-567. doi:10.2460/javma.237.5.561.

17) Cray, C. Acute phase proteins in animals. Prog Mol Biol Transl Sci. 2012;105: 113-150. doi:10.1016/b978-0-12-394596-9.00005-6.

18) Innis CJ, Tlusty M, Wunn D. Hematologic and plasma biochemical analysis of juvenile head-started northern red-bellied cooters (*Pseudemys rubriventris*). J Zoo Wildl Med. 2007;38(3): 425-432. doi:10.1638/1042-7260(2007)38[425:hapbao]2.0.co;2.

19) Rousselet E, Stacy NI, Lavictoire K, Higgins BM, Tocidlowski ME, Flanagan JP, et al. Hematology and plasma biochemistry analytes in five age groups of immature, captive-reared loggerhead sea turtles (*Caretta caretta*). J Zoo Wildl Med. 2013;44(4): 859-874. doi:10.1638/2012-0162r1.1.

20) Congdon JD, Sels RC. Relationships of reproductive traits and body size with attainment of sexual maturity and age in Blanding's turtles (*Emydoidea blandingii*). J Evol Biol. 1993;6(4): 547-557. doi:10.1046/j.1420-9101.1993.6040547.x.

21) van Dijk PP, Rhodin AGJ. *Emydoidea blandingii*. The IUCN Red List of Threatened Species. 2011. Available from https://dx.doi.org/10.2305/IUCN.UK.2011-1.RLTS.T7709A155088836.en.

22) Glowacki G. Blanding's Turtle recovery program: 2017 Summary Report. Lake County Forest Preserve District, Blanding's Turtle Recovery Program. 2017.

23) Kuhns AR. Recovery of the Blanding's turtle (Emydoidea blandingii) at Spring Bluff Nature Preserve, Lake Count Forest Preserves. Lake County 552 Forest Preserve District, Blanding's Turtle Recovery Program Federal Aid Project T-39-D-1. 2010. Available from: https://www2.illinois.gov/dnr/conservation/iwap/documents/swgreports/t-39%20d-1%20final%20recovery%20of%20the%20blandings%20turtle.pdf

24) Mumm LE, Winter JM, Andersson KE, Glowacki GA, Adamovicz LA, Allender MC. Hematology and plasma biochemistries in the Blanding's turtle (*Emydoidea blandingii*) in Lake County, Illinois. PloS One. 2019;14(11). doi: 10.1371/journal.pone.0225130.

25) Gutzke WH, Packard GC. The influence of temperature on eggs and hatchlings of Blanding's turtles, *Emydoidea blandingii*. J Herpetol. 1987;21(2): 161. doi:10.2307/1564476.

26) Ernst CH, Lovich JE. *Emydoidea blandingii*, Blanding's Turtle. In: Turtles of the United States and Canada. 2nd ed. Baltimore: The John Hopkins University Press; 2009. pp. 233–249.

27) Mosimann JE, Bider JR. Variation, sexual dimorphism, and maturity in a Quebec population of the common snapping turtle, *Chelydra serpentina*. Can J Zool. 1960;38:19–38.

28) Newman E, Allender M, Thompson D, Glowacki G, Ivančić M, Adkesson M, et al. Measuring fat content using computed tomography to establish a body condition index in free-ranging Blanding's turtles (*Emydoidea blandingii*) in Illinois. J Zoo Wildl Med. 2019;50(3): 594-603. doi: 10.1638/2018-0154.

29) R Core Team. 2020. R: A language and environment for statistical computing. R Foundation for Statistical Computing, Vienna, Austria. https://www.R-project.org/.

30) Bates D, Maechler M, Bolker B, Walker S. Fitting linear mixed-effects models using lme4. J Stat Softw. 2015;67(1): 1-48. doi:10.18637/jss.v067.i01.

31) Kuznetsova A, Brockhoff PB, Christensen RHB. lmerTest package: Tests in linear mixed effects models. J Stat Softw. 2017; 82(13): 1-26. doi: 10.18637/jss.v082.i13.

32) Lenth RV. Least-squares means: The R package ismeans. J Stat Softw. 2016;69(1): 1-33. doi: 10.18637/jss.v069.i01.

33) Fox J, Weisberg S. An {R} Companion to Applied Regression. Sage Publications, USA. 2011. Available from http://socserv.socsci.mcmaster.ca/jfox/Books/Companion.

34) Barton K. MuMIn: Multi-Model Inference. R package version 1.43.15. 2019. Available from https://CRAN.R-project.org/package=MuMIn.

35) Lüdecke D. ggeffects: Tidy data frames of marginal effects from regression models. J Open Source Softw. 2018;3(26): 772. doi: 10.21105/joss.00772.

36) Friedrichs KR, Harr KE, Freeman KP, Szladovits B, Walton RM, Barnhart KF, et al. ASVCP reference interval guidelines: determination of de novo reference intervals in veterinary species and other related topics. Vet Clin Pathol. 2012;41: 441–453. doi:10.1111/vcp.12006.

37) Walton RM. Subject-based reference values: biological variation, individuality, and reference change values. Vet Clin Pathol. 2012;41:175–181.

38) Harris EK. Effects of intra- and interindividual variation on the appropriate use of normal ranges. Clin Chem. 1974;20:1535–1542.

39) Finnegan D. referenceIntervals: Reference Intervals. R package version 1.1.1. 2004. Available from https://CRAN.R-project.org/package=referenceIntervals.

40) Horn PS, Feng L, Li Y, Pesce AJ. Effect of outliers and nonhealthy individuals on reference interval estimation. Clin Chem. 2001;47: 2137-2145.

41) Shine R. Life-history evolution in reptiles. Annu Rev Ecol Ecol Syst. 2005;36(1): 23-46. doi:10.1146/annurev.ecolsys.36.102003.152631.

42) Huey R, Slatkin M. 1976. Costs and benefits of lizard thermoregulation. Q. Rev. Biol. 51:363–84

43) NOAA. National Centers for Environmental Information; 2020. US Local Climatological Data. NCEI DSI 3505. Available from https://www.ncdc.noaa.gov/cdo-web/datasets/LCD/stations/WBAN:04845/detail.

44) Rozen- Rechels D, Dupoué A, Lourdais O, Chamaillé- Jammes S, Meylan S, Clobert J, et al. When water interacts with temperature: Ecological and evolutionary implications of thermo- hydroregulation in terrestrial ectotherms. Ecol Evol. 2019;9(17): 10029-10043. doi:10.1002/ece3.5440.

45) Flint M, Brand A, Bell IP, Hof CA. Monitoring the health of green turtles in northern Queensland post catastrophic events. Sci Total Environ. 2019;660: 586-592. doi:10.1016/j.scitotenv.2019.01.065.

46) Chaffin K, Norton TM, Gilardi K, Poppenga R, Jensen JB, Moler P, et al. Health assessment of free-ranging alligator snapping turtles (*Macrochelys temminckii*) in Georgia and Florida. J Wildl Dis. 2008;44(3): 670-686. doi:10.7589/0090-3558-44.3.670.

47) Deem SL, Dierenfeld ES, Sounguet GP, Alleman AR, Cray C, Poppenga RH, et al. Blood values in free-ranging nesting leatherback sea turtles (*Dermochelys coriacea*) on the coast of the Republic of Gabon. J Zoo Wildl Med. 2006;37(4): 464-471. doi:10.1638/05-102.1.

48) Deem SL, Norton TM, Mitchell M, Segars A, Alleman AR, Cray C, et al. Comparison of blood values in foraging, nesting, and stranded loggerhead turtles (*Caretta caretta*) along

the coast of Georgia, USA. J Wildl Dis. 2009;45(1): 41-56. doi:10.7589/0090-3558-45.1.41.

49) Flint M, Matthews BJ, Limpus CJ, Mills PC. Establishment of reference intervals for plasma protein electrophoresis in Indo-Pacific green sea turtles, *Chelonia mydas*. Conserv Physiol. 2015;3(1). doi: 10.1093/conphys/cov037.

50) Innis C, Merigo C, Dodge K, Tlusty M, Dodge M, Sharp B, et al. Health evaluation of leatherback turtles (*Dermochelys coriacea*) in the northwestern Atlantic during direct capture and fisheries gear disentanglement. Chelonian Conserv Biol. 2010;9(2): 205-222. doi:10.2744/ccb-0838.1.

51) Page-Karjian A, Rivera S, Torres F, Diez C, Moore D, Dam RV, et al. Baseline blood values for healthy free-ranging green sea turtles (*Chelonia mydas*) in Puerto Rico. Comp Clin Path. 2014;24(3): 567-573. doi:10.1007/s00580-014-1947-1.

52) Perrault JR, Miller DL, Eads E, Johnson C, Merrill A, Thompson LJ, et al. Maternal health status correlates with nest success of leatherback sea turtles (*Dermochelys coriacea*) from Florida. PLoS One. 2012;7(2). doi:10.1371/journal.pone.0031841.

53) Andreani G, Carpenè E, Cannavacciuolo A, Girolamo ND, Ferlizza E, Isani G. Reference values for hematology and plasma biochemistry variables, and protein electrophoresis of healthy Hermann's tortoises (*Testudo hermanni* ssp). Vet Clin Pathol. 2014;43(4): 573-583. doi:10.1111/vcp.12203.

54) Lindemann D, Allender M, Thompson D, Glowacki G, Newman E, Adamovicz L, et al. Epidemiology of Emydoidea herpesvirus 1 in free-ranging Blanding's turtles (*Emydoidea blandingii*) from Illinois. J Zoo Wildl Med. 2019;50(3): 547-556. doi: 10.1638/2018-0074.

55) Campbell TW. Clinical pathology of reptiles. In: Mader DR, editor. Reptile Medicine and Surgery. St. Louis: Saunders Elsevier; 2006. pp 453-470.

56) Dessauer HC. (1970). Blood chemistry of reptiles: Physiological and evolutionary aspects. In: Gans C, Parsons TS, editors. Biology of the Reptilia, Vol. 3. New York: Academic Press; 1970. pp. 1-72.

57) Dowling Z, Hartwig T, Kiviat E, Keesing F. Experimental management of nesting habitat for the Blanding's turtle (*Emydoidea blandingii*). Ecological Rest. 2010;28(2): 154-159. doi:10.3368/er.28.2.154.

58) Congdon J, Kinney O, Nagle R. Spatial ecology and core-area protection of Blanding's Turtle (*Emydoidea blandingii*). Can J Zool. 2011;89: 1098-1106. doi: 10.1139/z11-091.

59) Price ER, Sotherland PR, Wallace BP, Spotila JR, Dzialowski EM. Physiological determinants of the interesting interval in sea turtles: a novel 'water-limitation' hypothesis. Biol Lett. 2019;12(20190248). doi: 10.1098/rsbl.2019.0248.

60) Yang PY, Yu PH, Wu SH, Chie CH. Seasonal hematology and plasma biochemistry references range values of the yellow-marginated box turtle (*Cuora flavomarginata*). J Zoo Wildl Med. 2014;45:278-286. doi: 10.1638/2013-0125R1.1.

61) Musilová A, Knotková Z, Pinterová K, Knotek Z. Variations of plasma protein electrophoresis in healthy captive green iguanas (*Iguana iguana*). Vet Clin Pathol. 2015;44(2): 243-248. doi: 10.1111/vcp.12238.

62) Mader DR, Divers SJ. Current therapy in reptile medicine and surgery. St. Louis: Saunders Elsevier; 2004.

63) Selcer KW, Palmer BD. Estrogen downregulation of albumin and a 170-kDa serum protein in the turtle, *Trachemys scripta*. Gen Comp Endocrinol. 1995;97(3): 340-352. doi: 10.1006/gcen.1995.1034.

64) Harris DJ. Clinical Tests. In: Tully T, Dorrestien G, Jones A, editors. Handbook of Avian Medicine, 2nd ed. Philadelphia: Elsevier; 2009. pp. 77–84.

65) Perrault JR, Wyneken J, Page-Karjian A, Merrill A, Miller DL. Seasonal trends in nesting leatherback turtle (*Dermochelys coriacea*) serum proteins further verify capital breeding hypothesis. Conserv Physiol. 2014;2(1). doi: 10.1093/conphys/cou002.

66) Leineweber C, Öfner S, Stöhr AC, Marschang RE, Mathes K. A comparison of thyroid hormone levels and plasma capillary zone electrophoresis in red- eared sliders (*Trachemys scripta elegans*) and map turtles (*Graptemys* spp.) depending on season and sex. Vet Clin Pathol. 2019;49(1): 78-90. doi:10.1111/vcp.12838.

67) Zaias J, Norton T, Fickel A, Spratt J, Altman NH, Cray C. Biochemical and hematologic values for 18 clinically healthy radiated tortoises (*Geochelone radiata*) on St Catherines Island, Georgia. Vet Clin Pathol. 2006;35(3): 321-325. doi:10.1111/j.1939-165x.2006.tb00139.x.

68) Perrault JR, Schmidt JR, Walsh CJ, Yordy JE, Tucker AD. Brevetoxin exposure, superoxide dismutase activity and plasma protein electrophoretic profiles in wild-caught Kemp's ridley sea turtles (*Lepidochelys kempii*) in southwest Florida. Harmful Algae. 2014;37: 194-202. doi:10.1016/j.hal.2014.06.007.

69) Perrault JR, Arendt MD, Schwenter JA, Byrd JL, Harms CA, Cray C, et al. Blood analytes of immature Kemp’s ridley sea turtles (*Lepidochelys kempii*) from Georgia, USA: reference intervals and body size correlations. Conserv Physiol. 2020;8(1). doi: 10.1093/conphys/coaa091.

70) Page-Karjian A, Rafferty K, Clerson X, Stacy NI, Moore JA, Hirsch SE, et al. Comprehensive health assessment and blood analyte reference intervals of gopher

tortoises (*Gopherus polyphemus*) in southeastern FL, USA. Conserv Physiol. 2021;9(1). doi: 10.1093/conphys/coab015.

71) Lamberski N, Braun J, Witte C, Christopher M, Field K, Averill-Murray R, et al. Identifying key clinical signs and validating body condition scores to minimize disease spread and maximize individual survival during desert tortoise translocations. Joint Annual Meeting for Wildlife Disease Association and European Association of Wildlife Diseases. 2012. Available at: https://www.researchgate.net/publication/271519082_Identifying_key_clinical_signs_and_validating_body_condition_scores_to_minimize_disease_spread_and_maximize_individual_survival_during_desert_tortoise_translocations.

72) Fujii JA, McLeish D, Brooks AJ, Gaskell J, Van Houtan KS. Limb-use foraging marine turtles, an evolutionary perspective. PeerJ. 2018;6. doi: 10.7717/peerj.4565.

73) Adamovicz L, Allender MC, Archer G, Rzadkowska M, Boers K, Phillips C, et al. Investigation of multiple mortality events in eastern box turtles (*Terrapene carolina carolina*). PloS One. 2018;13(4). doi: 10.1371/journal.pone.0195617.

74) Beck FK, Rosenthal TC. Prealbumin: a marker for nutritional evaluation. Am Fam Physician. 2002;65: 1575-1578.

75) Zaias J, Cray C. Protein electrophoresis: A tool for the reptilian and amphibian practitioner. J Herpetol Med Surg. 2002;12(1): 30-32.

76) Rohilla MS, Tiwari PK. Simple method of blood sampling from Indian freshwater turtles for genetic studies. Acta Herpetol. 2020;3(4): 65-69.

77) Stacy NI, Alleman AR, Sayler KA. Diagnostic hematology of reptiles. Clin Lab Med. 2011;31(1): 87-108. doi: 10.1016/j.cll.2010.10.006.

78) Anderson ET, Minter LJ, Clarke EO, Mroch RM, Beasley JF, Harms CA. The effects of feeding on hematological and plasma biochemical profiles in green (*Chelonia mydas*) and Kemp's ridley (*Lepidochelys kempii*) sea turtles. Vet Med Int. 2011;2011. doi: 10.4061/2011/890829.

79) Stewart K, Mitchell MA, Norton T, Krececk RC. Measuring the level of agreement in hematologic and biochemical values between blood sampling sites in leatherback sea turtles (*Dermochelys coriacea*). J Zoo Wildl Med. 2021;43(4): 719-725. doi: 10.1638/2011-0045R.1.

80) Bolten AB, Bjorndal KA. Blood profiles for a wild population of green turtles (*Chelonia mydas*) in the southern Bahamas: Size-specific and sex-specific relationships. J Wildl Dis. 1992;28(3): 407-413. doi: 10.7589/0090-3558-28.3.407.

81) Alberghina D, Marafioti S, Spadola F, Panzera M, Piccione G. Influence of short-term storage conditions on the stability of total protein concentrations and electrophoretic fractions in plasma samples from loggerhead sea turtles, *Caretta caretta*. Comp Clin Path. 2014;24(5): 1091-1095. doi:10.1007/s00580-014-2038-z.

82) Stacy NI, Chabot RM, Innis CJ, Cray C, Fraser KM, Rigano KS, et al. Plasma chemistry in nesting leatherback sea turtles (*Dermochelys coriacea*) from Florida: Understanding the importance of sample hemolysis effects on blood analytes. PloS One. 2019;14(9). doi:10.1371/journal.pone.0222426.

Supplementary File 1. Combined Data.

All project data collected upon turtle capture, which includes identifying information, temporal data, capture method, weather data, measurements, physical exam findings, and release information.

1) Deem SL, Karesh WB, Weisman W. Putting theory into practice: Wildlife health in conservation. Conserv Biol. 2008;15(5): 1224-1233. doi:10.1111/j.1523-1739.2001.00336.x.

2) Ryser-Degiorgis MP. Wildlife health investigations: needs, challenges and recommendations. BMC Vet Res. 2013;9(1): 223. doi: 10.1186/1746-6148-9-223.

3) Mörner T, Obendorf DL, Artois M, Woodford MH. Surveillance monitoring of wildlife diseases. Rev Sci Tech. 2002;21(1): 67-76. doi: 10.20506/rst.21.1.1321.

4) Flower JE, Byrd J, Cray C, Allender MC. Plasma electrophoretic profiles and hemoglobin binding protein reference intervals in the eastern box turtle (*Terrapene carolina carolina*) and influences of age, sex, season, and location. J Zoo Wildl Med. 2014;45(4): 836-842. doi: 10.1638/2014-0035.1.

Formatted: Font: Italic

5) Knotek Z, Musilova A, Pinterova K, Knotkova Z. Plasma protein electrophoresis as a diagnostic tool for endangered Asian reptiles. World Small Animal Veterinary Association World Congress Proceedings. 2015. Available from https://www.vin.com/apputil/content/defaultadv1.aspx?id=7259164&pid=14365&print=1

6) Allender MC, Junge RE, Baker-Wylie S, Hileman ET, Faust LJ, Cray C. Plasma electrophoretic profiles in the eastern massasauga (*Sistrurus catenatus*) and influences of age, sex, year, location, and snake fungal disease. J Zoo Wildl Med. 2015;46(4): 767-773. doi: 10.1638/2015-0034.1.

Formatted: Font: Italic

7) Cooper-Bailey K, Smith SA, Zimmerman K, Lane R, Raskin RE, Denardo D. Hematology, leukocyte cytochemical analysis, plasma biochemistry, and plasma electrophoresis of wild-caught and captive-bred Gila monsters (*Heloderma suspectum*). Vet Clin Pathol. 2011;40(3): 316-323. doi: 10.1111/j.1939-165x.2011.00337.x.

Formatted: Font: Italic

8) Gicking JC, Foley AM, Harr KE, Raskin RE, Jacobson E. Plasma protein electrophoresis of the Atlantic loggerhead sea turtle, *Carretta carretta*. J Herpetol Med Surg. 2004;14(3): 13-18. doi: 10.5818/1529-9651.14.3.13.

9) Giménez M, Saco Y, Pato R, Busquets A, Martorell JM, Bassols A. Plasma protein electrophoresis of *Trachemys scripta* and *Iguana iguana*. Vet Clin Pathol. 2010;39(2): 227-235. doi: 10.1111/j.1939-165x.2009.00204.x.

10) Machado CC, Silva LFN, Ramos PRR, Takahira RK. (2006). Seasonal influence on hematologic values and hemoglobin electrophoresis in Brazilian *boa constrictor amarali*. J Zoo Wildl Med. 2006;37(4): 487–491. doi: 10.1638/05-124.1.

11) Proverbio D, Bagnagatti De Giorgi G, Della Pepa A, Baggiani L, Spada E, Perego R, et al. Preliminary evaluation of total protein concentration and electrophoretic protein fractions in fresh and frozen serum from wild Horned Vipers (*Vipera ammodytes ammodytes*). Veterinary Clinical Pathology. 2012;41(4): 582-586. doi: 10.1111/j.1939-165x.2012.00486.x.

12) Silva LFN, Riani-Costa CCM, Ramos PRR, Takahira RK. Seasonal influence on biochemical profile and serum protein electrophoresis for *boa constrictor amarali* in captivity. Braz J Biol. 2011;71(2): 517-520. doi: 10.1590/s1519-69842011000300023.

13) Work TM, Rameyer RA, Balazs GH, Cray C, Chang SP. Immune status of free-ranging green turtles with fibropapillomatosis from Hawaii. J Wildl Dis. 2001;37(3): 574-581. doi: 10.7589/0090-3558-37.3.574.

14) Cray, C. Acute phase proteins in animals. Prog Mol Biol Transl Sci. 2012;105: 113-150. doi:10.1016/b978-0-12-394596-9.00005-6.

Formatted: Font: Italic

Formatted: Font: Italic

Formatted: Font: Italic

Formatted: Font: Italic

Formatted: Font: Italic

~~15) Innis CJ, Tlusty M, Wunn D. Hematologic and plasma biochemical analysis of juvenile head-started northern red-bellied cooters (*Pseudemys rubriventris*). J Zoo Wildl Med. 2007;38(3): 425-432. doi:10.1638/1042-7260(2007)38[425:hapbao]2.0.co;2.~~

~~16) Rousselet E, Stacy NI, Lavictoire K, Higgins BM, Tocidlowski ME, Flanagan JP, et al. Hematology and plasma biochemistry analytes in five age groups of immature, captive-reared loggerhead sea turtles (*Caretta caretta*). J Zoo Wildl Med. 2013;44(4): 859-874. doi:10.1638/2012-0162r1.1.~~

~~17) Congdon JD, Sels RC. Relationships of reproductive traits and body size with attainment of sexual maturity and age in Blanding's turtles (*Emydoidea blandingii*). J Evol Biol. 1993;6(4): 547-557. doi:10.1046/j.1420-9101.1993.6040547.x.~~

~~18) van Dijk PP, Rhodin AGJ. *Emydoidea blandingii*. The IUCN Red List of Threatened Species. 2011. Available from https://dx.doi.org/10.2305/IUCN.UK.2011-1.RLTS.T7709A155088836.en.~~

~~19) Glowacki G. Blanding's Turtle recovery program: 2017 Summary Report. Lake County Forest Preserve District, Blanding's Turtle Recovery Program. 2017.~~

~~20) Gutzke WH, Packard GC. The influence of temperature on eggs and hatchlings of Blanding's turtles, *Emydoidea blandingii*. J Herpetol. 1987;21(2): 161. doi:10.2307/1564476.~~

~~21) Ernst CH, Lovich JE. *Emydoidea blandingii*, Blanding's Turtle. In: Turtles of the United States and Canada. 2nd ed. Baltimore: The John Hopkins University Press; 2009. pp. 233–249.~~

~~22) Mosimann JE, Bider JR. Variation, sexual dimorphism, and maturity in a Quebec population of the common snapping turtle, *Chelydra serpentina*. Can J Zool. 1960;38:19–38.~~

~~23) Newman E, Allender M, Thompson D, Glowacki G, Ivančić M, Adkesson M, et al. Measuring fat content using computed tomography to establish a body condition index in~~

Formatted: Font: Italic

Formatted: Font: Italic

Formatted: Font: Italic

Formatted: Font: Italic

Formatted: Font: Italic

Formatted: Font: Italic

Formatted: Font: Italic

free-ranging Blanding's turtles (*Emydoidea blandingii*) in Illinois. J Zoo Wildl Med. 2019;50(3): 594-603. doi: 10.1638/2018-0154.

24) R Core Team. 2020. R: A language and environment for statistical computing. R Foundation for Statistical Computing, Vienna, Austria. https://www.R-project.org/.

25) Bates D, Maechler M, Bolker B, Walker S. Fitting linear mixed-effects models using lme4. J Stat Softw. 2015;67(1): 1-48. doi:10.18637/jss.v067.i01.

26) Kuznetsova A, Brockhoff PB, Christensen RHB. lmerTest package: Tests in linear mixed effects models. J Stat Softw. 2017; 82(13): 1-26. doi: 10.18637/jss.v082.i13.

27) Lenth RV. Least-squares means: The R package ismeans. J Stat Softw. 2016;69(1): 1-33. doi: 10.18637/jss.v069.i01.

28) Fox J, Weisberg S. An {R} Companion to Applied Regression. Sage Publications, USA. 2011. Available from http://socserv.socsci.mcmaster.ca/jfox/Books/Companion.

29) Barton K. MuMIn: Multi-Model Inference. R package version 1.43.15. 2019. Available from https://CRAN.R-project.org/package=MuMIn.

30) Lüdecke D. ggeffects: Tidy data frames of marginal effects from regression models. J Open Source Softw. 2018;3(26): 772. doi: 10.21105/joss.00772.

31) Friedrichs KR, Harr KE, Freeman KP, Szladovits B, Walton RM, Barnhart KF, et al. ASVCP reference interval guidelines: determination of de novo reference intervals in veterinary species and other related topics. Vet Clin Pathol. 2012;41: 441–453. doi:10.1111/vcp.12006.

32) Walton RM. Subject-based reference values: biological variation, individuality, and reference change values. Vet Clin Pathol. 2012;41:175–181.

33) Harris EK. Effects of intra- and interindividual variation on the appropriate use of normal ranges. Clin Chem. 1974;20:1535–1542.

Formatted: Font: Italic

34) Finnegan D. referenceIntervals: Reference Intervals. R package version 1.1.1. 2004. Available from https://CRAN.R-project.org/package=referenceIntervals.

35) Horn PS, Feng L, Li Y, Pesce AJ. Effect of outliers and nonhealthy individuals on reference interval estimation. Clin Chem. 2001;47: 2137-2145.

36) Shine R. Life-history evolution in reptiles. Annu Rev Ecol Ecol Syst. 2005;36(1): 23-46. doi:10.1146/annurev.ecolsys.36.102003.152631.

37) Huey R, Slatkin M. 1976. Costs and benefits of lizard thermoregulation. Q. Rev. Biol. 51:363–84

38) Osborne AG, Jacobson ER, Bresette MJ, Singewald DA, Scarpino RA, Bolten AB. Reference intervals and relationships between health status, carapace length, body mass, and water temperature and concentrations of plasma total protein and protein electrophoretogram fractions in Atlantic loggerhead sea turtles and green turtles. J Am Vet Med Assoc. 2010;237(5): 561-567. doi:10.2460/javma.237.5.561.

39) NOAA. National Centers for Environmental Information; 2020. US Local Climatological Data. NCEI DSI 3505. Available from https://www.ncdc.noaa.gov/cdo-web/datasets/LCD/stations/WBAN:04845/detail.

40) Rozen- Rechels D, Dupoué A, Lourdais O, Chamaillé- Jammes S, Meylan S, Clobert J, et al. When water interacts with temperature: Ecological and evolutionary implications of thermo- hydroregulation in terrestrial ectotherms. Ecol Evol. 2019;9(17): 10029-10043. doi:10.1002/ece3.5440.

41) Flint M, Brand A, Bell IP, Hof CA. Monitoring the health of green turtles in northern Queensland post catastrophic events. Sci Total Environ. 2019;660: 586-592. doi:10.1016/j.scitotenv.2019.01.065.

42) Chaffin K, Norton TM, Gilardi K, Poppenga R, Jensen JB, Moler P, et al. Health assessment of free-ranging alligator snapping turtles (*Macrochelys temminckii*) in Georgia and Florida. J Wildl Dis. 2008;44(3): 670-686. doi:10.7589/0090-3558-44.3.670.

Formatted: Strikethrough

Formatted: Font: Italic

43) Deem SL, Dierenfeld ES, Sounguet GP, Alleman AR, Cray C, Poppenga RH, et al. Blood values in free-ranging nesting leatherback sea turtles (*Dermochelys coriacea*) on the coast of the Republic of Gabon. J Zoo Wildl Med. 2006;37(4): 464-471. doi:10.1638/05-102.1.

44) Deem SL, Norton TM, Mitchell M, Segars A, Alleman AR, Cray C, et al. Comparison of blood values in foraging, nesting, and stranded loggerhead turtles (*Caretta caretta*) along the coast of Georgia, USA. J Wildl Dis. 2009;45(1): 41-56. doi:10.7589/0090-3558-45.1.41.

45) Flint M, Matthews BJ, Limpus CJ, Mills PC. Establishment of reference intervals for plasma protein electrophoresis in Indo-Pacific green sea turtles, *Chelonia mydas*. Conserv Physiol. 2015;3(1). doi: 10.1093/conphys/cov037.

46) Innis C, Merigo C, Dodge K, Tlusty M, Dodge M, Sharp B, et al. Health evaluation of leatherback turtles (*Dermochelys coriacea*) in the northwestern Atlantic during direct capture and fisheries gear disentanglement. Chelonian Conserv Biol. 2010;9(2): 205-222. doi:10.2744/ccb-0838.1.

47) Page-Karjian A, Rivera S, Torres F, Diez C, Moore D, Dam RV, et al. Baseline blood values for healthy free-ranging green sea turtles (*Chelonia mydas*) in Puerto Rico. Comp Clin Path. 2014;24(3): 567-573. doi:10.1007/s00580-014-1947-1.

48) Perrault JR, Miller DL, Eads E, Johnson C, Merrill A, Thompson LJ, et al. Maternal health status correlates with nest success of leatherback sea turtles (*Dermochelys coriacea*) from Florida. PLoS One. 2012;7(2). doi:10.1371/journal.pone.0031841.

49) Andreani G, Carpenè E, Cannavacciuolo A, Girolamo ND, Ferlizza E, Isani G. Reference values for hematology and plasma biochemistry variables, and protein electrophoresis of

Formatted: Font: Italic

Formatted: Font: Italic

Formatted: Font: Italic

Formatted: Font: Italic

Formatted: Font: Italic

Formatted: Font: Italic

~~healthy Hermann's tortoises (*Testudo hermanni* ssp). Vet Clin Pathol. 2014;43(4): 573-583. doi:10.1111/vcp.12203.~~

~~50) Lindemann D, Allender M, Thompson D, Glowacki G, Newman E, Adamovicz L, et al. Epidemiology of Emydoidea herpesvirus 1 in free-ranging Blanding's turtles (*Emydoidea blandingii*) from Illinois. J Zoo Wildl Med. 2019;50(3): 547-556. doi: 10.1638/2018-0074.~~

~~51) Campbell TW. Clinical pathology of reptiles. In: Mader DR, editor. Reptile Medicine and Surgery. St. Louis: Saunders Elsevier; 2006. pp 453-470.~~

~~52) Dessauer HC. (1970). Blood chemistry of reptiles: Physiological and evolutionary aspects. In: Gans C, Parsons TS, editors. Biology of the Reptilia, Vol. 3. New York: Academic Press; 1970. pp. 1-72.~~

~~53) Dowling Z, Hartwig T, Kiviat E, Keesing F. Experimental management of nesting habitat for the Blanding's turtle (*Emydoidea blandingii*). Ecological Rest. 2010;28(2): 154-159. doi:10.3368/er.28.2.154.~~

~~54) Congdon J, Kinney O, Nagle R. Spatial ecology and core-area protection of Blanding's Turtle (*Emydoidea blandingii*). Can J Zool. 2011;89: 1098-1106. doi: 10.1139/z11-091.~~

~~55) Yang PY, Yu PH, Wu SH, Chie CH. Seasonal hematology and plasma biochemistry references range values of the yellow-marginated box turtle (*Cuora flavomarginata*). J Zoo Wildl Med. 2014;45:278-286. doi: 10.1638/2013-0125R1.1.~~

~~56) Musilová A, Knotková Z, Pinterová K, Knotek Z. Variations of plasma protein electrophoresis in healthy captive green iguanas (*Iguana iguana*). Vet Clin Pathol. 2015;44(2): 243-248. doi: 10.1111/vcp.12238.~~

~~57) Mader DR, Divers SJ. Current therapy in reptile medicine and surgery. St. Louis: Saunders Elsevier; 2004.~~

Formatted: Font: Italic

Formatted: Font: Italic

Formatted: Font: Italic

Formatted: Font: Italic

Formatted: Font: Italic

Formatted: Font: Italic

58) Selcer KW, Palmer BD. Estrogen downregulation of albumin and a 170-kDa serum protein in the turtle, *Trachemys scripta*. Gen Comp Endocrinol. 1995;97(3): 340-352. doi: 10.1006/gcen.1995.1034.

59) Harris DJ. Clinical Tests. In: Tully T, Dorrestien G, Jones A, editors. Handbook of Avian Medicine, 2nd ed. Philadelphia: Elsevier; 2009. pp. 77–84.

60) Perrault JR, Wyneken J, Page-Karjian A, Merrill A, Miller DL. Seasonal trends in nesting leatherback turtle (*Dermochelys coriacea*) serum proteins further verify capital breeding hypothesis. Conserv Physiol. 2014;2(1). doi: 10.1093/conphys/cou002.

61) Leineweber C, Öfner S, Stöhr AC, Marschang RE, Mathes K. A comparison of thyroid hormone levels and plasma capillary zone electrophoresis in red- eared sliders (*Trachemys scripta elegans*) and map turtles (*Graptemys* spp.) depending on season and sex. Vet Clin Pathol. 2019;49(1): 78-90. doi:10.1111/vcp.12838.

62) Zaias J, Norton T, Fickel A, Spratt J, Altman NH, Cray C. Biochemical and hematologic values for 18 clinically healthy radiated tortoises (*Geochelone radiata*) on St Catherines Island, Georgia. Vet Clin Pathol. 2006;35(3): 321-325. doi:10.1111/j.1939-165x.2006.tb00139.x.

63) Mumm LE, Winter JM, Andersson KE, Glowacki GA, Adamovicz LA, Allender MC. Hematology and plasma biochemistries in the Blanding's turtle (*Emydoidea blandingii*) in Lake County, Illinois. PloS One. 2019;14(11). doi: 10.1371/journal.pone.0225130.

64) Perrault JR, Schmidt JR, Walsh CJ, Yordy JE, Tucker AD. Brevetoxin exposure, superoxide dismutase activity and plasma protein electrophoretic profiles in wild-caught Kemp's ridley sea turtles (*Lepidochelys kempii*) in southwest Florida. Harmful Algae. 2014;37: 194-202. doi:10.1016/j.hal.2014.06.007.

Formatted: Font: Italic

Formatted: Font: Italic

Formatted: Font: Italic

Formatted: Font: Italic

Formatted: Font: Italic

Formatted: Font: Italic

65) Rosenberg JF, Wellehan JF, Crevasse SE, Cray C, Stacy NI. Reference intervals for erythrocyte sedimentation rate, lactate, fibrinogen, hematology, and plasma protein electrophoresis in clinically healthy captive gopher tortoises (Gopherus polyphemus). J Zoo Wildl Med. 2018;49(3): 520–527. doi: 10.1638/2017-0183.1.

66) Lamberski N, Braun J, Witte C, Christopher M, Field K, Averill-Murray R, et al. Identifying key clinical signs and validating body condition scores to minimize disease spread and maximize individual survival during desert tortoise translocations. Joint Annual Meeting for Wildlife Disease Association and European Association of Wildlife Diseases. 2012. Available at: https://www.researchgate.net/publication/271519082_Identifying_key_clinical_signs_and_validating_body_condition_scores_to_minimize_disease_spread_and_maximize_individual_survival_during_desert_tortoise_translocations.

67) Adamovicz L, Allender MC, Archer G, Rzadkowska M, Boers K, Phillips C, et al. Investigation of multiple mortality events in eastern box turtles (*Terrapene carolina carolina*). PloS One. 2018;13(4). doi: 10.1371/journal.pone.0195617.

68) Beck FK, Rosenthal TC. Prealbumin: a marker for nutritional evaluation. Am Fam Physician. 2002;65: 1575-1578.

69) Zaias J, Cray C. Protein electrophoresis: A tool for the reptilian and amphibian practitioner. J Herpetol Med Surg. 2002;12(1): 30-32.

70) Rohilla MS, Tiwari PK. Simple method of blood sampling from Indian freshwater turtles for genetic studies. Acta Herpetol. 2020;3(4): 65-69.

71) Stacy NI, Alleman AR, Sayler KA. Diagnostic hematology of reptiles. Clin Lab Med. 2011;31(1): 87-108. doi: 10.1016/j.cll.2010.10.006.

Formatted: Strikethrough

Formatted: Font: Italic

72) Alberghina D, Marafioti S, Spadola F, Panzera M, Piccione G. Influence of short-term storage conditions on the stability of total protein concentrations and electrophoretic fractions in plasma samples from loggerhead sea turtles, *Caretta caretta*. Comp Clin Path. 2014;24(5): 1091-1095. doi:10.1007/s00580-014-2038-z.

73) Stacy NI, Chabot RM, Innis CJ, Cray C, Fraser KM, Rigano KS, et al. Plasma chemistry in nesting leatherback sea turtles (*Dermochelys coriacea*) from Florida: Understanding the importance of sample hemolysis effects on blood analytes. PloS One. 2019;14(9). doi:10.1371/journal.pone.0222426.

Formatted: Font: Italic

Formatted: Font: Italic

WILDLIFE EPIDEMIOLOGY LAB

2001 S. Lincoln Ave.
Urbana, IL 61802 USA
vetmed.illinois.edu/wel

Dear Editor,

My co-authors and I wanted to express our sincere thanks for your time and effort in reviewing the following manuscript "Plasma electrophoresis profiles of Blanding's turtles (*Emydoidea blandingii*) and influences of month, age, sex, health status, and location." We have incorporated all requested changes into the revised manuscript. In addition, we have included a point-by-point explanation below. All responses are noted in ***bold-italic*** font.

Sincerely,

Kirsten Andersson

Reviewer #1: Well written manuscript. I would recommend to include also the basic hematological and plasma biochemical profile of the turtles (to really understand their health status - this is standard procedure in clinical research, as well as the control of faeces for any kind of parasites).

We appreciate the comment by the reviewer and generally agree that hematology and plasma biochemistry has traditionally been used to determine health status of wildlife. However, hematology and biochemistry data representing this population was published previously (Mumm et al., 2019) and cited in this manuscript. As determined in that manuscript, the ability of CBCs and biochemistries to determine health is difficult and does not adequately describe the health status of this species. Thus, the purpose of the current status was to establish baseline values for EPH in an effort to find a more sensitive assay for host response and health than can be provided by CBC and biochemistries.

Reviewer #2: This is a very unique and important study. It is very thorough and the statistical analyses have been performed adequately. Much useful information is presented to better understand turtle physiology. I have made a number of comments within the document: the biggest changes need to be in regards to re-working some of the figures, reference intervals by life-stage class, and showing some of the electrophoretograms for better interpretation of fractions. However, the majority of my comments are minor.

We appreciate the comment by the reviewer and have added several figures as suggested.

Paper Comments

1) **Delete "Establishing" from Line 22**

Modified as suggested.

2001 S. Lincoln Ave.
Urbana, IL 61802 USA
vetmed.illinois.edu/wel

2) **Delete "baseline values" from Line 24.**

Modified as suggested.

3) **Line 27 - This parenthetical inclusion is not useful. Either delete it, or be specific.**

Modified as suggested.

4) **Delete "swings" from Line 33**

Modified as suggested.

5) **Line 36 - Of which fractions?**

We have changed the word "concentrations" to "fractions", which matches the terminology used in previous sentences and mirrors the way the information is presented in the abstract.

6) **Line 37 - This is unclear as written.**

We changed the sentence to read, "The two sites showed a wide/large variation over the two years."

7) **Delete "baseline concentration ranges" from Line 40.**

Modified as suggested. The sentence now reads, "Establishing ranges for these analytes will allow investigation into disease prevalence and other environmental factors impacting this endangered species."

8) **Delete "aid in further" from line 40.**

Modified as suggested.

9) **Delete "studies" from Line 40**

Modified as suggested.

10) **Line 45 - Keywords should differ from those used in the title.**

Additional keywords have been added and changed to differ from those words in the title.

11) **Delete "sustainability and conservation of species" from line 48-49**

While we agree that the declarative nature of the statement might be too strong, we feel the sentiment is reflected in the reference citing this statement. As a compromise for Blanding's turtles, we have added the words "potentially affecting the" prior to the requested deleted statement.

12) **Line 52 - See: Deem et al. 2001. Putting Theory into Practice: Wildlife Health in Conservation; Aguirre and Lutz 2004. EcoHealth; Page-Karjian A, Perrault JR. 2021. Sea turtle health assessments: maximizing turtle encounters to better understand health. In Nahill B (Ed), Sea Turtle Research and Conservation: Lessons from Working in the Field, Elsevier, Academic Press, Cambridge, MA USA, pp. 31–44.**

Thank you for these reference suggestions. They have been added to the manuscript.

2001 S. Lincoln Ave.
Urbana, IL 61802 USA
vetmed.illinois.edu/wel

13) **Line 53 – note after common**

Modified as suggested.

14) **Delete “has” from Line 54**

Modified as suggested

15) **Line 55 - See Osborne et al. 2010 in green and loggerhead turtles.**

Thank you for this reference suggestion. It has been added to the manuscript.

16) **Line 56 - Aren't APPs also plasma proteins? Seems redundant.**

We agree that APPs are plasma proteins, so we have changed to sentence to eliminate redundancy.

17) **Add a comma after “stress” from Line 58.**

Modified as suggested.

18) **Delete “or” from Line 60.**

Modified as suggested.

19) **Delete “parts of” from Line 64**

Modified as suggested.

20) **Delete “parts of” from Line 65**

Modified as suggested.

21) **Delete “es” from “sexes” in Line 75**

Modified as suggested.

22) **Line 85- Which one is the managed and which one is the unmanaged site? I think you need to outline that clearly since it was one of your objectives. It is not clear by the descriptions.**

We agree that this point was not made clear. We have now stated which site is managed and which in unmanaged in lines 105 and 106.

23) **Add comma after “oak-savannah” in line 86**

Modified as suggested.

24) **Line 87 - How? If you are going to mention this, you need to elaborate slightly.**

The line has been changed to read “Management of mesopredators is also more robust in SBCP than IBSP, with studies focusing on camera trap surveillance for predator presence throughout the year as well as nest predation rates [23].”

25) **Line 90 - Some turtles are radio tagged? Might need a brief description of this and a reference to the study(ies).**

2001 S. Lincoln Ave.
Urbana, IL 61802 USA
vetmed.illinois.edu/wel

A brief description describing radiotelemetry was added, and two papers that used this methodology were referenced here. The sentence reads, "Radiotelemetry is a three-part system using a radio transmitter, a radio antenna, and a radio receiver. The transmitter is attached to the turtle's shell, which transmits a signal to the antenna and correlates to a beeping produced by the receiver that gets louder as the animal gets closer [23][24]."

26) **Line 92 - Would being trapped in a hoop net cause any kind of stress that might lead to changes in protein concentrations? 24 hours is a long time to be trapped.**

Short-term stressors have been demonstrated to change EPH values in other chelonians (Innis, et al. Health evaluation of leatherback turtles [Dermochelys coriacea] in the Northwestern Atlantic during direct capture and fisheries gear entanglement). However, the relationship has not yet been established for Blanding's turtles. The point has been added to the discussion, and the sentence reads, "For those turtles that were collect and sampled from traps, the stress of trapping could ultimately play a role in affecting plasma protein fractions [50], but the significance has not been studied in Blanding's turtles."

27) **Add comma after "traps" in line 93.**

Modified as suggested.

28) **Line 96 - How? Were the turtles tagged/notched? How were they identified?**

A description of how the turtles are assigned a permanent ID was added in the methods section. The sentence reads, "Each turtle was assigned a permanent ID, marking the shell with a notch code unless previously marked as well as inserting a pit tag (microchip) under the skin, and mass, sex, and age class were recorded."

29) **Deletes "was" from line 96.**

Modified as suggested.

30) **Add comma after "female" in line 97.**

Modified as suggested.

31) **Line 97 - There is no description of head-started turtles here? Lots of things are being mentioned in the methods without clarification (tagging, radio telemetry, head-starting, etc).**

A description of head-starting was added and referenced in the methods. The sentence reads, "Sex of head-started turtles, which are clutches deposited in captivity from free-range females, was known due to established incubation temperatures, as males were incubated at 26.5°C and females at 31.0°C [23][25]." A description of tagging has been added to the methods, and the sentence reads, "Each turtle was assigned a permanent ID, marking the shell with a notch code unless previously marked as well as inserting a pit tag (microchip) under the skin, and mass, sex, and age class were recorded. Sex was classified as male, female, or unknown." Finally, a description about radiotelemetry has been added to the methods, and the sentence reads, "Radiotelemetry is a three-part system using a radio transmitter, a radio antenna, and a radio receiver. The transmitter is attached to the turtle's shell, which transmits a signal to the

2001 S. Lincoln Ave.
Urbana, IL 61802 USA
vetmed.illinois.edu/wel

antenna and correlates to a beeping produced by the receiver that gets louder as the animal gets closer [23][24]."

32) **Line 103 - Reference needed for these mass determinations.**

These mass determinations are based off of an existing work that has been referenced in the methods: Mumm et al [24].

33) **Line 103 - What is this?**

LCFPD stands for Lake County Forest Preserve District, which is the region our research was conducted in. We have addressed this in the methods.

34) **Line 104 - When? And for this study? or a separate study? This is unclear.**

These mass determinations are based off of an existing work that has been referenced in the methods: Mumm et al [24].

35) **Line 105 - This seems more like a body condition index than a body fat percentage.**

The article referenced here used a CT scan to identify that the best determination of predicted fat percentage, especially for use in the field, was the relationship between carapace length and mass. We used predicted fat percentage as our marker in this study due to this study's recommendations.

36) **Line 109 - How did you know if they nested?**

A sentence explaining how nesting was determined has been added to line 146-147. The sentence reads, "Nesting was determined either by observation of nesting behavior or the lack of palpable eggs after having previously been confirmed gravid."

37) **Line 114 - This site is not recommended for use in turtles, due to heavy lymph contamination (and the risk of damaging spinal cord). There are numerous references on this. Jugular/brachial is preferred here. How did you account for potential lymph contamination of samples, as oftentimes it can go undetected? This would impact protein concentrations. Please address in discussion. This is adequately addressed in discussion, however.**

We agree that lymph contamination is an appropriate concern, and it is addressed in the discussion. The subcarapacial sinus is the most accessible venipuncture site to use when in the field alone. The tail vein is a poor option in Blanding's turtles due to size, and jugular or brachial venipuncture requires two people and likely sedation or anesthesia to access safely.

38) **Line 117 - Give time frame estimates of collection to processing.**

A time frame was included in the methods. The sentence now reads, "Blood samples were placed on ice in a cooler for one to five hours depending on time of collection until returning to the lab each afternoon."

39) **Line 117 - Is this acceptable? What refractometer was used? Total solids =/= total protein. Are there conversion factors for this species? Refractometers will also pick up on electrolytes and other blood analytes, especially in sick animals. How was this**

2001 S. Lincoln Ave.
Urbana, IL 61802 USA
vetmed.illinois.edu/wel

accounted for? Biuret method is preferred. I think to keep the refractometer results, you will need some citations to justify, at the least.

We used the language "total protein was estimated" to account for the knowledge that total solids can include other substances beyond protein in the blood. We agree that there are drawbacks of using the refractometer; however, we were limited with our resources in a field setting and considered this to be our best measurement. A section discussing TS vs TP has been added to the discussion and reads as follows, "There is a discrepancy between the use of TS verses total protein (TP). A refractometer is an efficient way to measure TS in a field research setting, but TS includes both plasma proteins as well as additional plasma solutes [79]. While there have been multiple studies conducted in other chelonian species that show a significant correlation between TS and TP [80][19], a direct relationship in Blanding's turtles has not been previously identified."

40) **Line 119 - What is the time frame here for storage? You can calculate this from sample collection date to analysis date and give a range.**

A time frame was included in the methods. The sentence now reads, "Whole blood samples were centrifuged at 4,185 g for 10 minutes, stored at -20°C for one to four months, and shipped on dry ice to the University of Miami at the end of the field season."

41) **Line 119 - This tagging methodology should be included above with animal collection protocols.**

The explanation of this methodology was moved the methods, and the sentence reads, "Each turtle was assigned a permanent ID, marking the shell with a notch code unless previously marked as well as inserting a pit tag (microchip) under the skin, and mass, sex, and age class were recorded."

42) **Add comma after "pit tag" in Line 120**

The sentence has been deleted.

43) **Delete "total protein" from Line 134**

Modified as suggested.

44) **Delete "AGR" from Line 135**

We changed AGR to A:G. This change has been reflected throughout the rest of the manuscript.

45) **Line 136 - It would be helpful to have a picture of an electrophoretogram (or a composite of several: juvenile, subadult, adult male, adult female, gravid female). That way we can see the cuts and follow those guidelines for future studies. There are other turtle studies by some authors on this manuscript where alpha-1 doesn't seem to be an actual fraction as there is no peak/trough to make a real cut. I would disagree with this fraction if that is the case, but would have to see the electrophoretogram first. Making these types of cuts (alpha-1) is not correct by conventional methods.**

2001 S. Lincoln Ave.
Urbana, IL 61802 USA
vetmed.illinois.edu/wel

An electrophoretogram figure has been added to the manuscript. The electrophoretogram does show a peak/trough associated with the alpha-1 fraction in this case.

46) **Add comma after) in line 145.**

Modified as suggested.

47) **Line 157 - Were some individuals sampled more than once? This was not mentioned in the methods.**

Some turtles, especially those with radio transmitters, were sampled multiple times, which was accounted for in our model. This has been addressed in the methods, and the sentence reads, "Many turtles, especially those equipped with a radio transmitter, were sampled up to two times per summer." It is also addressed in the results, which reads as, "Fifty animals were sampled two times approximately one year apart, and ten animals were sampled three times – twice within the 2018 active season and a third time in 2019."

48) **Add period after] in line 162.**

Modified as suggested.

49) **Line 172 - Hemolysis and lipemia need to be addressed in results.**

A section addressing lipemia and hemolysis have been added to the results section, and the sections reads, "Blood samples were collected from the subcarapacial sinus due to its relative ease of access and minimal restraint requirement in a field setting. Grossly hemolyzed or lymph contaminated samples were removed from the study, although microscopic hemolysis cannot be ruled out. The timing of sampling relative to food consumption is unknown, so post-prandial changes, such as lipemia, could impact results." Hemolysis and lipemia are also addressed in the discussion.

50) **Line 173 - Recaptures also need to be described in the methods.**

Recaptures have now been addressed in the methods, and the sentence now reads, "Many turtles, especially those equipped with a radio transmitter, were sampled up to two times per summer."

51) **Delete "the" from line 175.**

Modified as suggested.

52) **Line 192 - SI units (g/L) should be used throughout in figures/tables/results. Also keep number of decimal places standard throughout. You jump from 2-3.**

The SI units have been adjusted to g/L, and the decimal places have bee standardized.

53) **Line 202 - Do you really need to report absolute and relative? Absolute is really all that can be interpreted clinically; however, this can be open to discussion if the authors' feel it is necessary. Most studies just report absolute values and it gets a bit cumbersome reading about both sets of results here and in the tables.**

We feel very strongly about reporting both absolute and relative plasma protein fractions. There are many reasons why one or the other might be affected, but we feel reporting both

2001 S. Lincoln Ave.
Urbana, IL 61802 USA
vetmed.illinois.edu/wel

provides stronger evidence supporting the arguments we have presented. Additionally, absolute EPH fraction concentrations depend on TP/TS, therefore they might vary depending on which method is used to measure TP/TS. By providing values for relative concentrations as well, we ensure that our findings can be compared more readily across studies using different methods to measure TP/TS. The reasoning behind the decision to identify both is included in the discussion.

54) **Add comma after "years' in line 211.**

Modified as suggested.

55) **Line 229 - Some kind of summary is needed in these figure legends to help the reader comprehend. See comment below on suggestions to make these more clear.**

A description for the new figure 4 has been included.

56) **Line 232 - Your abbreviation for A:G is not consistent. Please ensure consistency before resubmitting. I suggest A:G.**

The abbreviation has been changed to A:G, and this change is reflected throughout the remainder of the manuscript.

57) **Line 252 - Not all of these abbreviations are defined.**

All abbreviations have been defined in the tables.

58) **Line 293 - It seems like you should do RIs for each life-stage class and gravid v non gravid? Since you found so many differences based on these parameters, they should be split up into 5 (juvenile, subadult, male, non-gravid female, gravid female).**

We did identify many statistically significant differences between sexes and age classes, but many of these differences were relatively small in magnitude (see "Difference" column in tables 1, 3 – 5) so we elected to keep data from all apparently healthy individuals in the reference interval dataset to maximize sample size. Clinicians can utilize the "differences" columns from our tables to understand roughly how much higher or lower EPH values may be for certain age classes or sexes compared to the overall population values.

59) **Line 297 - You need to keep all the number of decimal places consistent throughout. I fixed A:G as an example. Please revise for consistency and clarity.**

The tables have been edited with consistent decimal places. The abbreviation has been changed to A:G, and this change is reflected throughout the remainder of the manuscript.

60) **Line 311 - Citation(s) needed. See above suggestions in introduction. Could also cite Campbell's Clin Path Chapter in Reptile Med and Surg.**

Citations provided earlier on in the paper were included here as suggested.

61) **Delete "and observed" from Line 316.**

Modified as suggested.

2001 S. Lincoln Ave.
Urbana, IL 61802 USA
vetmed.illinois.edu/wel

62) **Line 316 - A negative correlation between A:G and what? Environmental temperature? Not enough information is presented here.**

We agree this sentence was misleading as written. The sentence now reads, "Temperature is a key determinant of metabolic rates in ectotherms, including reptiles [41][42], and previous studies in loggerheads (Caretta caretta) and green turtles (Chelonia mydas) observed a negative correlation with the A:G and environmental temperature [16]."

63) **Line 320 - Both alpha globulins? Be specific, you have two fractions.**

The sentence now reads, "Similar to the temperature-associated protein changes in sea turtles, Blanding's turtle TP, albumin, alpha 1 and alpha 2 globulins, and gamma globulin concentrations were greater in 2018, while prealbumin, beta globulin, and A:G were greater in 2019."

64) **Line 334 - You need a citation for these statements.**

We included Rozen- Rechels, et al [44] to support this statement.

65) **Delete "as well" from line 342.**

Modified as suggested.

66) **Line 342 - For some reason this paragraph seems unnecessary, or can be condensed? It isn't saying much else that the previous two paragraphs haven't said already.**

We agree that this paragraph does not provide any new information for the reader. We condensed the information we wanted to keep into the previous paragraph.

67) **Add period behind] in line 354.**

Modified as suggested.

68) **Line 356 - The only differences that remained similar? This doesn't make sense as written. This section has been hard to follow.**

We agree this sentence reads poorly. The sentence now reads, "The only consistent location-related differences between 2018 and 2019 were the cold spots at the northern end of SBCP, potentially indicating a more consistent health status for turtles occupying this area."

69) **Line 356 - Hot and cold need to be defined in the methods.**

Hot and cold spots have been defined in the methods. The sentence reads, "Hot spots are areas with higher plasma protein concentrations, while cold spots are areas with lower plasma protein concentrations."

70) **Line 371 - This contradicts your earlier paragraph where you state that gamma globulins are higher in 2018 than 2019 as 2018 was warmer. Here, gamma (although relative, not absolute), was higher in May, which would be cooler correct? This is why I'm not sure using the relative values adds much to the paper. Your subsequent**

2001 S. Lincoln Ave.
Urbana, IL 61802 USA
vetmed.illinois.edu/wel

sentences on pathogen transmission and vitellogenesis makes more sense, so perhaps just remove the temperature explanation.

We removed the temperature explanation as suggested and focused on the mating and reproductive influences on the protein fractions. We do see value in including relative and absolute fractions (see justification in our response to comment 53).

71) **Line 379 - Also see https://royalsocietypublishing.org/doi/pdf/10.1098/rsbl.2019.0248**

The suggested citation was included here.

72) **Delete "this" from Line 386.**

Modified as suggested.

73) **Delete "levels" from Line 389.**

Modified as suggested.

74) **Line 393 - Citation needed.**

We included Cray, C "Acute phase proteins in animals" [17] to support this statement.

75) **Line 404- What about size? Was there even size distribution between males and females?**

In general, male Blanding's turtles tend to be larger than females, but the difference in size between males and females was not emphasized in our study as much as the metabolic demands that differ between the two.

76) **Line 413 - There are other additional appropriate references here: https://academic.oup.com/conphys/article/8/1/coaa091/6013050 https://academic.oup.com/conphys/article/9/1/coab015/6190176?searchresult=1. The gopher tortoise paper is from wild tortoises, and is more appropriate than your captive tortoise reference.**

These references have been added, thank you.

77) **Add comma after "globulins" in line 415.**

Modified as suggested.

78) **Line 418 - This is a leatherback reference. See above suggested references for this. Innis et al. 2010 also found some differences in proteins by size in leatherbacks (can be cited in next sentence also).**

An appropriate Kemp's ridley reference has been added as suggested. The Innis et al. reference was added in support of the adult turtle statement, but due to the lack of study of juvenile leatherbacks in that manuscript, it was not included in support of the juvenile statement.

2001 S. Lincoln Ave.
Urbana, IL 61802 USA
vetmed.illinois.edu/wel

79) **Line 423 - Does higher beta and gamma necessarily indicate poor health? Seems like immunomodulation, instead of suppression, which at least indicates the immune system is working. I think you need a little more explanation here.**

This section has been edited for clarification and now reads, "The overall health status of individuals also contributed to variations in plasma protein concentrations. Higher absolute beta and gamma globulins were found in turtles with lower body fat percentage, which both increase in the presence of acute and chronic inflammation. BCS is a reliable measure of health status in other reptiles, with a good body condition score equating to better immune function and capability to fight periodic bouts of disease [71]."

80) **Delete "has proven to be a" from Line 424.**

Modified as suggested.

81) **Line 434 - Citation needed.**

We included Fuji, et al [72] support this statement.

82) **Delete "a" from Line 439.**

Modified as suggested.

83) **Line 443 - But then wouldn't we also see lower concentrations of other fractions, particularly albumin? Has pre-albumin ever been validated in chelonians (i.e. do we know if this fraction is real?).**

We compared turtles with URD to turtles without using only the prealbumin concentration. We added a section to the methods that reads, "EPH fractions were divided into the following six fractions: a fraction migrating in the prealbumin region ("prealbumin"), albumin, alpha 1 globulins, alpha 2 globulins, beta globulins, and gamma globulins." Prealbumin has never truly been validated in chelonion species, which is why we decided to add the "migrating fraction" description. It is unclear whether the migrating fraction in the prealbumin region is truly prealbumin. We have added a section addressing this in the discussion that reads, "It is important to note, however, that prealbumin has not yet been validated in chelonian species, so it is unclear if prealbumin is truly what is represented in the prealbumin region of the electrophoretogram."

84) **Delete "has to do with" from line 446.**

Modified as suggested.

85) **Line 447 - Did you also see higher concentrations of globulins?**

In this case, the changes between albumin and globulin individually were not large enough to be statistically significant, but the ratio between the two did change enough to be considered a significant change.

86) **Line 449 - temporally correct?**

Correct. There was variability in the same individual at different time points. This point has been addressed and now reads, "The population of Blanding's turtles that was studied showed a high degree of within-individual variability in EPH parameters at different points in time."

2001 S. Lincoln Ave.
Urbana, IL 61802 USA
vetmed.illinois.edu/wel

87) Line 452 - What is a "final value?"

We changed "final" to "absolute" for clarity.

88) Line 458 - Would be interesting to see fluctuations in some of these blood values in individuals across time. Could be done visually.

We agree that this would be interesting to look at; however, since we examined 14 analytes and followed many individuals over time, this would quickly become a massive figure that would be difficult to interpret. If there are specific analytes that the reviewer suggests we could highlight to reduce the complexity, we would be open to considering it.

89) Delete "they" from line 462.

The sentence has been reworded.

90) Delete "different" from line 463.

The sentence has been reworded.

91) Line 463 - The tagged turtles were diseased?

This sentence was misleading to readers. The sentence now reads, "Furthermore, turtles that could be identified more than once over the course of the sampling period were sampled every three to six weeks, which could indicate the fluctuation of an inflammatory response or antigenic stimulation over time."

92) Line 476 - Indicate hemolysis and lipemia in results.

A section addressing lipemia and hemolysis have been added to the results, which now reads, "Blood samples were collected from the subcarapacial sinus due to its relative ease of access and minimal restraint requirement in a field setting. Grossly hemolyzed or lymph contaminated samples were removed from the study, although microscopic hemolysis cannot be ruled out. The timing of sampling relative to food consumption is unknown, so post-prandial changes, such as lipemia, could impact results." Hemolysis was already addressed in the discussion, but a section addressing lipemia has been added and reads, "Timing samples to be collected pre- or post-prandial to account for lipemia is difficult to control in wildlife research, but a study conducted in Kemp's ridley and green sea turtles revealed that feeding had very minimal effects on plasma biochemical values and are therefore unlikely to alter clinical interpretation [78].

93) Add semi-colon after fractions in Line 480.

Modified as suggested.

94) Line 482 - This answers some of my comments above.

We are glad that you feel your previous comments have addressed, but all previous suggestions were included in earlier sections of the manuscript.

95) Line 492 - None of the scientific names below are italicized. Please check these for consistency.

2001 S. Lincoln Ave.
Urbana, IL 61802 USA
vetmed.illinois.edu/wel

Thank you for catching this error. All of the scientific names have been italicized in the references.

96) **Line XX - Need to convert to g/L for SI units for all figures/tables.**

All values have been converted to g/L in the figures and tables.

97) **Figure Comment - I don't think these figures are useful as presented. There is way too much going on to understand what we are supposed to be looking at. There are too many points and the colors/shapes blend together. Why not use color instead of grayscale, since PLoS is an online journal that publishes color figures for no additional cost? Could you also do some kind of smoothing so that it's not points, but instead just shows hot/cold? That might improve the understanding here. What does cold and hot spot mean? That is not described. Also, if you keep these and make the edits, I would combine into two panels (a and b) for 2018 and 2019 so they can be viewed together.**

We have edited the figures with these comments in mind. The meaning of hot and cold spots has been defined in the methods. We have represented the hot and cold spots in a clearer way and have made the figures colored for easier interpretation. Fig 3 and fig 4 have been combined so all of the panels can be viewed at once, making comparison between the two years much smoother.
