# Supplementary material for: Plasma electrophoresis profiles of Blanding’s turtles (Emydoidea blandingii) and influences of month, age, sex, health status, and location
Source: PLoS One. 2021 Oct 14;16(10):e0258397. doi: 10.1371/journal.pone.0258397 (PMC8516212; doi:10.1371/journal.pone.0258397)
Supplement: Attachment — Submitted filename: Response to Reviewers.docx [file pone.0258397.s003.docx]

Dear Editor,

My co-authors and I wanted to express our sincere thanks for your time and effort in reviewing the following manuscript “Plasma electrophoresis profiles of Blanding’s turtles (*Emydoidea blandingii*) and influences of month, age, sex, health status, and location.” We have incorporated all requested changes into the revised manuscript. In addition, we have included a point-by-point explanation below. All responses are noted in ***bold-italic*** font.

Sincerely,

Kirsten Andersson

**Reviewer #1: Well written manuscript. I would recommend to include also the basic hematological and plasma biochemical profile of the turtles (to really understand their health status - this is standard procedure in clinical research, as well as the control of faeces for any kind of parasites).**

***We appreciate the comment by the reviewer and generally agree that hematology and plasma biochemistry has traditionally been used to determine health status of wildlife. However, hematology and biochemistry data representing this population was published previously (Mumm et al., 2019) and cited in this manuscript. As determined in that manuscript, the ability of CBCs and biochemistries to determine health is difficult and does not adequately describe the health status of this species. Thus, the purpose of the current status was to establish baseline values for EPH in an effort to find a more sensitive assay for host response and health than can be provided by CBC and biochemistries.***

**Reviewer #2: This is a very unique and important study. It is very thorough and the statistical analyses have been performed adequately. Much useful information is presented to better understand turtle physiology. I have made a number of comments within the document: the biggest changes need to be in regards to re-working some of the figures, reference intervals by life-stage class, and showing some of the electrophoretograms for better interpretation of fractions. However, the majority of my comments are minor.**

***We appreciate the comment by the reviewer and have added several figures as suggested.***

Paper Comments

1) **Delete “Establishing” from Line 22**

***Modified as suggested.***

2) **Delete “baseline values” from Line 24.**

***Modified as suggested.***

3) **Line 27 - This parenthetical inclusion is not useful. Either delete it, or be specific.**

***Modified as suggested.***

4) **Delete “swings” from Line 33**

***Modified as suggested.***

5) **Line 36 - Of which fractions?**

***We have changed the word “concentrations” to “fractions”, which matches the terminology used in previous sentences and mirrors the way the information is presented in the abstract.***

6) **Line 37 - This is unclear as written.**

***We changed the sentence to read, “The two sites showed a wide/large variation over the two years.”***

7) **Delete “baseline concentration ranges” from Line 40.**

***Modified as suggested. The sentence now reads, “Establishing ranges for these analytes will allow investigation into disease prevalence and other environmental factors impacting this endangered species.”***

8) **Delete “aid in further” from line 40.**

***Modified as suggested.***

9) **Delete “studies” from Line 40**

***Modified as suggested.***

10) **Line 45 - Keywords should differ from those used in the title.**

***Additional keywords have been added and changed to differ from those words in the title.***

11) **Delete “sustainability and conservation of species” from line 48-49**

***While we agree that the declarative nature of the statement might be too strong, we feel the sentiment is reflected in the reference citing this statement. As a compromise for Blanding’s turtles, we have added the words “potentially affecting the” prior to the requested deleted statement.***

12) **Line 52 - See: Deem et al. 2001. Putting Theory into Practice: Wildlife Health in Conservation; Aguirre and Lutz 2004. EcoHealth; Page-Karjian A, Perrault JR. 2021. Sea turtle health assessments: maximizing turtle encounters to better understand health. In Nahill B (Ed), Sea Turtle Research and Conservation: Lessons from Working in the Field, Elsevier, Academic Press, Cambridge, MA USA, pp. 31–44.**

***Thank you for these reference suggestions. They have been added to the manuscript.***

13) **Line 53 – note after common**

***Modified as suggested.***

14) **Delete “has” from Line 54**

***Modified as suggested***

15) **Line 55 - See Osborne et al. 2010 in green and loggerhead turtles.**

***Thank you for this reference suggestion. It has been added to the manuscript.***

16) **Line 56 - Aren't APPs also plasma proteins? Seems redundant.**

***We agree that APPs are plasma proteins, so we have changed to sentence to eliminate redundancy.***

17) **Add a comma after “stress” from Line 58.**

***Modified as suggested.***

18) **Delete “or” from Line 60.**

***Modified as suggested.***

19) **Delete “parts of” from Line 64**

***Modified as suggested.***

20) **Delete “parts of” from Line 65**

***Modified as suggested.***

21) **Delete “es” from “sexes” in Line 75**

***Modified as suggested.***

22) **Line 85- Which one is the managed and which one is the unmanaged site? I think you need to outline that clearly since it was one of your objectives. It is not clear by the descriptions.**

***We agree that this point was not made clear. We have now stated which site is managed and which in unmanaged in lines 105 and 106.***

23) **Add comma after “oak-savannah” in line 86**

***Modified as suggested.***

24) **Line 87 - How? If you are going to mention this, you need to elaborate slightly.**

***The line has been changed to read “Management of mesopredators is also more robust in SBCP than IBSP, with studies focusing on camera trap surveillance for predator presence throughout the year as well as nest predation rates [23].”***

25) **Line 90 - Some turtles are radio tagged? Might need a brief description of this and a reference to the study(ies).**

***A brief description describing radiotelemetry was added, and two papers that used this methodology were referenced here. The sentence reads, “Radiotelemetry is a three-part system using a radio transmitter, a radio antenna, and a radio receiver. The transmitter is attached to the turtle’s shell, which transmits a signal to the antenna and correlates to a beeping produced by the receiver that gets louder as the animal gets closer [23][24].”***

26) **Line 92 - Would being trapped in a hoop net cause any kind of stress that might lead to changes in protein concentrations? 24 hours is a long time to be trapped.**

***Short-term stressors have been demonstrated to change EPH values in other chelonians (Innis, et al. Health evaluation of leatherback turtles [Dermochelys coriacea] in the Northwestern Atlantic during direct capture and fisheries gear entanglement). However, the relationship has not yet been established for Blanding’s turtles. The point has been added to the discussion, and the sentence reads, “For those turtles that were collect and sampled from traps, the stress of trapping could ultimately play a role in affecting plasma protein fractions [50], but the significance has not been studied in Blanding’s turtles.”***

27) **Add comma after “traps” in line 93.**

***Modified as suggested.***

28) **Line 96 - How? Were the turtles tagged/notched? How were they identified?**

***A description of how the turtles are assigned a permanent ID was added in the methods section. The sentence reads, “Each turtle was assigned a permanent ID, marking the shell with a notch code unless previously marked as well as inserting a pit tag (microchip) under the skin, and mass, sex, and age class were recorded.”***

29) **Deletes “was” from line 96.**

***Modified as suggested.***

30) **Add comma after “female” in line 97.**

***Modified as suggested.***

31) **Line 97 - There is no description of head-started turtles here? Lots of things are being mentioned in the methods without clarification (tagging, radio telemetry, head-starting, etc).**

***A description of head-starting was added and referenced in the methods. The sentence reads, “Sex of head-started turtles, which are clutches deposited in captivity from free-range females, was known due to established incubation temperatures, as males were incubated at 26.5°C and females at 31.0°C [23][25].” A description of tagging has been added to the methods, and the sentence reads, “Each turtle was assigned a permanent ID, marking the shell with a notch code unless previously marked as well as inserting a pit tag (microchip) under the skin, and mass, sex, and age class were recorded. Sex was classified as male, female, or unknown.” Finally, a description about radiotelemetry has been added to the methods, and the sentence reads, “Radiotelemetry is a three-part system using a radio transmitter, a radio antenna, and a radio receiver. The transmitter is attached to the turtle’s shell, which transmits a signal to the antenna and correlates to a beeping produced by the receiver that gets louder as the animal gets closer [23][24].”***

32) **Line 103 - Reference needed for these mass determinations.**

***These mass determinations are based off of an existing work that has been referenced in the methods: Mumm et al [24].***

33) **Line 103 - What is this?**

***LCFPD stands for Lake County Forest Preserve District, which is the region our research was conducted in. We have addressed this in the methods.***

34) **Line 104 - When? And for this study? or a separate study? This is unclear.**

***These mass determinations are based off of an existing work that has been referenced in the methods: Mumm et al [24].***

35) **Line 105 - This seems more like a body condition index than a body fat percentage.**

***The article referenced here used a CT scan to identify that the best determination of predicted fat percentage, especially for use in the field, was the relationship between carapace length and mass. We used predicted fat percentage as our marker in this study due to this study’s recommendations.***

36) **Line 109 - How did you know if they nested?**

***A sentence explaining how nesting was determined has been added to line 146-147. The sentence reads, “Nesting was determined either by observation of nesting behavior or the lack of palpable eggs after having previously been confirmed gravid.”***

37) **Line 114 - This site is not recommended for use in turtles, due to heavy lymph contamination (and the risk of damaging spinal cord). There are numerous references on this. Jugular/brachial is preferred here. How did you account for potential lymph contamination of samples, as oftentimes it can go undetected? This would impact protein concentrations. Please address in discussion. This is adequately addressed in discussion, however.**

***We agree that lymph contamination is an appropriate concern, and it is addressed in the discussion. The subcarapacial sinus is the most accessible venipuncture site to use when in the field alone. The tail vein is a poor option in Blanding’s turtles due to size, and jugular or brachial venipuncture requires two people and likely sedation or anesthesia to access safely.***

38) **Line 117 - Give time frame estimates of collection to processing.**

***A time frame was included in the methods. The sentence now reads, “Blood samples were placed on ice in a cooler for one to five hours depending on time of collection until returning to the lab each afternoon.”***

39) **Line 117 - Is this acceptable? What refractometer was used? Total solids =/= total protein. Are there conversion factors for this species? Refractometers will also pick up on electrolytes and other blood analytes, especially in sick animals. How was this accounted for? Biuret method is preferred. I think to keep the refractometer results, you will need some citations to justify, at the least.**

***We used the language “total protein was estimated” to account for the knowledge that total solids can include other substances beyond protein in the blood. We agree that there are drawbacks of using the refractometer; however, we were limited with our resources in a field setting and considered this to be our best measurement. A section discussing TS vs TP has been added to the discussion and reads as follows, “There is a discrepancy between the use of TS verses total protein (TP). A refractometer is an efficient way to measure TS in a field research setting, but TS includes both plasma proteins as well as additional plasma solutes [79]. While there have been multiple studies conducted in other chelonian species that show a significant correlation between TS and TP [80][19], a direct relationship in Blanding’s turtles has not been previously identified.”***

40) **Line 119 - What is the time frame here for storage? You can calculate this from sample collection date to analysis date and give a range.**

***A time frame was included in the methods. The sentence now reads, “Whole blood samples were centrifuged at 4,185 g for 10 minutes, stored at -20°C for one to four months, and shipped on dry ice to the University of Miami at the end of the field season.”***

41) **Line 119 - This tagging methodology should be included above with animal collection protocols.**

***The explanation of this methodology was moved the methods, and the sentence reads, “Each turtle was assigned a permanent ID, marking the shell with a notch code unless previously marked as well as inserting a pit tag (microchip) under the skin, and mass, sex, and age class were recorded.”***

42) **Add comma after “pit tag” in Line 120**

***The sentence has been deleted.***

43) **Delete “total protein” from Line 134**

***Modified as suggested.***

44) **Delete “AGR” from Line 135**

***We changed AGR to A:G. This change has been reflected throughout the rest of the manuscript.***

45) **Line 136 - It would be helpful to have a picture of an electrophoretogram (or a composite of several: juvenile, subadult, adult male, adult female, gravid female). That way we can see the cuts and follow those guidelines for future studies. There are other turtle studies by some authors on this manuscript where alpha-1 doesn't seem to be an actual fraction as there is no peak/trough to make a real cut. I would disagree with this fraction if that is the case, but would have to see the electrophoretogram first. Making these types of cuts (alpha-1) is not correct by conventional methods.**

***An electrophoretogram figure has been added to the manuscript. The electrophoretogram does show a peak/trough associated with the alpha-1 fraction in this case.***

46) **Add comma after ) in line 145.**

***Modified as suggested.***

47) **Line 157 - Were some individuals sampled more than once? This was not mentioned in the methods.**

***Some turtles, especially those with radio transmitters, were sampled multiple times, which was accounted for in our model. This has been addressed in the methods, and the sentence reads, “Many turtles, especially those equipped with a radio transmitter, were sampled up to two times per summer.” It is also addressed in the results, which reads as, “Fifty animals were sampled two times approximately one year apart, and ten animals were sampled three times – twice within the 2018 active season and a third time in 2019.”***

48) **Add period after ] in line 162.**

***Modified as suggested.***

49) **Line 172 - Hemolysis and lipemia need to be addressed in results.**

***A section addressing lipemia and hemolysis have been added to the results section, and the sections reads, “Blood samples were collected from the subcarapacial sinus due to its relative ease of access and minimal restraint requirement in a field setting. Grossly hemolyzed or lymph contaminated samples were removed from the study, although microscopic hemolysis cannot be ruled out. The timing of sampling relative to food consumption is unknown, so post-prandial changes, such as lipemia, could impact results.” Hemolysis and lipemia are also addressed in the discussion.***

50) **Line 173 - Recaptures also need to be described in the methods.**

***Recaptures have now been addressed in the methods, and the sentence now reads, “Many turtles, especially those equipped with a radio transmitter, were sampled up to two times per summer.”***

51) **Delete “the” from line 175.**

***Modified as suggested.***

52) **Line 192 - SI units (g/L) should be used throughout in figures/tables/results. Also keep number of decimal places standard throughout. You jump from 2-3.**

***The SI units have been adjusted to g/L, and the decimal places have bee standardized.***

53) **Line 202 - Do you really need to report absolute and relative? Absolute is really all that can be interpreted clinically; however, this can be open to discussion if the authors' feel it is necessary. Most studies just report absolute values and it gets a bit cumbersome reading about both sets of results here and in the tables.**

***We feel very strongly about reporting both absolute and relative plasma protein fractions. There are many reasons why one or the other might be affected, but we feel reporting both provides stronger evidence supporting the arguments we have presented. Additionally, absolute EPH fraction concentrations depend on TP/TS, therefore they might vary depending on which method is used to measure TP/TS. By providing values for relative concentrations as well, we ensure that our findings can be compared more readily across studies using different methods to measure TP/TS. The reasoning behind the decision to identify both is included in the discussion.***

54) **Add comma after “years’ in line 211.**

***Modified as suggested.***

55) **Line 229 - Some kind of summary is needed in these figure legends to help the reader comprehend. See comment below on suggestions to make these more clear.**

***A description for the new figure 4 has been included.***

56) **Line 232 - Your abbreviation for A:G is not consistent. Please ensure consistency before resubmitting. I suggest A:G.**

***The abbreviation has been changed to A:G, and this change is reflected throughout the remainder of the manuscript.***

57) **Line 252 - Not all of these abbreviations are defined.**

***All abbreviations have been defined in the tables.***

58) **Line 293 - It seems like you should do RIs for each life-stage class and gravid v non gravid? Since you found so many differences based on these parameters, they should be split up into 5 (juvenile, subadult, male, non-gravid female, gravid female).**

***We did identify many statistically significant differences between sexes and age classes, but many of these differences were relatively small in magnitude (see “Difference” column in tables 1, 3 – 5) so we elected to keep data from all apparently healthy individuals in the reference interval dataset to maximize sample size. Clinicians can utilize the “differences” columns from our tables to understand roughly how much higher or lower EPH values may be for certain age classes or sexes compared to the overall population values.***

59) **Line 297 - You need to keep all the number of decimal places consistent throughout. I fixed A:G as an example. Please revise for consistency and clarity.**

***The tables have been edited with consistent decimal places. The abbreviation has been changed to A:G, and this change is reflected throughout the remainder of the manuscript.***

60) **Line 311 - Citation(s) needed. See above suggestions in introduction. Could also cite Campbell's Clin Path Chapter in Reptile Med and Surg.**

***Citations provided earlier on in the paper were included here as suggested.***

61) **Delete “and observed” from Line 316.**

***Modified as suggested.***

62) **Line 316 - A negative correlation between A:G and what? Environmental temperature? Not enough information is presented here.**

***We agree this sentence was misleading as written. The sentence now reads, “Temperature is a key determinant of metabolic rates in ectotherms, including reptiles [41][42], and previous studies in loggerheads (Caretta caretta) and green turtles (Chelonia mydas) observed a negative correlation with the A:G and environmental temperature [16].”***

63) **Line 320 - Both alpha globulins? Be specific, you have two fractions.**

***The sentence now reads, “Similar to the temperature-associated protein changes in sea turtles, Blanding’s turtle TP, albumin, alpha 1 and alpha 2 globulins, and gamma globulin concentrations were greater in 2018, while prealbumin, beta globulin, and A:G were greater in 2019.”***

64) **Line 334 - You need a citation for these statements.**

***We included Rozen‐Rechels, et al [44] to support this statement.***

65) **Delete “as well” from line 342.**

***Modified as suggested.***

66) **Line 342 - For some reason this paragraph seems unnecessary, or can be condensed? It isn't saying much else that the previous two paragraphs haven't said already.**

***We agree that this paragraph does not provide any new information for the reader. We condensed the information we wanted to keep into the previous paragraph.***

67) **Add period behind ] in line 354.**

***Modified as suggested.***

68) **Line 356 - The only differences that remained similar? This doesn't make sense as written. This section has been hard to follow.**

***We agree this sentence reads poorly. The sentence now reads, “The only consistent location-related differences between 2018 and 2019 were the cold spots at the northern end of SBCP, potentially indicating a more consistent health status for turtles occupying this area.”***

69) **Line 356 - Hot and cold need to be defined in the methods.**

***Hot and cold spots have been defined in the methods. The sentence reads, “Hot spots are areas with higher plasma protein concentrations, while cold spots are areas with lower plasma protein concentrations.”***

70) **Line 371 - This contradicts your earlier paragraph where you state that gamma globulins are higher in 2018 than 2019 as 2018 was warmer. Here, gamma (although relative, not absolute), was higher in May, which would be cooler correct? This is why I'm not sure using the relative values adds much to the paper. Your subsequent sentences on pathogen transmission and vitellogenesis makes more sense, so perhaps just remove the temperature explanation.**

***We removed the temperature explanation as suggested and focused on the mating and reproductive influences on the protein fractions. We do see value in including relative and absolute fractions (see justification in our response to comment 53).***

71) **Line 379 - Also see https://royalsocietypublishing.org/doi/pdf/10.1098/rsbl.2019.0248**

***The suggested citation was included here.***

72) **Delete “this” from Line 386.**

***Modified as suggested.***

73) **Delete “levels” from Line 389.**

***Modified as suggested.***

74) **Line 393 - Citation needed.**

***We included Cray, C “Acute phase proteins in animals” [17] to support this statement.***

75) **Line 404- What about size? Was there even size distribution between males and females?**

***In general, male Blanding’s turtles tend to be larger than females, but the difference in size between males and females was not emphasized in our study as much as the metabolic demands that differ between the two.***

76) **Line 413 - There are other additional appropriate references here: https://academic.oup.com/conphys/article/8/1/coaa091/6013050**

[**https://academic.oup.com/conphys/article/9/1/coab015/6190176?searchresult=1**](https://academic.oup.com/conphys/article/9/1/coab015/6190176?searchresult=1)**. The gopher tortoise paper is from wild tortoises, and is more appropriate than your captive tortoise reference.**

***These references have been added, thank you.***

77) **Add comma after “globulins” in line 415.**

***Modified as suggested.***

78) **Line 418 - This is a leatherback reference. See above suggested references for this. Innis et al. 2010 also found some differences in proteins by size in leatherbacks (can be cited in next sentence also).**

***An appropriate Kemp’s ridley reference has been added as suggested. The Innis et al. reference was added in support of the adult turtle statement, but due to the lack of study of juvenile leatherbacks in that manuscript, it was not included in support of the juvenile statement.***

79) **Line 423 - Does higher beta and gamma necessarily indicate poor health? Seems like immunomodulation, instead of suppression, which at least indicates the immune system is working. I think you need a little more explanation here.**

***This section has been edited for clarification and now reads, “The overall health status of individuals also contributed to variations in plasma protein concentrations. Higher absolute beta and gamma globulins were found in turtles with lower body fat percentage, which both increase in the presence of acute and chronic inflammation. BCS is a reliable measure of health status in other reptiles, with a good body condition score equating to better immune function and capability to fight periodic bouts of disease [71].”***

80) **Delete “has proven to be a” from Line 424.**

***Modified as suggested.***

81) **Line 434 - Citation needed.**

***We included Fuji, et al [72] support this statement.***

82) **Delete “a” from Line 439.**

***Modified as suggested.***

83) **Line 443 - But then wouldn't we also see lower concentrations of other fractions, particularly albumin? Has pre-albumin ever been validated in chelonians (i.e. do we know if this fraction is real?).**

***We compared turtles with URD to turtles without using only the prealbumin concentration. We added a section to the methods that reads, “EPH fractions were divided into the following six fractions: a fraction migrating in the prealbumin region ("prealbumin"), albumin, alpha 1 globulins, alpha 2 globulins, beta globulins, and gamma globulins.” Prealbumin has never truly been validated in chelonion species, which is why we decided to add the “migrating fraction” description. It is unclear whether the migrating fraction in the prealbumin region is truly prealbumin. We have added a section addressing this in the discussion that reads, “It is important to note, however, that prealbumin has not yet been validated in chelonian species, so it is unclear if prealbumin is truly what is represented in the prealbumin region of the electrophoretogram.”***

84) **Delete “has to do with” from line 446.**

***Modified as suggested.***

85) **Line 447 - Did you also see higher concentrations of globulins?**

***In this case, the changes between albumin and globulin individually were not large enough to be statistically significant, but the ratio between the two did change enough to be considered a significant change.***

86) **Line 449 - temporally correct?**

***Correct. There was variability in the same individual at different time points. This point has been addressed and now reads, “The population of Blanding’s turtles that was studied showed a high degree of within-individual variability in EPH parameters at different points in time.”***

87) **Line 452 - What is a "final value?"**

***We changed “final” to “absolute” for clarity.***

88) **Line 458 - Would be interesting to see fluctuations in some of these blood values in individuals across time. Could be done visually.**

***We agree that this would be interesting to look at; however, since we examined 14 analytes and followed many individuals over time, this would quickly become a massive figure that would be difficult to interpret. If there are specific analytes that the reviewer suggests we could highlight to reduce the complexity, we would be open to considering it.***

89) **Delete “they” from line 462.**

***The sentence has been reworded.***

90) **Delete “different” from line 463.**

***The sentence has been reworded.***

91) **Line 463 - The tagged turtles were diseased?**

***This sentence was misleading to readers. The sentence now reads, “Furthermore, turtles that could be identified more than once over the course of the sampling period were sampled every three to six weeks, which could indicate the fluctuation of an inflammatory response or antigenic stimulation over time.”***

92) **Line 476 - Indicate hemolysis and lipemia in results.**

***A section addressing lipemia and hemolysis have been added to the results, which now reads, “Blood samples were collected from the subcarapacial sinus due to its relative ease of access and minimal restraint requirement in a field setting. Grossly hemolyzed or lymph contaminated samples were removed from the study, although microscopic hemolysis cannot be ruled out. The timing of sampling relative to food consumption is unknown, so post-prandial changes, such as lipemia, could impact results.” Hemolysis was already addressed in the discussion, but a section addressing lipemia has been added and reads, “Timing samples to be collected pre- or post-prandial to account for lipemia is difficult to control in wildlife research, but a study conducted in Kemp’s ridley and green sea turtles revealed that feeding had very minimal effects on plasma biochemical values and are therefore unlikely to alter clinical interpretation [78].***

93) **Add semi-colon after fractions in Line 480.**

***Modified as suggested.***

94) **Line 482 - This answers some of my comments above.**

***We are glad that you feel your previous comments have addressed, but all previous suggestions were included in earlier sections of the manuscript.***

95) **Line 492 - None of the scientific names below are italicized. Please check these for consistency.**

***Thank you for catching this error. All of the scientific names have been italicized in the references.***

96) **Line XX - Need to convert to g/L for SI units for all figures/tables.**

***All values have been converted to g/L in the figures and tables.***

97) **Figure Comment - I don't think these figures are useful as presented. There is way too much going on to understand what we are supposed to be looking at. There are too many points and the colors/shapes blend together. Why not use color instead of grayscale, since PLoS is an online journal that publishes color figures for no additional cost? Could you also do some kind of smoothing so that it's not points, but instead just shows hot/cold? That might improve the understanding here. What does cold and hot spot mean? That is not described. Also, if you keep these and make the edits, I would combine into two panels (a and b) for 2018 and 2019 so they can be viewed together.**

***We have edited the figures with these comments in mind. The meaning of hot and cold spots has been defined in the methods. We have represented the hot and cold spots in a clearer way and have made the figures colored for easier interpretation. Fig 3 and fig 4 have been combined so all of the panels can be viewed at once, making comparison between the two years much smoother.***
